# Photochromic Oxime Directing Groups for Spatially Controlled Pd-Catalyzed C–H Difunctionalization with Tandem Electrophiles

**DOI:** 10.1021/acscatal.5c05469

**Published:** 2025-09-23

**Authors:** Mahmoud R. Saleh, Mahmoud Afrasi, Ajay H. Bansode, Poulami Ghosh, Dan E. Wise, Marvin Parasram

**Affiliations:** Department of Chemistry, 5894 New York University, 24 Waverly Place, third floor, New York, New York 10003, United States

**Keywords:** C−H functionalization, oximes, photochromism, photocatalysis, palladium catalysis, hypervalent iodine

## Abstract

Herein, we report the employment of oxime ethers as photochromic directing groups for the controlled functionalization of spatially and inherently distinct C­(sp^2^)–H/C­(sp^3^)–H bonds. Our approach features a semi-two-pot protocol for Pd-catalyzed C­(sp^2^)–H oxygenation, photoisomerization, and Pd-catalyzed C­(sp^3^)–H arylation for the synthesis of difunctionalized oxime derivatives in a highly selective and controlled manner. Notably, we illustrate the rare utilization of hypervalent iodine reagents as tandem electrophiles for C–H difunctionalization. The reverse sequence of functionalization events can be achieved thermally, thereby providing a platform for full directing group-controlled C–H difunctionalization. Overall, this work highlights that photochromic directing groups can provide an avenue for positionally controlled C–H difunctionalization.

## Graphical abstract

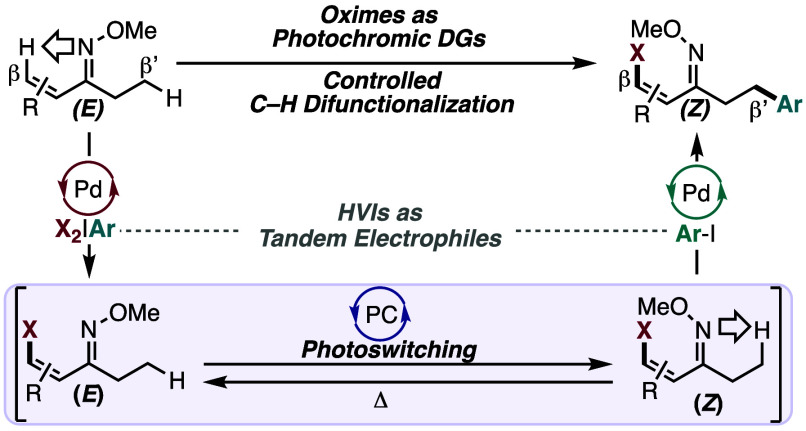

C–H bonds are ubiquitous in most organic molecules. Thus, the functionalization of C–H bonds, especially with transition-metal (TM) catalysts, has become a burgeoning area in synthetic chemistry.^1^ Numerous examples of late-stage C–H functionalization reactions have been implemented in the synthesis of a range of bioactive and natural products.^2^ Existing approaches rely on employing directing groups (DGs) for site-selective C–H functionalization.^3^ There are many classes of DG types employed for C–H functionalization based on the nature of the activated C–H bond and the synthetic utility of the transformation. DGs can either be monodentate^3^ or bidentate,^4^ possess strong or weak chelation,^5^ removable^6^ or transient,^7^ and/or templated.^8^ In most cases, DGs are flat and rigid, which restricts directionality control and enables predisposed C–H sites to undergo functionalization. For most directed C–H functionalization processes, the DG binds to the transition metal to enable proximal C–H bond activation through either direct insertion or a concerted metalation-deprotonation (CMD) mechanism (Scheme 1A, top).^9^ The formed metallacycle intermediate then undergoes functionalization through reaction with an electrophile (R–X), or via transmetalation with nucleophiles, resulting in the C–H functionalized product. However, this becomes increasingly difficult for the 2-fold functionalization of spatially distinct C–H bonds when different electrophiles are present with a singular DG (Scheme 1A, bottom). This often leads to a statistical mixture of C–H functionalized products due to a lack of DG control.^10^ While recent reports have demonstrated that C–H difunctionalization can be achieved selectively by altering the DG identity or relying on steric effects after the first C–H functionalization event, controlling the DG directionality for C–H functionalization remains an unresolved challenge.^11^

**Scheme 1:**
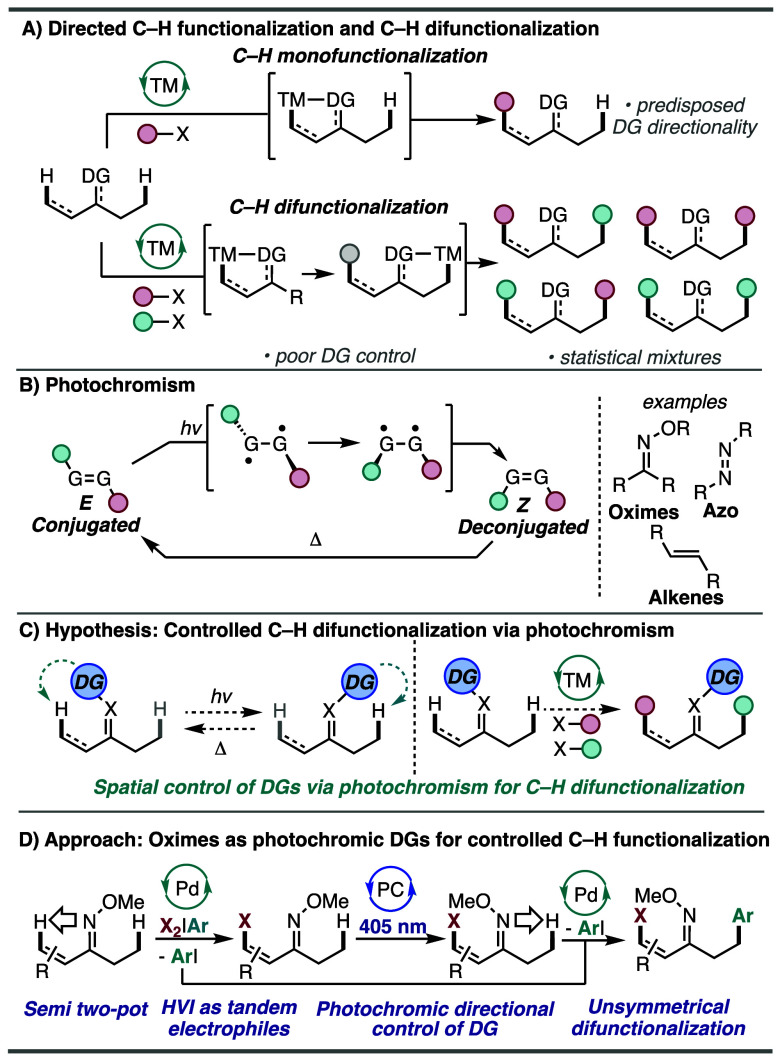
Directed C–H Functionalization and Photochromism

Photochromism is the ability to promote reversible structural changes of conjugated organic molecules at different wavelengths of light (Scheme 1B).^12^ Based on this phenomenon, we hypothesize that a DG with inherent photochromic features could engender spatial control for unsymmetrical, directed C–H difunctionalization under visible light and/or with a thermal stimulus (Scheme 1C). The ability to control the directionality of the DG would permit controlled, site-selective C–H functionalization of spatially distinct C–H bonds and empower multifunctionalization of organic molecules effectively. Common photochromic groups include azobenzene, stilbene derivatives, spirooxazines, and oximes.^13^ Seminal works by Shaw, Sanford, and others illustrated that oximes are effective directing groups for C–H functionalization.^14^ However, they are seldom used as photochromic groups in organic synthesis owing to the high energy required to trigger photoswitching of the oxime stereoisomers. Padwa and Albrecht reported low stereoisomeric ratios in the photostationary state, and deleterious N–O bond cleavage of the oxime was observed under UV-irradiation.^13e,13f^ Recently, Rovis and co-workers reported that triplet sensitization with an Ir-photocatalyst under visible light could enable one-way photoisomerization of aryl oximes with up to >20:1 *Z*:*E* ratios.^15^ Inspired by these reports, we postulated that oxime ethers could be employed as a photochromic DG to facilitate two separate Pd-catalyzed C–H functionalization events of spatially different C­(sp^2^)–H and C­(sp^3^)–H bonds with readily available hypervalent iodine (HVI) reagents as tandem electrophiles to generate difunctionalized oxime derivatives (Scheme 1D). During the development of this project, Yoshino, Matsunaga, and co-workers reported the discrete C­(sp^2^)–H and C­(sp^3^)–H functionalization of oximes, enabled by Ir-catalysis, an Ir-photosensitizer, and sequential electrophiles.^16^ While complementary, our approach features a practical workflow with inexpensive reagents. Moreover, the C–H difunctionalization event, featuring C­(sp^2^)–H oxygenation and C­(sp^3^)–H arylation, occurs sequentially in a semi-two-pot fashion with excellent stereocontrol and chemoselectivity.

We began our optimization studies of each individual step – Pd-catalyzed C­(sp^2^)–H oxygenation, photoisomerization, and Pd-catalyzed C­(sp^3^)–H arylation, with oxime substrate **(** *E* **)-1a** (Tables S1–S3). Based on the geometry of **1a**, it is predisposed to functionalize the ortho C­(sp^2^)–H bond of the aromatic ring. It was found that exposure of **(** *E* **)-1a** to modified literature conditions for Pd-catalyzed acetoxylation conditions with PIDA (3.0 equiv) in HFIP [0.2 M] was successful in generating the desired product in a 71% NMR yield.^17^ Next, the photoswitching step was achieved with thioxanthone (TX) photosensitizer in quantitative yield (Table S1), and the final C­(sp^3^)–H arylation step was achieved with Pd­(OAc)_2_, AgTFA, and a pyridone ligand (Ligand **L**) (Tables S2–S3). After optimization of each step, we carried out the optimization studies through a more practical semi-two-pot protocol for the C–H difunctionalization with oxime substrate **(** *E* **)-1a** (Table 1). After the first C­(sp^2^)–H acetoxylation step, the crude reaction mixture was subjected to photoisomerization with an organophotosensitizer (TX) with MeOH as cosolvent,^18^ leading to the *Z*-oxime intermediate (99% NMR yield) after filtration and concentration (no silica gel purification). The new conformation can now allow C­(sp^3^)–H functionalization to occur with the iodobenzene byproduct produced from the first functionalization event. New Pd­(OAc)_2_, AgTFA, and ligand **L** were added to the reaction flask to enable the Pd-catalyzed C­(sp^3^)–H arylation of the *Z*-oxime substrate, resulting in **(** *Z* **)-2a** (74% NMR yield). To the best of our knowledge, this outcome represents the first example of PIDA serving as a tandem electrophile for C–H functionalization reactions. Attempts to recycle the Pd-catalyst after the first step, however, resulted in Pd-black formation, leading to inconsistent results (Table S4). Overall, this semi-two-pot protocol resulted in the desired difunctionalized oxime product **2a** in 53% NMR yield over three transformative steps (Table 1, entry 1). Decreasing the PIDA equivalents led to a decrease in the yield of **2a** with concomitant **2a’** from the first step (entry 3). Attempts to suppress **2a’** formation by lowering the temperature, reducing the reaction time, and increasing concentration were unsuccessful (Table S5). Employment of HFIP as solvent for all steps was unsuccessful, as the photoisomerization was inefficient (entry 4). Fortunately, we found that a mixture of HFIP/MeOH (1:1) worked well for the photoisomerization step with TX. Screening various photosensitizers for the second step resulted in a lower yield of **2a** (entries 5–7). Ligand **L** was found to be crucial for the third step of the reaction; other common ligands for Pd-catalyzed C­(sp^3^)–H arylation were less efficient (entry 8 and Table S2).^19^ Control studies indicated that all the reaction components are necessary for the controlled difunctionalization of **1a (**entries 9–12).

**Table 1:**
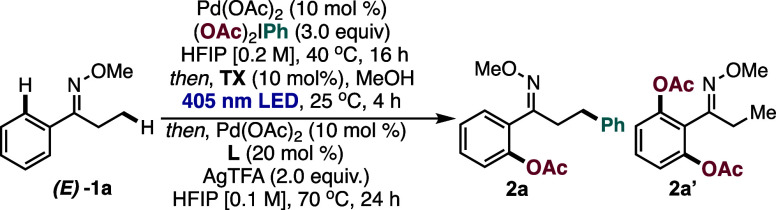
Optimization of the Reaction Parameters

Entry	Deviation from standard conditions	**2a** NMR Yield, %^a^	2a’ NMR Yield, %^a^
1	None	**53**	10
2	1.5 equiv of PIDA	21	9
3	2.0 equiv of PIDA	29	12
4	HFIP [0.10 M]^b^	0	20
5	with [Ir]^c^ ^,^ ^d^	46	12
6	with Benzil^d^	0	7
7	with 4CzIPN^d^	13	17
8	**L1**	33	11
9	No Pd	0	0
10	No light	0	13
11	No PC	0	8
12	No AgTFA	0	<5

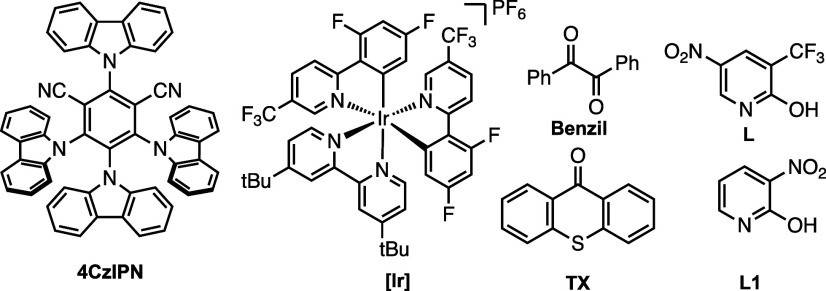

a ^1^H NMR yield using CH_2_Br_2_ as an internal standard.

b HFIP was used as solvent for all three steps.

c 1 mol % loading.

d 427 nm was used.

After uncovering the optimized conditions for the C­(sp^2^)–H/C­(sp^3^)–H difunctionalization of oxime ethers, the scope of the transformation was interrogated (Table 2). Difunctionalization of oxime derivatives possessing electron-donating groups reacted efficiently (**2b**-**2c, 2f**); however, low yields were obtained with electron-withdrawing substituents (**2e** and **2g**) due to the low-yielding C­(sp^3^)–H arylation step. To transcend conventional acetoxylation and arylation of PIDA, we illustrated that replacing HFIP with MeOH for the first step of the reaction led to the C­(sp^2^)–H methoxylation/C­(sp^3^)–H arylation difunctionalization product (**2h**) via methanolysis of PIDA. Notably, this highlights that in situ derivatization of commercially available PIDA to other useful HVIs can potentially avoid the need for independent synthesis of these tandem reagents (*vide infra*). Oximes ethers possessing fused aromatic rings, **1i** and **1j**, underwent efficient C­(sp^2^)–H acetoxylation/C­(sp^3^)–H arylation difunctionalization, leading to **2i** and **2j**, respectively. Installation of the methyl group at the ortho position of the oxime ether (**1k**) completely shut down the reaction (**2k**), as the photoisomerization step failed due to the steric effects. Subjecting aryl oxime ether with branched alkyl substituents to the reaction conditions proceeded smoothly (**2l**-**2m**). Cyclic oxime **1n** furnished the C­(sp^2^)–H acetoxylation/C­(sp^3^)–H arylation **2n** in 44% yield. Heterocyclic oxime ether possessing a benzothiophene unit (**1o**) reacted modestly under our protocol, resulting in **2o** in low yield, featuring preferential functionalization at the C-4 position over C-2.

**Table 2:**
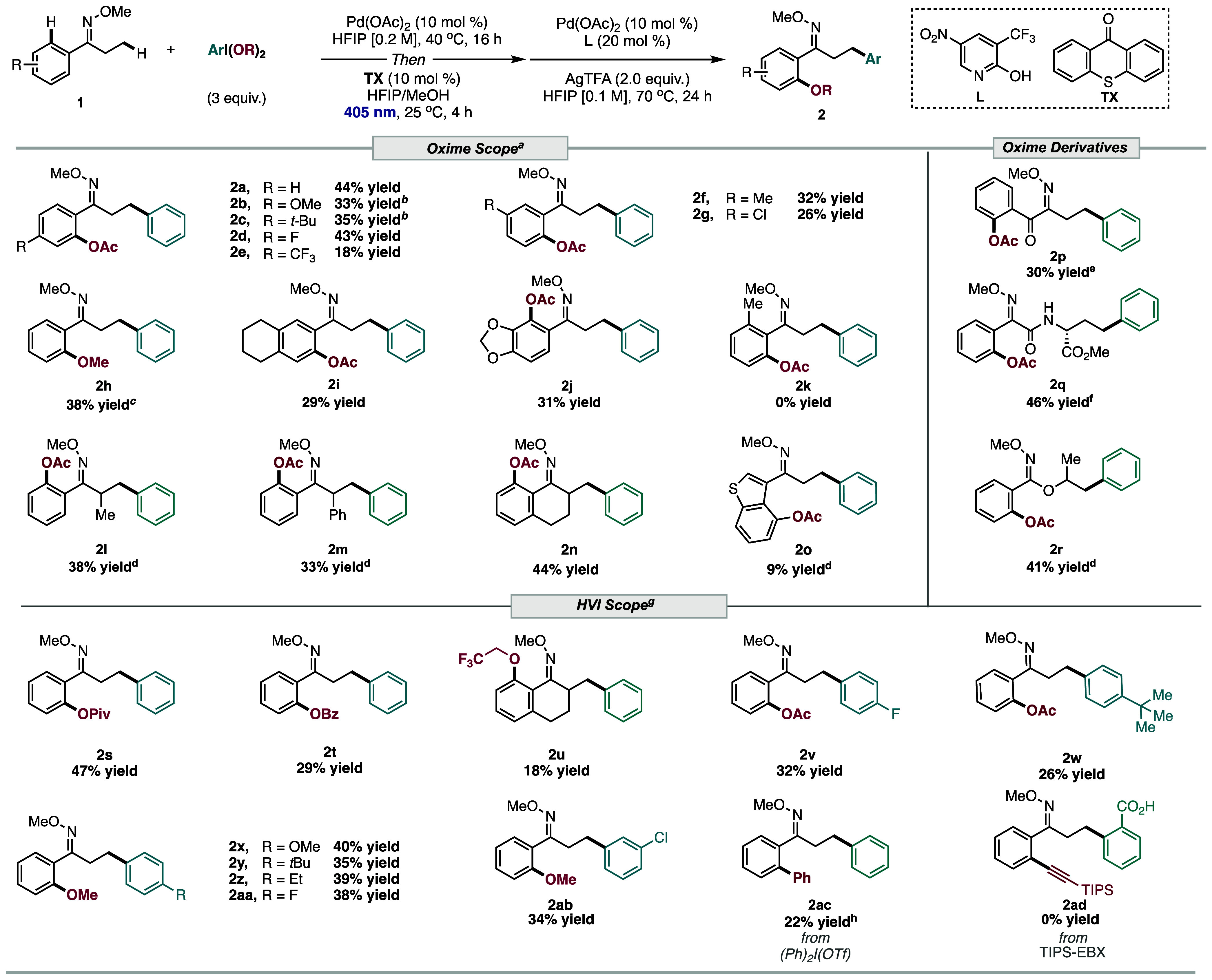
Reaction Scope^a^

a Condition A: First step = **1** (1 equiv), PhI­(OAc)_2_ (3 equiv), Pd­(OAc)_2_ (10 mol %), HFIP [0.20 M], 40 °C; Second step = TX (10 mol %), HFIP/MeOH, 405 nm, 25 °C, 4 h; Third step = Pd­(OAc)_2_ (10 mol %), **L** (20 mol %), AgTFA (2.0 equiv), HFIP [0.1 M], 70 °C, 24 h.

b PhI­(OAc)_2_ (1.5 equiv).

c MeOH used instead of HFIP for the first step.

d ^1^H NMR yield.

e Reaction temperature = 60 °C for the first step, and reaction time = 36 h for the first step, and 48 h for the third step.

f Third step condition = Pd­(OAc)_2_ (10 mol %), AgOAc (1 equiv), *t*BuCO_2_H (1.0 equiv), HFIP (0.10 M), 90 °C, and 48 h.

g Using ArI­(OR)_2_ instead of PhI­(OAc)_2_.

h Pd­(OAc)_2_ (10 mol %), TX (10 mol %), Ph_2_IOTf (2.0 equiv), dry MeOH [0.10 M], N_2_, 390 nm Kessil lamp, 25 °C, 24 h; then, Pd­(OAc)_2_ (10 mol %), **L** (20 mol %), AgTFA (2.0 equiv), HFIP [0.10 M], 70 °C, 24 h.

Next, related photochromic DGs were tested under this successive C–H acetoxylation/arylation difunctionalization protocol. Oxo-oxime **1p** resulted in the difunctionalized product **2p** in 30% yield. Interestingly, exposure of amino acid-derived oxime ether **1q** to the reaction resulted in the desired difunctionalization product **2q**, with the arylation event occurring at the γ-position of the alkyl substituent (46% yield). This outcome is likely due to the bidentate coordination ability of **1q** for the C–H arylation step. Oxime-acetimidate**1r** was examined under the reaction conditions and led to the corresponding difunctionalized product **2r** in good yield. These results illustrate that analogous photochromic DGs could be employed under this paradigm.

Other HVIs and related tandem electrophiles were tested under our protocol. C­(sp^2^)–H pivaloxylation and O-benzoylation, followed by successive C­(sp^3^)–H arylation of **1s** and **1t**, proceed efficiently to yield **2s** and **2t** with phenyliodine­(III) dipivalate and -benzyloxy, respectively. Installation of trifluoroethanol via C­(sp^2^)–H etherification and C­(sp^3^)–H arylation via in situ formation of phenyliodine­(III) ditrifluoroethoxy, leading to **2u** transpired effectively. Next, the aromatic ring scope of the HVIs was then investigated. HVIs possessing electron-donating groups (**2w**-**2z**) and inductive groups (**2v**, **2aa**-**2ab**) were the most effective for the C­(sp^2^)–H oxygenation/(C­(sp^3^)–H arylation difunctionalization of the oxime substrates. Notably, employment of diphenyl iodonium salt resulted in the formation of diarylated oxime product **2ac** (Table S6). However, difunctionalization via C­(sp^2^)–H alkynylation/C­(sp^3^)–H arylation with TIPS-ethynylbenziodoxolone (TIPS-EBX) failed under the reaction conditions (**2ad**).

The proposed mechanism of the transformation is shown in Scheme 2. Based on the *E*-geometry of the oxime (***E***)**-1**, the preference of the DG allows the nitrogen lone pair of the oxime to directly activate the β-C­(sp^2^)–H bond via Pd-catalyzed concerted metalation deprotonation (CMD) to generate a palladacycle **3** that undergoes oxidative addition with the HVI electrophile to give Pd (IV) complex **4**. Reductive elimination of the latter generates the β-C­(sp^2^)–H oxygenation product **5** and the aryl iodide (Ar–I) byproduct. Then, the reaction is irradiated in the presence of a thioxanthone to empower the photoisomerization of (***E***)-**5** to give (***Z***)-**6**. The oxime-ether photoisomerization step via an energy transfer pathway was confirmed using UV–vis and PhotoNMR studies (Figures S1–S2). Following the addition of new Pd-catalyzed conditions, (***Z***)-**6** undergoes a directed CMD at the β’-C­(sp^3^)–H bond based on the new directionality of the DG to furnish Pd (II) complex **7**. Next, the latter undergoes oxidative addition with aryl iodide, recycled from the first step, to give Pd (IV) complex **8**. Subsequent reductive elimination of **8** yields the difunctionalized product **(** *Z* **)-2**.

**Scheme 2:**
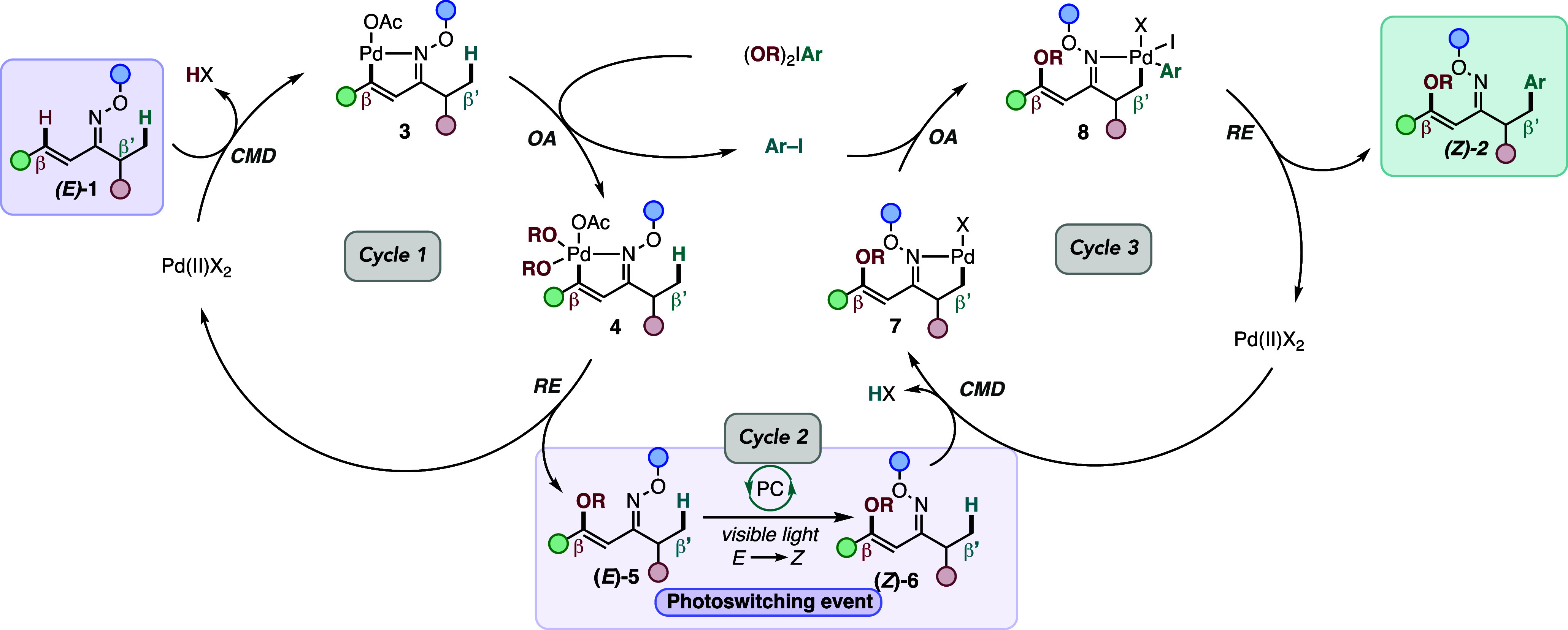
Proposed Mechanism

Finally, the synthetic utility of the transformation was investigated (Scheme 3). We aimed to reverse the C–H difunctionalization sequence with the *Z*-oxime isomer (Scheme 3A). However, photoswitching of the Z-oxime can be challenging since the system is deconjugated, resulting in a high triplet energy.^15^ Thus, the reverse C–H difunctionalization was performed under thermal conditions. Subjecting **(** *Z* **)-1p** to the C­(sp^3^)–H arylation conditions at 70 °C and then heating to 110 °C for an additional 6 h resulted in the trans arylation product **(** *E* **)-9**. Notably, the C­(sp^3^)–H arylation and isomerization occurred successively in a single pot. With the appropriate geometry of (***E***)**-9**, the subsequent C­(sp^2^)–H acetoxylation occurred productively, leading to **10** in 44% overall yield. The formed oxime products were further derivatized (Scheme 3B). Beckmann-type rearrangement of **2h** furnished aniline **11** in 91% yield.^20^ Photoinduced, nitroarene-promoted anaerobic cleavage of **2h** to ketone **12** occurred in modest yield.^21^ Lastly, our concept was demonstrated in the synthesis of antiarrhythmic propafenone (Scheme 3C).^22^ Oxime condensation of **13**, followed by our C–H difunctionalization strategy via C­(sp^2^)–H acetoxylation and C­(sp^3^)–H arylation, and subsequent global hydrolysis, led to **14**. Next, etherification of **14** with 2-(chloromethyl)­oxirane, followed by nucleophilic ring-opening of the installed epoxide with propyl amine, furnished propafenone (**15**) in 7.6% yield over 7 linear steps.

**Scheme 3:**
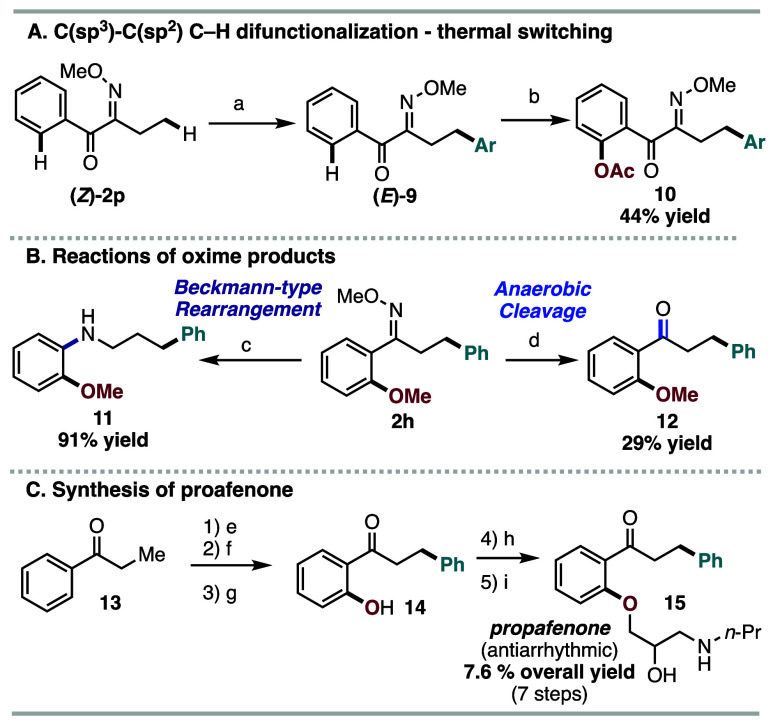
Synthetic Utility^a^

a Conditions: (a) Pd­(OAc)_2_ (10 mol %), 4–F-C_6_H_4_I (2 equiv), **L** (20 mol %), AgTFA (2.0 equiv), HFIP [0.1 M], 70 °C, 48 h, then 110 °C, 6 h. (b)­Pd­(OAc)_2_ (10 mol %), PhI­(OAc)_2_ (2 equiv), HFIP [0.2 M], 60 °C, 16 h. (c) DIBAL-H (4 equiv), Toluene, −10 °C, 2 h.(d) 4-Nitrophthalonitrile (3 equiv), CH_3_CN:H_2_O, 390 nm, 23 °C, 48 h. (e) O-methylhydroxylamine hydrochloride (1.5 equiv), sodium acetate (2 equiv), EtOH:H_2_O (reflux), 16 h, 85% yield of 11:1 *E:Z*-oxime ether. (f) Condition A, Table 1, 43% yield. (g) 37% HCl, Et_2_O, 23 °C, 12 h, 93% yield. (h) 2-(chloromethyl)­oxirane, NaOH, 110 °C, 18 h, 49% yield. (i) Propyl amine, CH_3_OH, 6 h, 50% yield.

In conclusion, we have illustrated the implementation of oxime ethers as effective photochromic DGs for spatially controlled C–H difunctionalization of distinct C–H bonds. The benefits of our protocol involve photocontrolled functionalization using simple Pd-catalyzed conditions with HVI as tandem electrophiles and the employment of an organocatalytic photosensitizer. All three transformative steps of the reaction, C­(sp^2^)–H functionalization, photoisomerization, and C­(sp^3^)–H functionalization, occur in a fully controlled manner in a semi-two-pot fashion. Also, the reverse functionalization events can be achieved under thermal control. We anticipate that this work on the merger of photochromism and directed transition metal-catalyzed C–H functionalization will provide the synthetic community with a powerful platform for the controlled multifunctionalization of organic molecules.

## Supplementary Material



## Notes

Experimental details, optimization studies, characterization data, and NMR spectra (PDF)

## ABBREVIATIONS

PC: photocatalyst
Pd: palladium
HVI: hypervalent iodine
TX: thioxanthone
PIDA: phenyliodine diacetate.

## References

[1] Lucas E. L., Lam N. Y. S., Zhuang Z., Chan H. S. S., Strassfeld D. A., Yu J.-Q. (2022). Palladium-Catalyzed
Enantioselective β-C­(sp^3^)–H Activation Reactions
of Aliphatic Acids: A Retrosynthetic Surrogate for Enolate Alkylation
and Conjugate Addition. Acc. Chem. Res..

[2] Gutekunst W. R., Baran P. S. (2011). C–H functionalization logic in total synthesis. Chem. Soc. Rev..

[3] Sun H., Huang Y. (2015). Recent Progress in
the Development
of Multitasking Directing Groups for Carbon–Hydrogen Activation
Reactions. Synlett.

[4] Daugulis O., Roane J., Tran L. D. (2015). Bidentate, Monoanionic
Auxiliary-Directed Functionalization of Carbon–Hydrogen Bonds. Acc. Chem. Res..

[5] Engle K. M., Mei T.-S., Wasa M., Yu J.-Q. (2012). Weak Coordination
as a Powerful Means for Developing Broadly Useful C–H Functionalization
Reactions. Acc. Chem. Res..

[6] Parasram M., Gevorgyan V. (2017). Silicon-Tethered
Strategies for C–H
Functionalization Reactions. Acc. Chem. Res..

[7] Gandeepan P., Ackermann L. (2018). Transient
Directing Groups for Transformative
C–H Activation by Synergistic Metal Catalysis. Chem..

[8] Lam N. Y. S., Fan Z., Wu K., Park H. S., Shim S. Y., Strassfeld D. A., Yu J.-Q. (2022). Empirical Guidelines
for the Development of Remote Directing Templates through Quantitative
and Experimental Analyses. J. Am. Chem. Soc..

[9] a Topics in Current Chemistry: C–H Activation; Yu, J.-Q. , Shi, Z. , Eds.; Springer, Berlin, 2010, Vol. 292. 21500400

[10] Nadres E. T., Santos G. I. F., Shabashov D., Daugulis O. (2013). Scope and Limitations
of Auxiliary-Assisted, Palladium-Catalyzed Arylation and Alkylation
of Sp^2^ and Sp^3^ C-H Bonds. J. Org. Chem..

[11] Shabashov D., Daugulis O. (2010). Auxiliary-Assisted
Palladium-Catalyzed Arylation and Alkylation of Sp^2^ and
Sp^3^ Carbon-Hydrogen Bonds. J. Am.
Chem. Soc..

[12] a Irie, M. ; Yokoyama, Y. ; Seki, T. New Frontiers in Photochromism; Springer: Tokyo, 2013.

[13] Dugave C., Demange L. (2003). Cis–Trans Isomerization
of Organic Molecules and Biomolecules: Implications and Applications. Chem. Rev..

[14] Constable A.
G., McDonald W. S., Sawkins L. C., Shaw B. L. (1980). Attempts to Cyclopalladate Some Aliphatic
Oximes, NN-dimethylhydrazones,
Ketazines, and Oxime O-allyl ethers. J. Chem.
Soc., Dalton Trans..

[15] Zhang X., Rovis T. (2021). Photocatalyzed Triplet
Sensitization of Oximes Using Visible Light
Provides a Route to Nonclassical Beckmann Rearrangement Products. J. Am. Chem. Soc..

[16] Hirata Y., Kimura S., Higashida K., Yoshino T., Matsunaga S. (2025). Site-Selective
C­(sp^3^)–H and Switchable C­(sp^3^)–H/C­(sp^2^)–H Functionalization Enabled by Electron-Deficient
Cp*CF_3_Ir­(III) Catalyst and Photosensitizer. Angew. Chem., Int. Ed..

[17] Pan L., Wang L., Chen Q., He M. (2016). Palladium-Catalyzed Oxime Ether Directed Regioselective C-H Alkoxylation
of Arenes. Synth. Commun..

[18] Strieth-Kalthoff F., James M. J., Teders M., Pitzer L., Glorius F. (2018). Energy Transfer Catalysis Mediated
by Visible Light: Principles, Applications, Directions. Chem. Soc. Rev..

[19] Wang P., Farmer M. E., Huo X., Jain P., Shen P.-X., Ishoey M., Bradner J. E., Wisniewski S. R., Eastgate M. D., Yu J.-Q. (2016). Ligand-Promoted
Meta-C–H Arylation of Anilines, Phenols, and Heterocycles. J. Am. Chem. Soc..

[20] Kaur K., Srivastava S. (2020). Beckmann Rearrangement
Catalysis: A Review of Recent
Advances. New J. Chem..

[21] Göttemann L. T., Wiesler S., Sarpong R. (2023). Oxidative
Cleavage of Ketoximes to Ketones using Photoexcited Nitroarenes. Chem. Sci..

[22] Somberg J.
C., Tepper D., Landau S. (1988). Propafenone:
A new antiarrhythmic agent. Am. Heart J..

