# Supplementary material for: Photochromic Oxime Directing Groups for Spatially Controlled Pd-Catalyzed C–H Difunctionalization with Tandem Electrophiles
Source: ACS Catal. 2025 Sep 23;15(19):16917–23. doi: 10.1021/acscatal.5c05469 (PMC12501948; doi:10.1021/acscatal.5c05469)
Supplement: Supplementary file 1 [file cs5c05469_si_002.pdf]

Photochromic Oxime Directing Groups for Spatially Controlled Pd-Catalyzed C–H Difunctionalization with Tandem Electrophiles

Mahmoud R. Saleh,[‡] Mahmoud Afrasi,[‡] Ajay H. Bansode, Poulami Ghosh, Dan E. Wise, and Marvin Parasram*

Department of Chemistry, New York University, 24 Waverly Place, 3rd floor, New York, NY 10003
*parasram@nyu.edu

Supporting Information

Table of Contents

General Information 2
General Procedures 3
Optimization of Reaction Conditions 11
Mechanistic studies 14
Characterization Data of Starting Materials 16
Characterization Data of Difunctionalized Products 19
Characterization Data of Difunctionalized Products (^{1}H NMR Yield) 29
Spectral Data: ^{1}H and ^{13}C NMR Spectra 31
References: 79

General Information

All requisite chemicals were purchased from Fisher Scientific, Sigma Aldrich (Merck), Oakwood Chemical (Oakwood Products), Ambeed, TCI, and used without further purification unless otherwise stated. All NMR spectra (^{1}H, ^{13}C, and ^{19}F) were recorded on Bruker 400 MHz; 500 MHz Avance spectrometers and are referenced to the deuterated solvent resonance. Chemical shifts (δ) are reported in parts per million (ppm), multiplicity (s = singlet, br = broad, d= doublet, t = triplet, q = quartet, qt = quintet, sext = sextet, m = multiplet), and coupling constants (J) are in Hertz (Hz). All reactions were carried out under ambient conditions unless otherwise noted. Thin-layer chromatography (TLC) was performed on 250-μm glass-backed silica gel plates and column chromatography were performed using 200–300 mesh silica gel unless otherwise stated. Deuterated chloroform ($CDCl_3$) was purchased from Cambridge Isotopes. GC chromatograms were taken on an Agilent 8890 GC with 5977B MSD, and helium as the carrier gas. High-resolution mass spectra (HRMS) were obtained on an Agilent 6224 TOF LC/MS which was acquired through the support of New York University. UV-Visible spectra were recorded on an Agilent Cary 3500 UV-Visible spectrophotometer. We utilized 34 W Kessil Lamps with varying wavelengths as well as 18 W EvoluChem 405 nm LEDs for the photochemical setups.

General Procedures

General Procedure A. Conditions for the sequential Pd-catalyzed C(*sp*²)–H oxygenation and C(*sp*³)–H arylation difunctionalization of oxime ethers using a hypervalent iodine reagent as a tandem electrophile

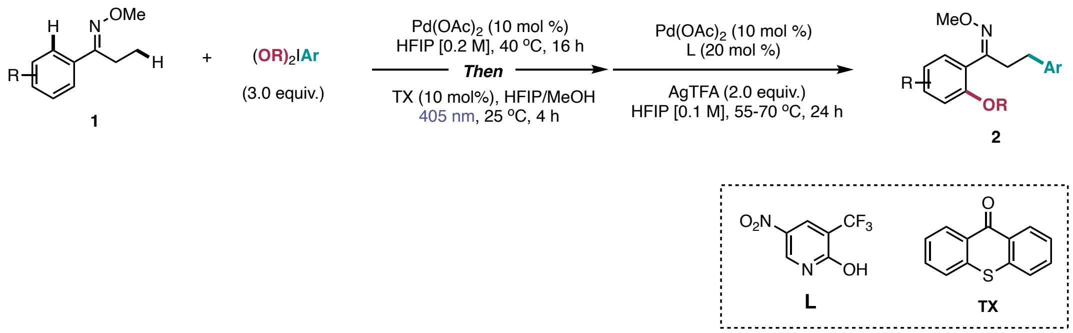

For C(sp²)–H acetoxylation and C(sp³)–H arylation: A 2-dram vial equipped with a magnetic stirrer bar was charged with oxime ether **1** (0.20 mmol, 1 equiv.), Pd $(OAc)_2$ (4.5 mg, 0.02 mmol, 10 mol %), **$(OR)_2IAr$** (0.60 mmol, 3.0 equiv.), and HFIP (1.0 mL, 0.2 M). The vial was sealed with a cap, placed in a preheated heating block at 40 °C, and then continuously stirred for 16 h. Upon complete consumption of the starting material (monitored by TLC or GCMS), the reaction was cooled to room temperature, and thioxanthone photocatalyst (4.3 mg, 0.02 mmol, 10 mol %) in 1.0 mL methanol was added. The mixture was irradiated with a 405 nm light source for 4 hours (with a fan set up to maintain room temperature). After photoirradiation, the reaction mixture was passed through a short plug of Celite to remove Pd black. The filtrate was concentrated under reduced pressure, and the residue was redissolved in HFIP (2.0 mL). $Pd(OAc)_2$ (4.5 mg, 0.02 mmol, 10 mol %), (2,2,2-trifluoroacetoxy)silver (88 mg, 0.40 mmol, 2 equiv.), and 3-nitro-5-(trifluoromethyl)pyridin-2-ol (8.3 mg, 0.04 mmol, 20 mol %) were added. The vial was sealed with a pressure cap, placed in a heating block preheated to 70 °C, and stirred for 24 h. Reaction progress was monitored by GCMS. Upon completion, the mixture was filtered through Celite, and the crude residue was purified by flash column chromatography on silica gel to afford the desired product.

For C(sp²)–H methoxylation and C(sp³)–H arylation: The procedure above was followed, except methanol was used instead of HFIP in the first step.

For C(sp²)–H etherification and C(sp³)–H arylation: The procedure above was followed, except trifluoroethanol (TFE) was used instead of HFIP in the first step.

General Procedure B. Conditions for sequential C(*sp²*)–H and C(*sp³*)–H di-arylation functionalization using diphenyliodonium triflate (Ph_2IOTf)

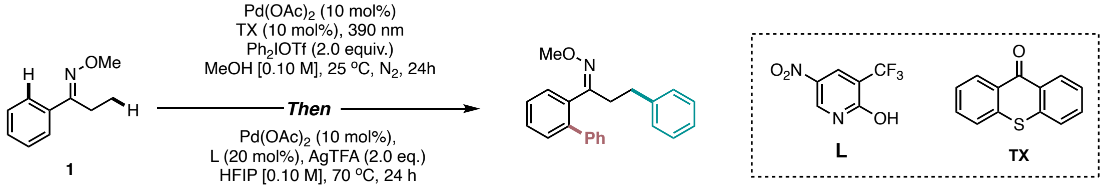

In an oven dried 2-dram vial, oxime ether **1a** (0.10 mmol, 1.0 equiv.), $Pd(OAc)_2$ (2.3 mg, 0.01 mmol, 10 mol %), thioxanthone (2.2 mg, 0.01 mmol, 10 mol %), and diphenyliodonium triflate (Ph_2IOTf, 86 mg, 0.20 mmol, 2.0 equiv.) were dissolved in dry methanol (1.0 mL, 0.1 M). The vial was sealed with a septum cap, and the whole mixture was degassed with N_2 for 15 minutes and irradiated at 390 nm (Kessil lamp) for 24 hours (with a fan set up to maintain room temperature). After completion, as indicated by GCMS or TLC, the reaction mixture was filtered through a plug of celite, and methanol was removed. The crude residue was redissolved in HFIP (1.0 mL, 0.1 M), followed by the addition of $Pd(OAc)_2$ (2.3 mg, 0.01 mmol, 10 mol %), (2,2,2-trifluoroacetoxy)silver (44 mg, 0.40 mmol, 2.0 equiv.), and 3-nitro-5-(trifluoromethyl)pyridin-2-ol (4.2 mg, 0.04 mmol, 20 mol %). The vial was sealed with a pressure cap and placed in a block pre-heated to 70 °C. Upon completion, the mixture was filtered through Celite, and the crude residue was purified by flash column chromatography on silica gel to afford the desired product.

General Procedure C. Conditions for the directed C(sp²)-H/C(sp³)-H Difunctionalization using oxooxime as DG

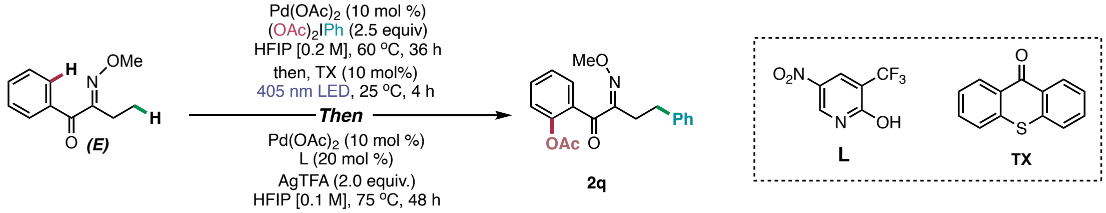

In a 1-dram vial equipped with a stir-bar, oxooxime (0.2 mmol), $Pd(OAc)_2$ (10 mol %), HVI (2.5 equiv.), and HFIP were charged. The vial was then heated at 60 °C for 36 h. Then, TX (10 mol %)

and 1.0 mL of methanol were added. The vial was then irradiated with 405 nm light for 4 h. After the reaction was completed, a plug of Celite was used to remove Pd black, and the solvent was removed. Then, $Pd(OAc)_2$ (10 mol %), AgTFA (2 equiv.), ligand (20 mol %), and HFIP (0.1 M) were added, and the reaction vessel was left at 75 °C to stir at 600 rpm for 48 h. Completion of the reaction was determined by TLC and GC-MS analysis. After the reaction was complete, the solvent was removed. The crude product was purified by column chromatography (Hex:DCM:Et_2O = 7:2:1/5:3:2) to afford the difunctionalized product.

General Procedure D. Conditions for the directed C(sp^2)-H/C(sp^3)-H Difnctionalization using amidoxime as DG. [1]

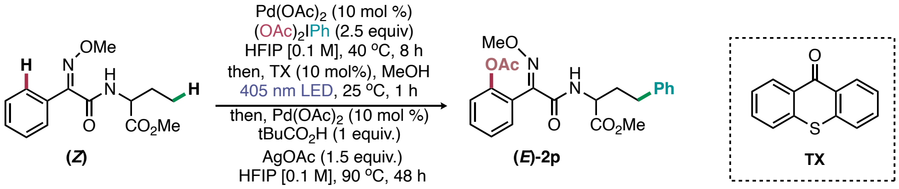

In a 1-dram vial equipped with a stir-bar, amidoxime (0.2 mmol), Pd(Oac)₂ (10 mol %), HVI (2.5 equiv.), and HFIP were charged. The vial was then heated at 40 °C for 8 h. Then, TX (10 mol %) and 1.0 mL of methanol were added. The vial was then irradiated with 405 nm light for 1 h. After the reaction was completed, a plug of Celite was used to remove Pd black, and the solvent was removed. Then, $Pd(Oac)_2$ (10 mol %), AgOAc (1 equiv.), *t*$BuCO_2H$ (1.0 equiv.), and HFIP (0.1 M) were added, and the reaction vessel was left at 90 °C to stir at 600 rpm for 48 h. Completion of the reaction was determined by TLC and GC-MS analysis. After the reaction was complete, the solvent was removed. The crude product was purified by column chromatography (Hex: EtOAc = 9:1) to afford the difunctionalized product.

General Procedure E. Conditions for synthesis of oxime ethers substrates

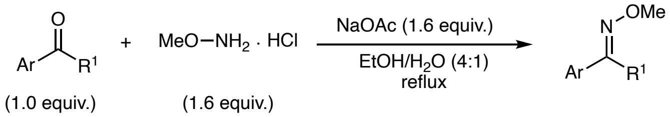

Following the reported procedure[8], in a 50 mL round-bottom flask equipped with a condenser, Ketone (1.0 equiv.) was dissolved in the mixture of $EtOH/H_2O$ (4:1, 0.30 M). Then, O-methylhydroxylamine hydrochloride (1.6 equiv.) and sodium acetate (2.0 equiv.) were added in

one portion. The reaction mixture was stirred and refluxed until the consumption of the starting material was observed by TLC. After that, the reaction was cooled to room temperature, diluted with water, extracted with ethyl acetate three times. The combined organic phase was then washed with brine and dried over anhydrous Na_2SO_4 and concentrated. The residue was purified by column chromatography on silica gel.

General Procedure F. Synthesis of Conjugated Oxooxime [3]

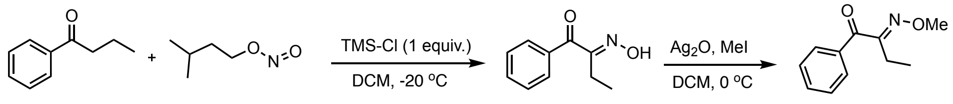

To a solution of 10 mmol ketone in 5 mL DCM was added 1 eq. TMSCl (trimethylchlorosilane, 1.24 mL) at -20 °C. To this cooled solution, 1 eq. isoamyl nitrite (1.34 mL) was added dropwise. The reaction was found to be instantaneous, but the mixture was stirred at r.t. for an additional period of 1 h before working up. The solution was directly concentrated in vacuo. The crude product was purified by flash column chromatography on silica gel (eluting with hexane/ethyl acetate = 20:1) and the corresponding oximes was obtained. Then, Ag_2O (3.2 g, 13.8 mmol) was slowly added with stirring to a solution of (E)-2-(hydroxyimino)-1-phenylbutan-1-one (12.3 mmol) and MeI (8.7 mL, 61.5 mmol) in 15 mL of CH_2Cl_2, and cooled with an ice–water bath. After 0.5 h of reaction, the precipitate was filtered off and washed with CH_2Cl_2. The CH_2Cl_2 was evaporated from the filtrate, yielding the corresponding Oxooxime in 35 % yield.

General Procedure G. Synthesis of oxime derivative (**1r**) [4,5]

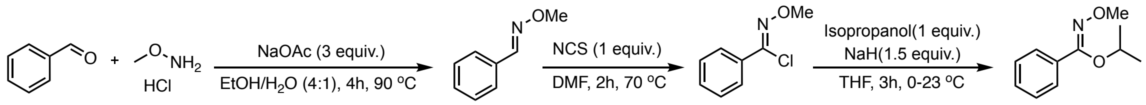

In a 50 mL round-bottom flask equipped with a condenser, benzaldehyde (1.0 equiv.) was dissolved in the mixture of EtOH/H_2O (4:1, 0.30 M). Then, O-methylhydroxylamine hydrochloride (1.6 equiv.) and sodium acetate (3.0 equiv.) were added in one portion. The reaction mixture was stirred and refluxed until the consumption of the starting material was observed by TLC. Then, the reaction was cooled to room temperature, diluted with water, and extracted with ethyl acetate three times. The organic layer was washed with saturated $NaHCO_3$ (aq), dried over anhydrous Na_2SO_4, filtered, and concentrated in vacuo to give oxime, which was used in the next step without further purification.

A 100 mL flask was charged with oxime (30 mmol), and anhydrous DMF (20 mL). N-Chlorosuccinamide (30 mmol, 1.0 equiv.) was added in portions, and the reaction mixture was heated at 70 °C for 2 hours. After 2 hours, the system was cooled to rt. Upon completion, the reaction mixture was poured into ice water (100 mL), and the resulting mixture was extracted with ethyl acetate (3 x 100 mL). The combined organic layers were washed with saturated $NaHCO_3$ (aq), brine, dried over Na_2SO_4, filtered, and concentrated in vacuo. The crude residue was purified by flash chromatography to afford the corresponding substrate.

Next, to a flame-dried round bottom flask with a stir bar was added NaH (60% dispersion in mineral oil) (1.5 mmol, 1.5 equiv.). The flask was evacuated and refilled with N_2 three times. Dry THF (5 mL) was added via syringe. The reaction was cooled to 0 °C and then alcohol (1 mmol, 1 equiv.) was added. The reaction mixture was allowed to stir for 3 h at room temperature. Then, the reaction was cooled to 0 °C and the (Z)-N-methoxybenzimidoyl chloride (1.1 mmol, 1.1 equiv.) in THF (5 mL) was added. The reaction was stirred at room temperature until full consumption of alcohol. Upon completion, the reaction was poured into a separatory funnel containing Et_2O and H_2O. The aqueous phase was extracted with Et_2O. The combined organic phases were dried over Na_2SO_4, filtered, and concentrated. The crude reaction mixture was loaded onto silica gel and purified to afford the oxime derivative.

Procedure H

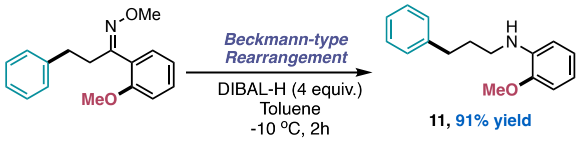

To a solution of (*Z*)-1-(2-methoxyphenyl)-3-phenylpropan-1-one *O*-methyl oxime (**2h)** (100 mg, 0.371 mmol) in dry toluene (10 mL) was added dropwise a solution of DIBALH (1.0 M in toluene, 1.49 mL, 1.49 mmol) at −10 °C, and the reaction mixture was stirred for 2 hours. After that, 10% aq. HCl was added slowly until a precipitate was formed, followed by 10% aq. NaOH until the solution was alkaline. The mixture was extracted with ether (2 x 10 mL) and CH_2Cl_2 (2 x 10 mL). The combined organic layers were dried with Na_2SO_4, filtered, and the solvents removed in vacuo. The residue was purified by flash chromatography on silica gel using EtOAc/*n*-hexane (1/5 (v/v)) to afford the corresponding 2-methoxy-N-(3-phenylpropyl)aniline (**11**) in 82 mg (91% yield).

Procedure I

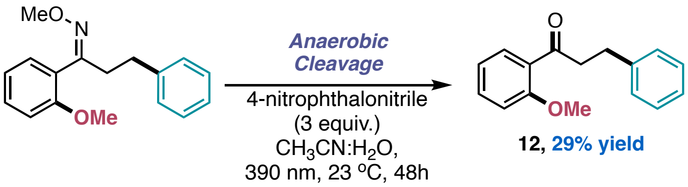

A flame-dried 1-dram clear glass vial was charged with a magnetic stir bar, (*Z*)-1-(2-methoxyphenyl)-3-phenylpropan-1-one *O*-methyl oxime (26.9 mg 0.10 mmol) and 4-nitrophtalonitrile (51.9 mg, 0.30 mmol). The vial was sealed using a septum cap, and the mixture was evacuated and backfilled with nitrogen (3x) before the addition of anhydrous acetonitrile (0.4 mL) and distilled water (0.1 mL). The resulting solution was sparged with nitrogen for a period of 5 min, after which the septum cap was exchanged for a Teflon cap, which was reinforced by sealing with black tape. The sealed vial was placed on a magnetic stir plate in between **two Kessil PR160L lamps (390 nm)** with each at a distance of 3 cm. A stream of air/nitrogen was pointed at the vial using a funnel that ensured cooling of the reaction mixture, and both lamps were switched on. Stirring under irradiation was continued for 48 h before the irradiation and cooling were discontinued. The reaction mixture was then diluted with DCM (2 mL) as well as distilled water (2 mL), and the phases were separated. The aqueous phase was extracted with DCM (3 x 2 mL), and the combined organic layers were dried over anhydrous sodium sulfate and carefully concentrated under reduced pressure. The crude product was then subjected to preparative thin-layer chromatography/flash column chromatography to obtain the 1-(2-methoxyphenyl)-3-phenylpropan-1-one (**12**) in 7.0 mg (29% yield).

Procedure J

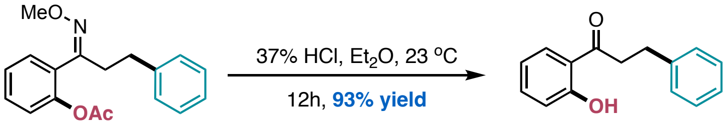

(Z)-2-(1-(methoxyimino)-3-phenylpropyl)phenyl acetate **2a** (10.0 mg, 33.6 mmol) was dissolved in Et_2O (2 mL) and stirred for 10 min, then 37% HCl (1 mL) was added, and then the reaction mixture was stirred at 25 °C for 12 h. After the reaction, the reaction mixture was diluted with ethyl acetate and washed with water (2 x 5 mL), Na_2CO_3 (2 x 5 mL), and brine (2 x 5 mL). The organic layer was dried over $MgSO_4$, filtered, and concentrated. The resulting oil was purified by chromatography on silica gel to afford the 1-(2-hydroxyphenyl)-3-phenylpropan-1-one in 7.1 mg (93% yield).

Procedure k

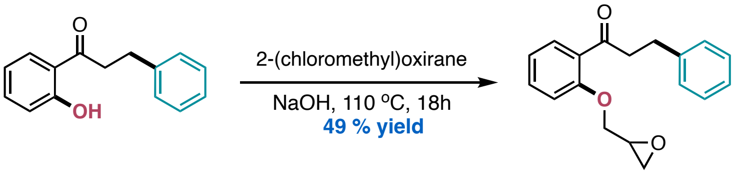

A powdered sodium hydroxide (42.3 mg, 0.88 mmol) was added to a solution of 1-(2-hydroxyphenyl)-3-phenylpropan-1-one (200 mg, 0.88 mmol) in excess of rac-epichlorohydrin (2.45 g, 26.5 mmol). The reaction mixture was stirred under reflux for 18 h. After this period, the reaction mixture was concentrated under reduced pressure, obtaining a yellow oil. The oil was dissolved in 5.0 mL of ethyl ether and washed 3 times with water (5.0 mL). Then, the organic phase was dried over anhydrous sodium sulfate, and after filtration, the solvent was evaporated under reduced pressure, obtaining the crude product as a white solid. In the sequence, the crude product was purified by flash chromatographic column with flash silica gel using hexane: EtOAc (90: 10) as eluent, to give a white solid identified as 1-{o-[(2-oxiranyl)methoxy]phenyl}-3-phenyl-1-propanone (120.0 mg, 49% yield).

Procedure L

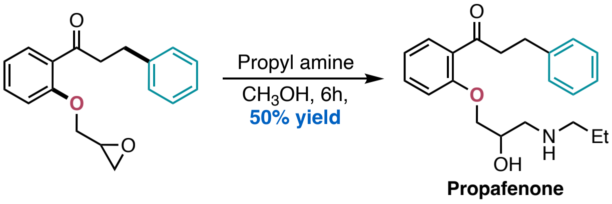

An epoxide 1-{o-[(2-oxiranyl)methoxy]phenyl}-3-phenyl-1-propanone (**7**) (20.0 mg, 0.07 mmol) and the propan-1-amine (5.0 mg, 0.08 mmol) were dissolved in CH_3OH and held at reflux for 6 h. The mixture was evaporated to dryness, and the oily residue was purified by column chromatography (silica gel, CH_2Cl_2/CH_3OH/conc NH_4OH, 200:10:1) to afford 12.5 mg (50% yield) of Propafenone.

Procedure M: Synthesis of oxime derivative (**1q**)

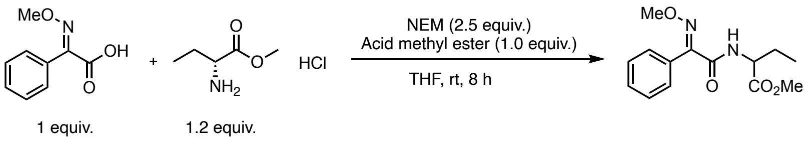

To a stirred solution of 2-methoxyiminoacetic acid (700 mg, 3.91 mmol, 1.0 equiv.) in dry tetrahydrofuran (THF, 20 mL, 0.2 M) under nitrogen at 0 °C was added N-ethylmorpholine (NEM, 1.27 mL, 9.78 mmol, 2.5 equiv.). After 5 minutes, isobutylchloroformate (0.51 mL, 3.91 mmol, 1.0 equiv.) was added dropwise, and the mixture was stirred at 0 °C for 1 hour. The Methyl (R)-2-aminobutanoate hydrochloride (4.69 mmol, 1.2 equiv.) was then added in one portion, and the reaction mixture was warmed to room temperature and stirred overnight. The reaction mixture was concentrated under reduced pressure, and the residue was diluted with water (20 mL) and extracted with ethyl acetate (3 × 25 mL). The combined organic layers were washed with brine (30 mL), dried over anhydrous sodium sulfate (Na_2SO_4), filtered, and concentrated in vacuo. The crude residue was purified by silica gel flash chromatography (eluent: hexanes/ethyl acetate, 10-20 %) to afford the desired Amido-oxime product as a yellow oil (436 mg, 40% yield).

Procedure N: Conditions for sequential C(*sp*^{3})- C(*sp*^{2}) difunctionalization using thermal method

In a 1-dram vial equipped with a magnetic stir bar, oxooxime (Z)-**1p** (0.10 mmol), $Pd(OAc)_2$ (10 mol %), AgTFA (2.0 equiv.), and ligand (20 mol %) were combined in HFIP (0.1 M). The reaction mixture was stirred at 75 °C for 48 h at 600 rpm. After completion of the initial C(sp^3)–H arylation, the vial was heated to 110 °C for an additional 6 h to induce Z/E isomerization, furnishing the trans-arylated intermediate (E)-9. Without purification, $Pd(OAc)_2$ (10 mol %) and HVI (2.5 equiv.) were then added to the same vial, and the mixture was heated at 60 °C for 36 h to effect C(sp^2)–H acetoxylation. Upon completion, the reaction mixture was cooled, and the solvent was removed under reduced pressure. The crude residue was purified by column chromatography (Hexanes:DCM:Et_2O = 7:2:1 to 5:3:2) to afford the difunctionalized product **10** in 44% yield.

Optimization of Reaction Conditions

Table S1: Evaluation of photosensitizers with different triplet energies.

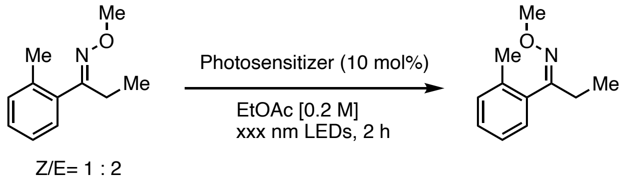

Entry	Photosensitizer	Light source	E_T (Kcal/mol)	Z/E[a]
1	none	390 nm	–	1 : 2
2[b]	$Ir[dF(CF_3)ppy]_2(dtbbpy)PF_6$	427 nm	61.8	99 : 1
3	Benzil	427 nm	54.0	1 : 1
4	4CzIPN	427 nm	53.0	2 : 1
5	Thioxanthone	390 nm	65.4	99 : 1

[a]Z/E ratios were determined by GCMS analysis.
[b]1.0 mol% photosensitizer was used.

Table S2. 2-pyridione ligands screening for C(sp^3)–H arylation functionalization using oxime ether directing groups.

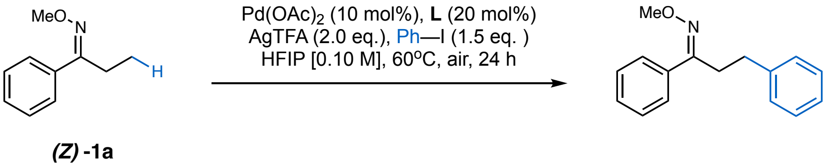

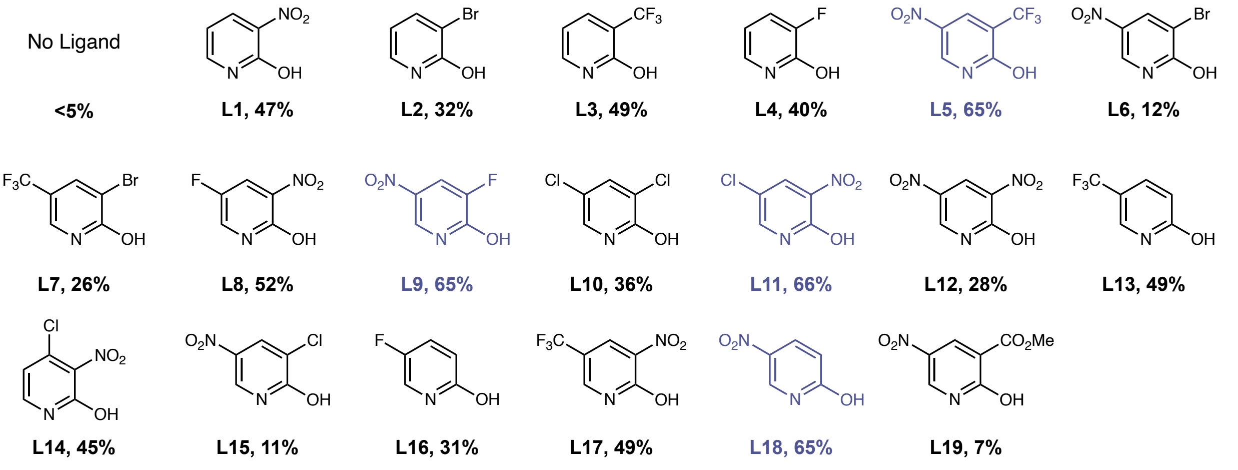

Note: % conversion was determined by GCMS

Table S3. Temperature and base screening for C(*sp*[3])–H arylation functionalization using oxime ether directing groups.

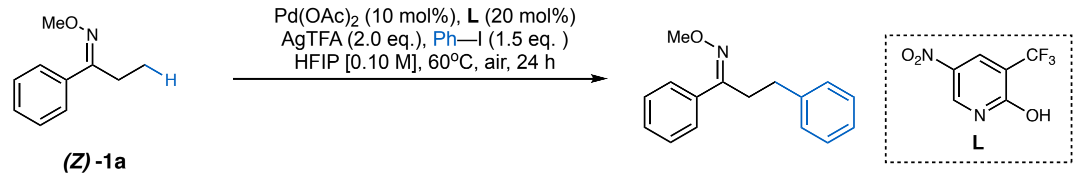

Entry	Temp. (°C)	Base (2 eq.)	SM (*Z*) (%)	SM (*E*) (%)	Pdt. (%)
1	30	AgTFA	72	0	27
2	50	AgTFA	21	0	78
3	60	AgTFA	10	20	70
4[a]	70	AgTFA	0	13	84
5[b]	55	AgTFA	0	0	90
6	55	AgOAc	37	33	30
7	55	Ag_2CO_3	49	18	32
8	55	Ag_3PO_4	23	72	0
9	55	CsOPiv	30	70	0
10	55	K_2CO_3	47	53	0
11	55	Cs_2CO_3	43	57	0
12	55	NaOAc	27	73	0
13	55	Bu_4NOH	64	36	0
14	55	$Bu_4NH_2PO_4$	18	82	0
15	55	none	17	83	0

[a] Z/E= 1 : 1 ratio of the product [b] 78% isolated yield

Table S4: Recycling Pd-catalyst after the first step of C(*sp*[2])–H acetoxylation.

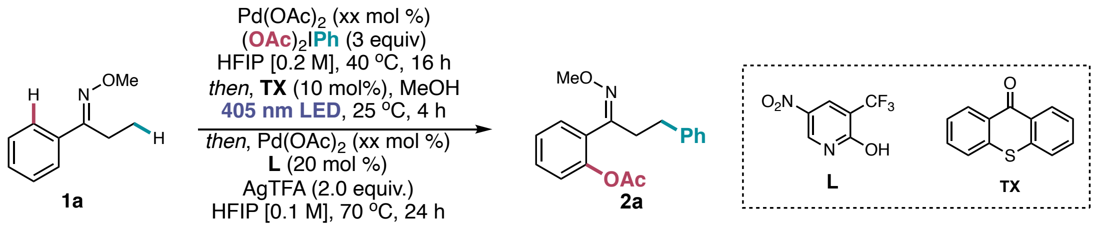

Entry	$Pd(OAc)_2$ (xx mol %) for C(sp^2)—H acetoxylation	$Pd(OAc)_2$ (xx mol %) for C(sp^3)—H arylation	NMR yield of **2a**
1	10	0	0
2	10	10	53
3	20	0	0

Reactions were performed on a 0.2 mmol scale. ^{1}H NMR yields calculated using CH_2Br_2 as an external standard.

Table S5: Optimization temperature and solvent concentration C(sp^2)–H acetoxylation/ methoxylation.

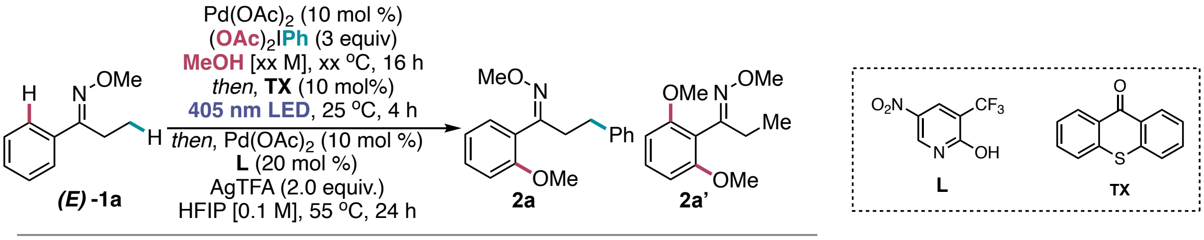

Entry	Temperature	Conc. of MeOH	2a NMR Yield, %	2a' NMR Yield, %
1	30 °C	0.2 M	47	5
2	40 °C	0.2 M	53	20
3	40 °C	0.1 M	35	26

Reactions were performed on a 0.2 mmol scale. ^{1}H NMR yields calculated using CH_2Br_2 as an external standard.

Table S6: Photocatalyst screening for Pd-photoredox C(sp^2)–H arylation using Ph_2IOTf.

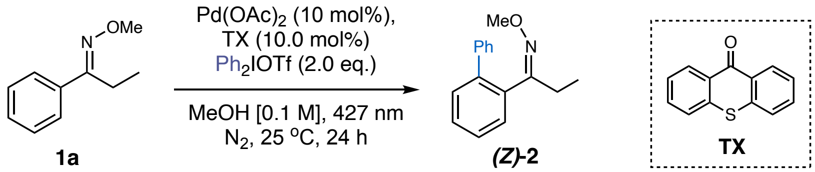

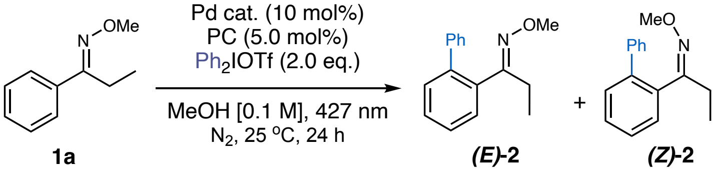

Entery	Pd cat.	PC	Z/E[a]	Yield[b]
1	$Pd(OAc)_2$	$Ir(ppy)_2(dtbbpy)PF_6$	0/100	35%
2	$Pd(OAc)_2$	$Ru(bpy)_3Cl_2.6H_2O$	0/100	60%
3	$Pd(OAc)_2$	$Ir[dF(CF_3)ppy]_2(dtbpy)PF_6$	**99/1**	38%
4	$Pd(OAc)_2$	$Ir[dF(CF_3)ppy]_2(dtbpy)PF_6$	99/1	**<5%**
5	$Pd(OAc)_2$	Thioxanthone[c]	**90/10**	**35%**
6	$Pd(NO_3)_2$	Thioxanthone	66/34	52%
7[d]	$Pd(OAc)_2$	Thioxanthone	17/83	31%
8[e]	$Pd(OAc)_2$	Thioxanthone	**45/55**	**44%**

[a] Z/E ratio was determined by GCMS.
[b] yield was determined by NMR.
[c] 10 mol% of TX and 390 nm were used
[d] 2 eq. of Cs_2CO_3 added as an additive.
[e] 2 eq. of 2,6-Lutidine added as an additive.

Mechanistic studies

To support the photoisomerization of the oxime-ether as the key-step in our proposed reaction mechanism, the absorption spectra of ***E*-(1a)**, ***Z*-(1a)**, and thioxanthone (**TX**) were collected (Figure **S1**).

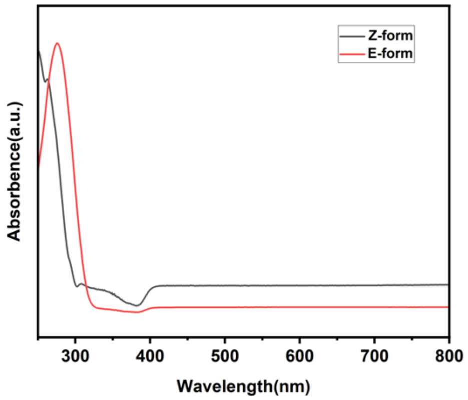

Figure S1: UV-Vis absorption spectra of the ***E*-(1a)**, ***Z*-(1a),** and thioxanthone (**TX**).

Results: The ***E*-(1a)** oxime shows no absorption in the visible region, excluding the possibility of a direct photoexcitation pathway. Instead, the **TX** photocatalyst is responsible for absorbing visible light and transferring energy to ***E*-(1a)**, thereby enabling photoisomerization. The absorption band edge of ***Z*-(1a)** is blue-shifted, indicating that the Z isomer has a less conjugated system than the *E* isomer. The reduced conjugation of the *Z* geometry further implies that the triplet state of the *Z* isomer has a higher energy (E_T) than that of the *E* isomer and the photocatalyst, thus preventing disfavored switching from *Z* back to *E*.

Additionally, the photoisomerization reaction was monitored by recording ^{1}H PhotoNMR studies over 75 minutes, as shown in Figure **S2**. The results indicate a gradual accumulation of the Z-isomer over time, reaching nearly complete switching after approximately one hour.

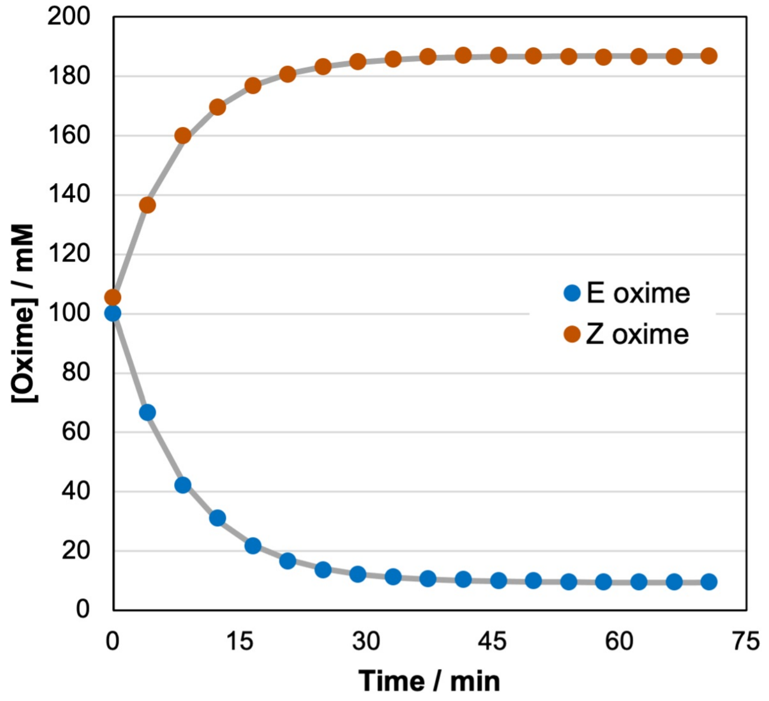

Figure S2: ^{1}H PhotoNMR experiment of a mixture of isomers (*E*:*Z*, 1:1)-**1a** under visible light irradiation.

Characterization Data of Starting Materials

Compounds **1a–b**, **1d–g**, and **1k–m** were prepared following General Procedure E, and their analytical data were consistent with previously reported values. [9-11] All hypervalent iodine reagents used in this study were synthesized according to literature methods, and their analytical data matched those previously reported. [12, 13]

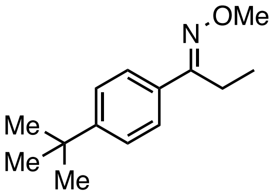

(E)-1-(4-(tert-butyl)phenyl)propan-1-one O-methyl oxime (1c)
Prepared according to general procedure **E**. The title compound was isolated via flash chromatography (gradient 0–10% EtOAc/hexanes) (3.9 g, 89% yield).
TLC (SiO_2) R_f = 0.65 in 9:1 hexanes/EtOAc
^{1}H NMR (400 MHz, $CDCl_3$) (δ, ppm): 7.60 – 7.53 (m, 2H), 7.43 – 7.35 (m, 2H), 3.97 (s, 3H), 2.74 (q, *J* = 7.6 Hz, 2H), 1.33 (s, 9H), 1.14 (t, *J* = 7.6 Hz, 3H).
^{13}C NMR (101 MHz, $CDCl_3$) (δ, ppm): 159.8, 152.3, 132.9, 126.1, 125.5, 61.9, 34.8, 31.4, 20.2, 11.4.
HRMS (ESI-TOF): *m/z* calculated for $C_{14}H_{22}NO$ $[M+H]^+$ = 220.1701; found 220.1706.

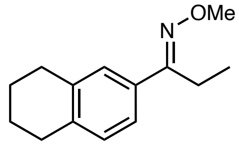

(*E*)-1-(5,6,7,8-tetrahydronaphthalen-2-yl)propan-1-one *O*-methyl oxime (1i)
Prepared according to general procedure **E**. The title compound was isolated via flash chromatography (gradient 0–20% EtOAc/hexanes) (1.3 g, 40 % yield).
TLC (SiO_2) R_f = 0.86 in 4:1 hexanes/EtOAc
^{1}H NMR (400 MHz, $CDCl_3$) (δ, ppm): 7.37 – 7.31 (m, 2H), 7.12 – 7.01 (m, 1H), 3.96 (s, 3H), 2.73 (dq, *J* = 15.2, 7.6, 7.1 Hz, 6H), 1.87 – 1.76 (m, 4H), 1.12 (t, *J* = 7.6 Hz, 3H).
^{13}C{H} NMR (101 MHz, $CDCl_3$) (δ, ppm): 160.2, 138.5, 137.4, 132.9, 129.3, 127.0, 123.5, 61.9, 29.4, 23.3, 20.2, 11.4.
HRMS (ESI-TOF): *m/z* calculated for $C_{14}H_{20}NO$ $[M+H]^+$ = 218.1545; found 218.1537.

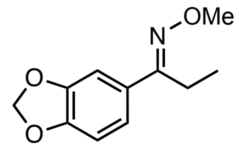

(E)-1-(benzo[d][1,3]dioxol-5-yl)propan-1-one O-methyl oxime (1j)
Prepared according to general procedure **E**. The title compound was isolated via flash chromatography (gradient 0–20% EtOAc/hexanes) (530 mg, 44 % yield).
TLC (SiO_2) R_f = 0.73 in 4:1 hexanes/EtOAc
^{1}H NMR (400 MHz, $CDCl_3$) (δ, ppm): 7.22 – 7.20 (m, 1H), 7.09 (dd, *J* = 8.2, 1.8 Hz, 1H), 6.79 (d, *J* = 8.1 Hz, 1H), 5.95 (s, 2H), 3.96 (s, 3H), 2.69 (q, *J* = 7.6 Hz, 2H), 1.12 (t, *J* = 7.6 Hz, 3H).

^{13}C{H} NMR (101 MHz, $CDCl_3$) (δ, ppm): 159.1, 148.4, 147.9, 129.8, 120.3, 108.0, 106.5, 101.2, 61.8, 20.0, 11.2.
HRMS (ESI-TOF): *m/z* calculated for $C_{11}H_{14}NO_3$ $[M+H]^+$ = 208.0974; found 208.0981.

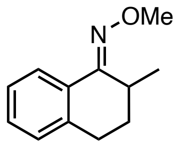

(*E*)-2-methyl-3,4-dihydronaphthalen-1(2*H*)-one *O*-methyl oxime (1n)
Prepared according to general procedure **E**. The title compound was isolated via flash chromatography (gradient 0–20% EtOAc/hexanes) (528 mg, 40% yield).
TLC (SiO_2) R_f = 0.83 in 9:1 hexanes/EtOAc
^{1}H NMR (400 MHz, $CDCl_3$) (δ, ppm): 8.15 (dq, *J* = 7.9, 1.7 Hz, 1H), 7.40 – 7.28 (m, 2H), 7.25 (d, *J* = 7.6 Hz, 1H), 4.15 – 4.04 (m, 3H), 3.68 (d, *J* = 3.7 Hz, 1H), 3.08 (s, 1H), 2.78 (d, *J* = 16.6 Hz, 1H), 2.15 – 2.02 (m, 1H), 1.89 – 1.78 (m, 1H), 1.31 (dt, *J* = 7.1, 1.7 Hz, 3H).
^{13}C NMR (101 MHz, $CDCl_3$) (δ, ppm): 157.8, 138.6, 130.1, 128.9, 128.7, 126.3, 124.7, 61.9, 28.5, 27.3, 25.1, 15.5.
HRMS (ESI-TOF): *m/z* calculated for $C_{12}H_{16}NO$ $[M+H]^+$ = 190.1232; found 190.1235.

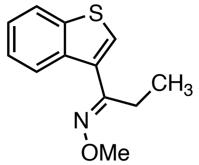

(*E*)-1-(benzo[*b*]thiophen-3-yl)propan-1-one *O*-methyl oxime (1o)
Prepared according to general procedure **E**. The title compound was isolated via flash chromatography (gradient 0–20% EtOAc/hexanes) (192 mg, 40% yield).
TLC (SiO_2) R_f = 0.86 in 4:1 hexanes/EtOAc
^{1}H NMR (400 MHz, $CDCl_3$) (δ, ppm): 8.65 (dt, *J* = 8.2, 1.0 Hz, 1H), 7.85 (dt, *J* = 7.9, 1.0 Hz, 1H), 7.60 (s, 1H), 7.46 – 7.35 (m, 2H), 4.07 (s, 3H), 2.83 (q, *J* = 7.6 Hz, 2H), 1.21 (t, *J* = 7.6 Hz, 3H).
^{13}C NMR (101 MHz, $CDCl_3$) (δ, ppm): 157.6, 140.9, 137.1, 132.0, 127.0, 126.45, 125.2, 125.2, 122.8, 62.4, 21.8, 11.9.
HRMS (ESI-TOF): *m/z* calculated for $C_{12}H_{14}NOS$ $[M+H]^+$ = 220.0796; found 220.0800.

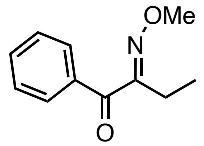

(*E*)-2-(methoxyimino)-1-phenylbutan-1-one (1p)
Prepared according to general procedure **F**. The title compound was isolated via flash chromatography (gradient 0–20% EtOAc/hexanes) (385 mg, 35 % yield).
TLC (SiO_2) R_f = 0.69 in 9:1 hexanes/EtOAc
^{1}H NMR (500 MHz, $CDCl_3$) (δ, ppm): 7.98 – 7.94 (m, 2H), 7.57 – 7.52 (m, 1H), 7.43 (dd, J = 8.5, 7.1 Hz, 2H), 4.03 (s, 3H), 2.69 (q, J = 7.6 Hz, 2H), 1.12 (t, J = 7.6 Hz, 3H).

^{13}C NMR (101 MHz, CDCl$_3$) (δ, ppm): 191.21, 159.81, 136.77, 132.67, 130.63, 127.99, 63.05, 18.51, 10.51.

HRMS (ESI-TOF): *m/z* calculated for $C_{11}H_{14}NO_2$ $[M+H]^+$ = 192.1025; found 192.1024.

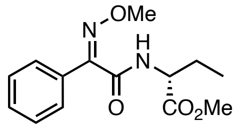

Methyl (*R*,*Z*)-2-(2-(methoxyimino)-2-phenylacetamido)butanoate (1q)
Prepared according to general procedure **M**. The title compound was isolated via flash chromatography (gradient 0–20% EtOAc/hexanes) (436 mg, 40% yield).
TLC (SiO_2) R_f = 0.72 in 9:1 hexanes/EtOAc
^{1}H NMR (500 MHz, CDCl$_3$) (δ, ppm): 7.67 – 7.61 (m, 2H), 7.39 – 7.33 (m, 3H), 6.60 (d, *J* = 8.7 Hz, 1H), 4.83 – 4.69 (m, 1H), 4.03 – 3.98 (m, 3H), 3.78 – 3.74 (m, 3H), 2.08 – 1.99 (m, 1H), 1.84 – 1.75 (m, 1H), 0.95 (td, *J* = 7.5, 3.4 Hz, 3H).
^{13}C NMR (101 MHz, CDCl$_3$) (δ, ppm): 172.2, 162.6, 152.4, 131.2, 130.1, 128.6, 126.7, 62.9, 62.8, 53.5, 53.3, 52.5, 25.5, 9.4.
HRMS (ESI-TOF): *m/z* calculated for $C_{14}H_{19}N_2O_4$ $[M+H]^+$ = 279.1345; found 279.1332.

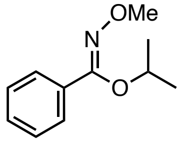

Isopropyl (Z)-N-methoxybenzimidate (1r)
Prepared according to general procedure **G**. The title compound was isolated via flash chromatography (gradient 0–20% EtOAc/hexanes) (465 mg mg, 40% yield).
TLC (SiO_2) R_f = 0.60 in 9:1 hexanes/EtOAc
^{1}H NMR (500 MHz, CDCl$_3$) (δ, ppm): 7.70 (dd, J = 7.8, 1.9 Hz, 2H), 7.39 – 7.34 (m, 3H), 4.87 – 4.80 (m, 1H), 3.91 (s, 3H), 1.32 (d, J = 6.3 Hz, 6H).
^{13}C NMR (101 MHz, CDCl$_3$) (δ, ppm): 153.7, 131.9, 129.8, 128.3, 127.1, 74.0, 62.3, 22.5.
HRMS (ESI-TOF): *m/z* calculated for $C_{11}H_{15}NO_2$ $[M+H]^+$ = 194.1181; found 194.1181.

Characterization Data of Difunctionalized Products

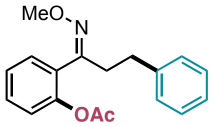

(*Z*)-2-(1-(methoxyimino)-3-phenylpropyl)phenyl acetate (2a)

Prepared according to general procedure **A**. The title compound was isolated via flash chromatography (gradient 0 –20% EtOAc/hexanes) as a light-yellow oil (32 mg, 44% yield) with a Z:E ratio of 8:1.

TLC (SiO_2) R_f = 0.65 in 4:1 hexanes/EtOAc

^{1}H NMR (500 MHz, $CDCl_3$) (δ, ppm): 7.37 (td, *J* = 7.8, 1.7 Hz, 1H), 7.24 (dd, *J* = 6.0, 2.1 Hz, 3H), 7.16 (ddd, *J* = 7.8, 6.3, 2.1 Hz, 5H), 3.77 (s, 3H), 2.76 (dddd, *J* = 9.1, 7.3, 5.0, 1.7 Hz, 4H), 2.18 (s, 3H).

^{13}C{H} NMR (126 MHz, $CDCl_3$) (δ, ppm): 169.0, 154.2, 147.2, 141.2, 129.7, 128.6, 128.5, 128.2, 127.9, 126.2, 125.9, 123.0, 61.9, 37.1, 32.7, 21.1.

HRMS (ESI TOF): m/z calculated for $C_{18}H_{20}NO_3$ $[M+H]^+$ = 298.1436; found 298.1443.

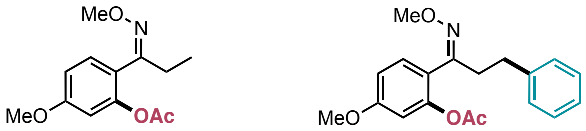

2b-P$_1$ **2b-P$_2$**

(*Z*)-5-methoxy-2-(1-(methoxyimino)-3-phenylpropyl)phenyl acetate (2b)

Prepared according to general procedure **A**. The title compound was isolated via flash chromatography (gradient 0–20% EtOAc/hexanes) as a yellow oil (22 mg, 33% yield, 2b-P$_1$: 2b-P$_2$ = 1:2). NMRs include the mixture of the desired product (2b-P$_2$) and product from the second step (2b-P$_1$).

TLC (SiO_2) Rf=0.45 in 4:1 hexanes/EtOAc

^{1}H **NMR (400 MHz,$CDCl_3$) (δ, ppm):** 7.25 (d, *J* = 0.7 Hz, 1H), 7.21 – 7.15 (m, 3H), 7.09 (d, *J* = 8.6 Hz, 1H), 6.81 (ddd, *J* = 8.5, 2.5, 0.7 Hz, 1H), 6.72 (dd, *J* = 4.0, 2.5 Hz, 1H), 3.81 (s, 3H), 3.80 (s, 3H), 3.79 (s, 3H), 3.78 (s, 3H), 2.81 – 2.72 (m, 4H), 2.46 (q, *J* = 7.5 Hz, 2H), 2.25 (s, 3H), 2.20 (s, 3H), 1.05 (t, *J* = 7.5 Hz, 2H).

^{13}C{H} NMR (101 MHz, $CDCl_3$) (δ, ppm): δ 168.9, 168.9, 160.5, 160.4, 156.0, 154.1, 148.3, 148.2, 141.3, 128.9, 128.8, 128.F6, 128.5, 126.1, 120.0, 119.8, 112.1, 112.0, 108.7, 108.7, 61.8, 61.7, 55.6, 55.6, 37.2, 32.8, 29.8, 28.9, 21.2, 21.1, 11.3.

HRMS (ESI TOF): m/z calculated for $C_{19}H_{21}NO_4$ $[M+H]^+$ = 328.1549; found 328.1539.

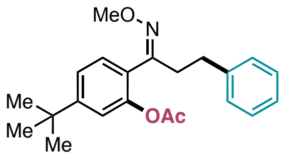

(Z)-5-(tert-butyl)-2-(1-(methoxyimino)-3-phenylpropyl)phenyl acetate (2c)

Prepared according to general procedure **A**. The title compound was isolated via flash chromatography (gradient 0 –20% EtOAc/hexanes) as a light-yellow oil (25 mg, 35% yield).

TLC (SiO_2) R_f = 0.46 in 4:1 hexanes/EtOAc

^{1}H NMR (400 MHz, CD_2Cl_2) (δ, ppm): 7.32 – 7.24 (m, 3H), 7.22 – 7.16 (m, 3H), 7.15 – 7.10 (m, 2H), 3.75 (s, 3H), 2.83 – 2.76 (m, 2H), 2.76 – 2.70 (m, 2H), 2.18 (s, 3H), 1.33 (d, *J* = 1.7 Hz, 9H).
^{13}C{H} NMR (101 MHz, $CDCl_3$) (δ, ppm): 169.1, 154.4, 153.6, 147.1, 141.3, 128.6, 128.5, 127.7, 126.1, 124.7, 123.1, 120.1, 61.9, 37.2, 35.0, 32.8, 31.3, 21.2.
HRMS (ESI TOF): m/z calculated for $C_{22}H_{28}NO_3$ $[M+H]^+$ = 354.2069; found 354.2054.

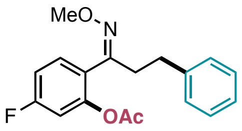

(Z)-5-fluoro-2-(1-(methoxyimino)-3-phenylpropyl)phenyl acetate (2d)
Prepared according to general procedure **A**. The title compound was isolated via flash chromatography (gradient 0 –20% EtOAc/hexanes) as a light-yellow oil (21 mg, 33% yield).
TLC (SiO_2) R_f = 0.40 in 4:1 hexanes/EtOAc
^{1}H NMR (400 MHz, $CDCl_3$) (δ, ppm): 7.28 – 7.24 (m, 2H), 7.19 (dd, *J* = 7.7, 5.8 Hz, 3H), 7.13 (dd, *J* = 8.5, 6.0 Hz, 1H), 6.98 (ddd, *J* = 15.5, 8.7, 2.5 Hz, 2H), 3.79 (s, 3H), 2.87 – 2.66 (m, 4H), 2.19 (s, 3H).
^{13}C{H} NMR (101 MHz, $CDCl_3$) (δ, ppm): 168.5, 162.6 (d, *J* = 249.5 Hz),153.4, 148.2 (d, *J* = 10.9 Hz), 141.0, 129.2 (d, *J* = 9.5 Hz), 128.5 (d, *J* = 5.4 Hz), 126.2, 123.8 (d, *J* = 3.7 Hz), 113.2 (d, *J* = 21.6 Hz), 111.1 (d, *J* = 24.6 Hz), 61.9, 37.0, 32.6, 21.0.
^{19}F NMR (377 MHz, $CDCl_3$) (δ, ppm): -112.66.
HRMS (ESI TOF): m/z calculated for $C_{18}H_{19}FNO_3$ $[M+H]^+$ = 316.1349; found 316.1337.

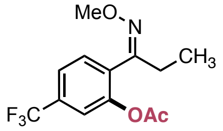

2e-P1

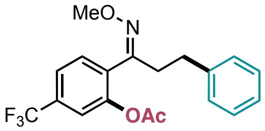

2e-P2

(*Z*)-2-(1-(methoxyimino)-3-phenylpropyl)-5-(trifluoromethyl) phenyl acetate (2e)
Prepared according to general procedure **A**. The title compound was isolated via flash chromatography (gradient 0–20% EtOAc/hexanes) as a colorless oil (13 mg, 18% yield, 2e-P_1:2e-P_2 = 1:1.4). NMRs include the mixture of the desired product (2e-P_2) and product from the second step (2e-P_1).
TLC (SiO_2) Rf = 0.53 in 4:1 hexanes/EtOAc
^{1}H NMR (400 MHz, $CDCl_3$) (δ, ppm): 7.57 (ddd, *J* = 7.9, 6.1, 1.7 Hz, 2H), 7.52 (d, *J* = 1.7 Hz, 2H), 7.37 – 7.30 (m, 5H), 7.25 – 7.22 (m, 3H), 3.86 (s, 3H), 3.84 (s, 3H), 2.91 – 2.81 (m, 4H), 2.54 (q, *J* = 7.5 Hz, 4H), 2.33 (s, 3H), 2.27 (s, 3H), 1.13 (t, *J* = 7.5 Hz, 3H).
^{13}C NMR (101 MHz, $CDCl_3$) (δ, ppm): 168.8, 168.7, 155.8, 153.28, 147.58 (d, *J* = 6.5 Hz), 141.1, 132.45 – 131.49 (m), 129.19 (d, *J* = 7.8 Hz), 128.82 (d, *J* = 3.0 Hz), 126.9, 126.6, 124.81 (d, *J* = 3.3 Hz), 123.02 (q, *J* = 3.6 Hz), 122.65 (d, *J* = 3.4 Hz), 120.72 (dq, *J* = 7.7, 3.8 Hz), 62.3, 62.2, 36.9, 32.8, 30.1, 28.9, 21.3, 21.2, 11.3.
^{19}F NMR (471 MHz, $CDCl_3$) (δ, ppm): -62.73 and -62.75.
HRMS (ESI TOF): m/z calculated for $C_{19}H_{18}F_3NO_3$ $[M+H]^+$ = 366.1317; found 366.1306.

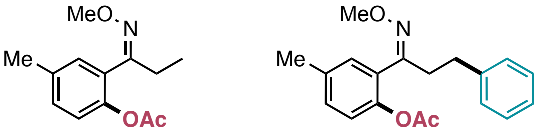

2f-P$_1$ **2f-P$_2$**

(Z)-2-(1-(methoxyimino)-3-phenylpropyl)-6-methylphenyl acetate (2f)

Following General Procedure **A**, except the first and last step is done at 60 and 75 °C, respectively. The title compound was isolated via flash chromatography gradient 0 –20% EtOAc/hexanes as a colorless liquid (20 mg, 32% yield, 2f-P$_1$: 2f-P$_2$ = 1:6). NMRs include the mixture of the desired product (2f-P$_2$) and product from the second step (2f-P$_1$).

TLC (SiO_2) R_f = 0.54 in 4:1 hexanes/EtOAc

^{1}H NMR (500 MHz, $CDCl_3$) (δ, ppm): 7.28 – 7.26 (m, 1H), 7.25 (s, 1H), 7.21 – 7.16 (m, 4H), 7.05 (dd, *J* = 8.3, 4.4 Hz, 1H), 6.94 (d, *J* = 2.2 Hz, 1H), 3.80 (s, 3H), 3.78 (s, 3H) 2.82 – 2.74 (m, 4H), 2.46 (q, *J* = 7.5 Hz, 2H), 2.34 (s, 3H), 2.24 (s, 3H), 2.18 (s, 3H), 1.06 (t, *J* = 7.5 Hz, 1H).

^{13}C NMR (126 MHz, $CDCl_3$) (δ, ppm): 169.2, 154.4, 145.0, 141.3, 135.7, 130.4, 128.6, 128.5, 127.6, 126.2, 122.7, 61.9, 37.1, 32.7, 29.9, 21.1, 11.2.

HRMS (ESI-TOF): *m/z* calculated for $C_{19}H_{22}NO_3$ $[M+H]^+$ = 312.1600; found 312.1589.

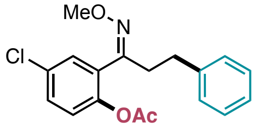

(*Z*)-4-chloro-2-(1-(methoxyimino)-3-phenylpropyl) phenyl acetate (2g)

Prepared according to general procedure **A**. The title compound was isolated via flash chromatography (gradient 0–20% EtOAc/hexanes) as a white solid (17 mg, 26% yield).

TLC (SiO_2) R_f= 0.81 in 4:1 hexanes/EtOAc

^{1}H NMR (500 MHz, $CDCl_3$) (δ, ppm): δ 7.34 (dd, *J* = 8.7, 2.6 Hz, 1H), 7.28 (d, *J* = 7.9 Hz, 2H), 7.19 (td, *J* = 5.4, 2.8 Hz, 3H), 7.13 – 7.10 (m, 2H), 3.79 (s, 3H), 2.83 – 2.73 (m, 4H), 2.18 (s, 3H).

^{13}C NMR (126 MHz, $CDCl_3$) (δ, ppm): δ 168.6, 152.7, 145.6, 140.7, 131.2, 129.5, 129.3, 128.5, 128.4, 128.0, 127.9, 126.1, 124.2, 124.1, 61.8, 36.6, 32.4, 31.9, 29.7, 20.9, 14.1, 11.0.

HRMS (ESI-TOF): *m/z* calculated for $C_{18}H1_8ClNO_3$ $[M+H]^+$ = 332.1053; found 332.1046.

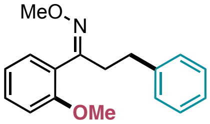

(Z)-1-(2-methoxyphenyl)-3-phenylpropan-1-one O-methyl oxime (2h)

Prepared according to the general procedure **A** except that MeOH was used as a solvent instead of HFIP in the first step. The title compound was isolated by flash chromatography (gradient 0–20% EtOAc/hexanes) to afford the product (21 mg, 38% yield) with a Z:E ratio of 9:1.

TLC (SiO_2) R_f = 0.36 in 4:1 hexanes/EtOAc

^{1}H NMR (400 MHz, $CDCl_3$) (δ, ppm): 7.72 – 7.67 (m, 2H), 7.36 (dd, *J* = 5.1, 2.0 Hz, 3H), 7.22 – 7.14 (m, 2H), 6.91 – 6.82 (m, 2H), 3.98 (s, 3H), 3.85 (s, 3H), 3.04 – 2.96 (m, 2H), 2.90 – 2.81 (m, 2H).

^{13}C{H} NMR (101 MHz, $CDCl_3$) (δ, ppm): 158.4, 157.6, 136.0, 130.0, 129.1, 128.5, 127.5, 126.7, 120.6, 110.3, 62.1, 55.4, 27.7, 27.2.

HRMS (ESI-TOF): *m/z* calculated for $C_{17}H_{20}NO_2$ $[M+H]^+$ = 270.1494; found 270.1485.

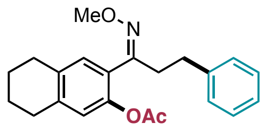

(*Z*)-3-(1-(methoxyimino)-3-phenylpropyl)-5,6,7,8-tetrahydronaphthalen-2-yl acetate (2i)
Prepared according to general procedure **A**. The title compound was isolated via flash chromatography (gradient 0 –20% EtOAc/hexanes) as a yellow oil (20 mg, 29% yield).
TLC (SiO_2) R_f = 0.36 in 4:1 hexanes/EtOAc
^{1}H NMR (400 MHz, CD_2Cl_2) (δ, ppm): 7.30 – 7.24 (m, 2H), 7.23 – 7.15 (m, 3H), 6.84 (s, 2H), 3.74 (s, 3H), 2.81 – 2.70 (m, 8H), 2.15 (s, 3H), 1.80 (dq, J = 5.9, 3.3 Hz, 4H).
^{13}C{H} NMR (101 MHz, CD_2Cl_2) (δ, ppm): 169.5, 154.6, 145.1, 141.8, 139.3, 135.2, 128.9, 128.7, 126.3, 125.4, 123.2, 61.8, 37.4, 32.8, 29.7, 29.2, 23.4, 23.2, 21.2.
HRMS (ESI TOF): *m/z* calculated for $C_{22}H_{26}NO_3$ $[M+H]^+$ = 352.1913; found 352.1901.

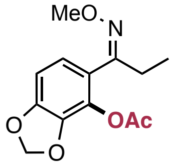

2j-P$_1$

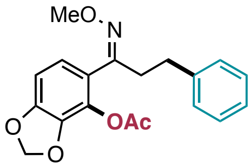

2j-P$_2$

(Z)-5-(1-(methoxyimino)-3-phenylpropyl)benzo[d][1,3]dioxol-4-yl acetate (2j)
Following General Procedure **A**. The title compound was isolated via flash chromatography (gradient 0 –20% EtOAc/hexanes) as a yellow oil (21 mg, 31% yield, 2j-P$_1$: 2j-P$_2$ = 1:3). NMRs include the mixture of the desired product (2j-P$_2$) and product from the second step (2j-P$_1$).

TLC (SiO_2) Rf = 0.14 in Hex: EtOAc = 9:1.
^{1}H NMR (400 MHz, $CDCl_3$) (δ, ppm): 7.28 (dd, J = 7.5, 1.0 Hz, 1H), 7.25 (d, J = 0.7 Hz, 1H), 7.21 – 7.16 (m, 3H), 6.77 – 6.74 (m, 1H), 6.64 (dd, J = 8.0, 4.4 Hz, 1H), 6.03 (s, 2H), 6.02 (s, 2H), 3.79 (s, 3H), 3.79 (s, 3H), 2.80 – 2.72 (m, 4H), 2.47 – 2.45 (q, 2H), 2.26 (s, 3H), 2.21 (s, 3H), 1.05 (s, 3H).
^{13}C NMR (101 MHz, $CDCl_3$) (δ, ppm): 167.1, 153.6, 149.5, 141.0, 139.5, 130.7, 128.5, 128.4, 126.0, 122.3, 120.6, 120.4, 106.3, 106.3, 102.2, 102.1, 61.7, 37.4, 37.1, 32.7, 29.7, 29.1, 20.4, 11.2.
HRMS (ESI-TOF): *m/z* calculated for $C_{19}H_{20}NO_5$ $[M+H]^+$ = 342.1341; found 342.1331.

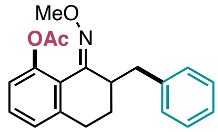

(*Z*)-7-benzyl-8-(methoxyimino)-5,6,7,8-tetrahydronaphthalen-1-yl acetate (2n)
Following General Procedure **A**. The title compound was isolated via flash chromatography (gradient 0 –20% EtOAc/hexanes) (28 mg, 44% yield) with a Z:E ratio of 7:1.
TLC (SiO_2) Rf = 0.75 in Hex: EtOAc = 9:1.
^{1}H NMR (500 MHz, $CDCl_3$) (δ, ppm): 7.34 (t, J = 7.8 Hz, 1H), 7.28 (dd, J = 7.0, 1.5 Hz, 2H), 7.22 – 7.18 (m, 3H), 7.07 – 7.04 (m, 2H), 3.79 (s, 3H), 3.10 – 3.03 (m, 1H), 2.92 (dd, J = 13.9, 6.5 Hz, 1H), 2.76 (dt, J = 15.9, 5.5 Hz, 1H), 2.66 – 2.57 (m, 2H), 2.24 (s, 3H), 2.12 – 2.06 (m, 1H), 1.37 (t, J = 7.2 Hz, 1H).
^{13}C NMR (126 MHz, $CDCl_3$) (δ, ppm): 169.2, 153.2, 148.4, 142.4, 139.8, 130.1, 129.5, 128.30, 126.2, 124.9, 120.9, 61.8, 41.7, 29.9, 27.9, 22.8, 21.4, 14.3.

HRMS (ESI-TOF): *m/z* calculated for $C_{20}H_{22}NO_3$ $[M+H]^+$ = 324.1600; found 324.1588.

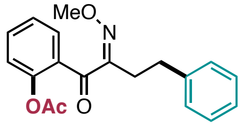

(Z)-2-(2-(methoxyimino)-4-phenylbutanoyl)phenyl acetate (2p)
Following General Procedure **C**. The title compound was isolated via flash chromatography (gradient 0 –20% EtOAc/hexanes) (19 mg, 30 % yield). The NMR spectra show a mixture of the desired product and a small amount of acetoxylated product after second step.
TLC (SiO_2) R*f* = 0.35 in Hex: EtOAc = 4:1.
^{1}H NMR (500 MHz, $CDCl_3$) (δ, ppm): 7.83 (dd, *J* = 7.9, 1.7 Hz, 1H), 7.61 (tt, *J* = 7.7, 2.0 Hz, 2H), 7.38 – 7.32 (m, 2H), 7.28 (d, *J* = 8.2 Hz, 1H), 7.21 – 7.19 (m, 2H), 7.15 (d, *J* = 1.2 Hz, 1H), 3.73 (s, 3H), 2.94 – 2.88 (m, 2H), 2.75 – 2.69 (m, 2H), 2.36 (s, 3H).
^{13}C NMR (126 MHz, $CDCl_3$) (δ, ppm): 192.7, 192.4, 169.1, 159.2, 157.4, 150.2, 140.5, 134.9, 131.9, 128.4, 126.9, 126.3, 124.1, 61.9, 32.0, 29.7, 24.7, 21.0, 10.3.
HRMS (ESI-TOF): *m/z* calculated for $C_{19}H_{20}NO_4$ $[M+H]^+$ = 326.1392; found 326.1385.

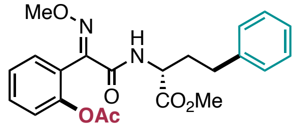

Methyl (*R*,E)-2-(2-(2-acetoxyphenyl)-2-(methoxyimino)acetamido)-4-phenylbutanoate (2q)
Following General Procedure **D**. The title compound was isolated via flash chromatography (gradient 0 –20% EtOAc/hexanes) as a yellow oil (18 mg, 46 % yield).
TLC (SiO_2) R*f* = 0.47 in Hex: EtOAc = 9:1.
^{1}H NMR (500 MHz, $CDCl_3$) (δ, ppm): 7.44 (ddd, J = 8.2, 7.2, 2.0 Hz, 1H), 7.33 – 7.27 (m, 4H), 7.24 – 7.18 (m, 5H), 4.74 (td, J = 7.7, 5.2 Hz, 1H), 4.00 (s, 3H), 3.75 (s, 3H), 2.71 (dt, J = 9.2, 6.3 Hz, 2H), 2.30 – 2.25 (m, 1H), 2.20 (s, 3H), 2.13 – 2.07 (m, 1H).
^{13}C NMR (126 MHz, $CDCl_3$) (δ, ppm): 172.6, 168.5, 161.7, 148.0, 147.4, 140.6, 130.6, 130.4, 128.5, 128.4, 126.3, 125.3, 122.7, 122.2, 63.6, 52.5, 52.1, 33.9, 31.7, 21.0.
HRMS (ESI-TOF): *m/z* calculated for $C_{22}H_{25}N_2O_6$ $[M+H]^+$ = 413.1713; found 413.1704.

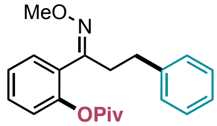

(Z)-2-(1-(methoxyimino)-3-phenylpropyl)phenyl pivalate (2s)
Prepared according to general procedure **A**. The title compound was isolated via flash chromatography (gradient 0 –20% EtOAc/hexanes) (31 mg, 47% yield).
TLC (SiO_2) R_f = 0.36 in 4:1 hexanes/EtOAc
^{1}H NMR (400 MHz, CD_2Cl_2) (δ, ppm): 7.43 – 7.36 (m, 1H), 7.31 – 7.24 (m, 3H), 7.22 – 7.11 (m, 5H), 3.76 (s, 3H), 2.87 – 2.78 (m, 2H), 2.78 – 2.69 (m, 2H), 1.32 (s, 9H).
^{13}C{H} NMR (101 MHz, CD_2Cl_2) (δ, ppm): 176.7, 154.3, 147.8, 141.8, 129.7, 128.7, 128.3, 126.4, 125.9, 123.1, 61.9, 39.3, 37.6, 32.8, 27.3.
HRMS (ESI-TOF): *m/z* calculated for $C_{21}H_{26}NO_3$ $[M+H]^+$ = 340.1913; found 340.1906.

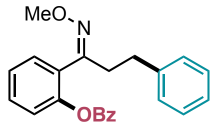

(Z)-2-(1-(methoxyimino)-3-phenylpropyl)phenyl benzoate (2t)

Prepared according to general procedure **A**. The title compound was isolated via flash chromatography (gradient 0 –20% EtOAc/hexanes) (21 mg, 29% yield) with a Z:E ratio of 16:1.

TLC (SiO_2) R_f = 0.40 in 4:1 hexanes/EtOAc

^{1}H NMR (400 MHz, $CDCl_3$) (δ, ppm): 8.11 – 8.06 (m, 2H), 7.66 – 7.60 (m, 1H), 7.52 – 7.43 (m, 3H), 7.40 – 7.30 (m, 2H), 7.27 – 7.22 (m, 3H), 7.20 – 7.14 (m, 3H), 3.64 (s, 3H), 2.83 (d, *J* = 1.6 Hz, 4H).

^{13}C{H} NMR (101 MHz, $CDCl_3$) (δ, ppm): 164.7, 154.0, 147.5, 141.1, 133.7, 130.3, 129.8, 129.7, 128.7, 128.6, 128.5, 127.7, 126.2, 126.0, 123.3, 61.8, 37.1, 32.8.

HRMS (ESI TOF): *m/z* calculated for $C_{23}H_{22}NO_3$ $[M+H]^+$ = 360.1600; found 360.1590.

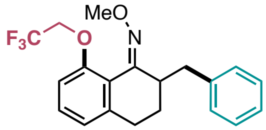

(*Z*)-2-benzyl-8-(2,2,2-trifluoroethoxy)-3,4-dihydronaphthalen-1(2*H*)-one *O*-methyl oxime (2u)

Following General Procedure **A**. The title compound was isolated via flash chromatography (gradient 0 –20% EtOAc/hexanes) (19 mg**,** 18% yield).

TLC (SiO_2) R*f* = 0.18 in Hex: EtOAc = 9:1.

^{1}H NMR (500 MHz, $CDCl_3$) (δ, ppm): 7.31 – 7.27 (m, 1H), 7.24 (s, 2H), 7.19 (d, *J* = 7.6 Hz, 3H), 6.89 (d, *J* = 7.5 Hz, 1H), 6.80 (d, *J* = 8.3 Hz, 1H), 4.37 (dtt, *J* = 19.5, 11.2, 8.2 Hz, 2H), 3.82 (s, 3H), 3.14 – 3.01 (m, 1H), 2.91 (s, 1H), 2.70 (dt, *J* = 15.7, 5.2 Hz, 1H), 2.57 (ddd, *J* = 21.6, 12.0, 7.1 Hz, 2H), 2.12 (tt, *J* = 12.6, 5.8 Hz, 1H), 1.46 (s, 1H).

^{13}C{H} NMR (126 MHz, $CDCl_3$) (δ, ppm): 155.0, 152.8, 143.1, 130.34, 129.56, 128.28, 126.17, 124.68, 122.47, 121.70, 110.96, 66.36 (q, *J* = 35.6 Hz), 61.72, 41.84, 29.85, 28.39, 28.19.

^{19}F NMR (471 MHz, $CDCl_3$) (δ, ppm): -73.82.

HRMS (ESI TOF): *m/z* calculated for $C_{20}H_{21}F_3NO_2$ $[M+H]^+$ = 364.1519; found 364.1524.

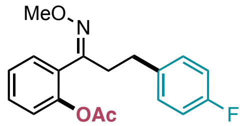

(Z)-2-(4-(4-fluorophenyl)-2-(methoxyimino)butanoyl)phenyl acetate (2v)

Following General Procedure **A**. The title compound was isolated via flash chromatography (gradient 0 –20% EtOAc/hexanes) (19 mg**,** 2% yield).

TLC (SiO_2) R*f* = 0.44 in Hex: EtOAc = 4:1.

^{1}H NMR (400 MHz, $CDCl_3$) (δ, ppm): 7.42 – 7.36 (m, 1H), 7.28 (dd, *J* = 7.5, 1.2 Hz, 1H), 7.19 – 7.11 (m, 4H), 6.95 (t, *J* = 8.7 Hz, 2H), 3.79 (s, 3H), 2.82 – 2.71 (m, 4H), 2.20 (s, 3H).

^{13}C{H} NMR (101 MHz, $CDCl_3$) (δ, ppm): 169.1, 162.7, 160.3 (d, *J* = 242.9 Hz), 154.0, 147.2, 136.7 (d, *J* = 3.2 Hz), 130.0 (d, *J* = 7.8 Hz), 129.8, 128.2 (d, *J* = 26.7 Hz), 126.0, 123.0, 115.3 (d, *J* = 21.2 Hz), 61.9, 37.1, 31.8, 21.1.

^{19}F NMR (377 MHz, $CDCl_3$) (δ, ppm): -117.6.

HRMS (ESI-TOF): *m/z* calculated for $C_{18}H_{19}FNO_3$ $[M+H]^+$ = 316.1349; found 316.1340.

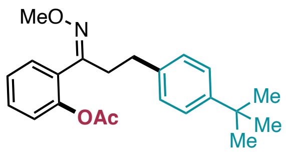

(*Z*)-2-(3-(4-(*tert*-butyl)phenyl)-1-(methoxyimino)propyl)phenyl acetate (2w)
Following General Procedure **A**. The title compound was isolated via flash chromatography (Hex:DCM:Et_2O 7:2:1 to 5:3:2) (18 mg, 26 % yield).
TLC (SiO_2) R*f* = 0.34 in Hex: EtOAc = 8:1.
^{1}H NMR (500 MHz, $CDCl_3$) (δ, ppm): 7.38 (td, *J* = 7.8, 1.6 Hz, 1H), 7.29 (d, *J* = 8.0 Hz, 3H), 7.19 – 7.15 (m, 2H), 7.12 (d, *J* = 8.0 Hz, 2H), 3.80 (s, 3H), 2.77 (s, 4H), 2.19 (s, 3H), 1.30 (s, 9H).
^{13}C NMR (126 MHz, $CDCl_3$) (δ, ppm): 168.8, 154.3, 148.8, 147.1, 137.9, 129.5, 128.1, 127.8, 125.8, 125.2, 122.9, 61.7, 36.9, 34.4, 32.0, 31.4, 30.9, 20.9.
HRMS (ESI-TOF): *m/z* calculated for $C_{22}H_{28}NO_3$ $[M+H]^+$ = 354.2064; found 354.2069.

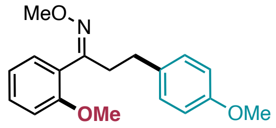

(*Z*)-1-(2-methoxyphenyl)-3-(4-methoxyphenyl)propan-1-one *O*-methyl oxime (2x)
Prepared according to the general procedure **A**, except that MeOH was used as a solvent instead of HFIP in the first step. The title compound was isolated via flash chromatography (gradient 0 – 20% EtOAc/hexanes) (30 mg, 40% yield).
TLC (SiO_2) R_f = 0.59 in 4:1 hexanes/EtOAc
^{1}H NMR (500 MHz, $CDCl_3$) (δ, ppm): 7.33 (ddd, *J* = 8.3, 7.4, 1.8 Hz, 1H), 7.11 – 7.05 (m, 3H), 6.97 (ddd, *J* = 21.5, 7.8, 0.9 Hz, 2H), 6.81 (d, *J* = 8.6 Hz, 2H), 3.82 (s, 6H), 3.78 (s, 3H), 2.81 – 2.76 (m, 2H), 2.76 – 2.69 (m, 2H).
^{13}C{H} NMR (126 MHz, $CDCl_3$) (δ, ppm): 157.9, 156.7, 155.8, 133.7, 129.9, 129.4, 129.0, 124.0, 120.5, 113.8, 111.3, 61.8, 55.7, 55.4, 37.1, 32.0.
HRMS (ESI TOF): *m/z* calculated for $C_{18}H_{22}NO_3$ $[M+H]^+$ = 300.1600; found 300.1593.

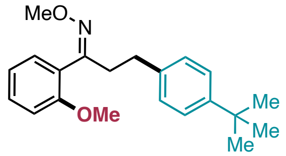

(*Z*)-3-(4-(*tert*-butyl)phenyl)-1-(2-methoxyphenyl)propan-1-one *O*-methyl oxime (2y)
Prepared according to the general procedure **A**, except that MeOH was used as a solvent instead of HFIP in the first step. The title compound was isolated via flash chromatography (gradient 0–20% EtOAc/hexanes) (23 mg, 35% yield).
TLC (SiO_2) R_f = 0.39 in 4:1 hexanes/EtOAc
^{1}H NMR (500 MHz, $CDCl_3$) (δ, ppm): 7.32 (ddd, *J* = 8.4, 7.4, 1.8 Hz, 1H), 7.28 (d, *J* = 8.3 Hz, 2H), 7.12 – 7.09 (m, 2H), 7.08 (dd, *J* = 7.5, 1.8 Hz, 1H), 6.98 (td, *J* = 7.4, 1.0 Hz, 1H), 6.95 – 6.92 (m, 1H), 3.82 (s, 3H), 3.81 (s, 3H), 2.82 – 2.74 (m, 4H), 1.30 (s, 9H).
^{13}C NMR (151 MHz, $CDCl_3$) (δ, ppm): 156.7, 155.7, 148.7, 138.4, 129.7, 128.9, 128.0, 125.2, 123.9, 120.3, 111.1, 61.6, 55.6, 36.7, 34.3, 32.2, 31.4.
HRMS (ESI-TOF): *m/z* calculated for $C_{21}H_{28}NO_2$ $[M+H]^+$ = 326.2115; found 326.2120.

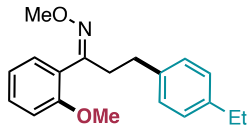

(*Z*)-3-(4-ethylphenyl)-1-(2-methoxyphenyl)propan-1-one *O*-methyl oxime (2z)
Prepared according to the general procedure **A**, except that MeOH was used as a solvent instead of HFIP in the first step. The title compound was isolated via flash chromatography (gradient 0–20% EtOAc/hexanes) (24 mg, 39% yield).
TLC (SiO_2) R_f = 0.35 4:1 hexanes/EtOAc
^{1}H NMR (600 MHz, $CDCl_3$) (δ, ppm): 7.34 – 7.30 (m, 1H), 7.09 (s, 5H), 6.98 (td, *J* = 7.4, 1.0 Hz, 1H), 6.94 (d, *J* = 8.3 Hz, 1H), 3.82 (s, 3H), 3.81 (s, 3H), 2.81 – 2.73 (m, 4H), 2.60 (q, *J* = 7.6 Hz, 2H), 1.21 (t, *J* = 7.6 Hz, 3H).
^{13}C NMR (151 MHz, $CDCl_3$) (δ, ppm): 156.8, 155.8, 141.9, 138.8, 129.9, 129.0, 129.0, 128.4, 128.4, 127.9, 127.92, 124.1, 120.5, 111.3, 61.8, 55.7, 37.0, 32.5, 28.6, 15.8.
HRMS (ESI-TOF): *m/z* calculated for $C_{19}H_{24}NO_2$ $[M+H]^+$ = 298.1802; found 298.1822.

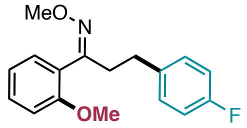

(*Z*)-3-(4-fluorophenyl)-1-(2-methoxyphenyl)propan-1-one *O*-methyl oxime (2aa)
Prepared according to the general procedure **A**, except that MeOH was used as a solvent instead of HFIP in the first step. The title compound was isolated via flash chromatography (gradient 0–20% EtOAc/hexanes) (22 mg, 38% yield).
TLC (SiO_2) R_f = 0.35 4:1 hexanes/EtOAc
^{1}H NMR (500 MHz, $CDCl_3$) (δ, ppm): 7.33 (td, *J* = 8.4, 7.9, 1.8 Hz, 1H), 7.11 (dd, *J* = 8.4, 5.5 Hz, 2H), 7.06 (dd, *J* = 7.5, 1.8 Hz, 1H), 7.01 – 6.96 (m, 1H), 6.93 (t, *J* = 8.6 Hz, 3H), 3.81 (s, 6H), 2.77 (s, 4H).
^{13}C NMR (126 MHz, $CDCl_3$) (δ, ppm): 161.4 (d, *J* = 243.3 Hz), 156.3, 155.8, 137.2 (d, *J* = 3.2 Hz), 130.0 (d, *J* = 7.3 Hz), 129.9, 129.0, 123.9, 120.5, 115.1 (d, *J* = 21.1 Hz), 111.3, 61.8, 55.8, 36.9, 32.0.
^{9}F NMR (471 MHz, $CDCl_3$) (δ, ppm): -117.72.
HRMS (ESI-TOF): *m/z* calculated for $C_{17}H_{19}FNO_2$ $[M+H]^+$ = 288.1394; found 288.1400.

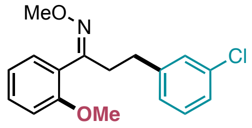

(*Z*)-3-(3-chlorophenyl)-1-(2-methoxyphenyl)propan-1-one *O*-methyl oxime (2ab)
Prepared according to the general procedure **A**, except that MeOH was used as a solvent instead of HFIP in the first step. The title compound was isolated via flash chromatography (gradient 0–20% EtOAc/hexanes) (21 mg, 34% yield).
TLC (SiO_2) R_f = 0.34 4:1 hexanes/EtOAc
^{1}H NMR (600 MHz, $CDCl_3$) (δ, ppm): 7.32 (dddd, *J* = 8.4, 7.4, 1.8, 1.0 Hz, 1H), 7.21 – 7.12 (m, 3H), 7.06 (tt, *J* = 6.4, 1.5 Hz, 2H), 6.98 (tt, *J* = 7.4, 1.0 Hz, 1H), 6.94 (dd, *J* = 8.3, 1.1 Hz, 1H), 3.81 (t, *J* = 1.1 Hz, 6H), 2.78 (d, *J* = 1.3 Hz, 4H).

^{13}C NMR (151 MHz, $CDCl_3$) (δ, ppm): 156.1, 155.8, 143.7, 134.1, 130.0, 129.6, 129.0, 128.8, 126.8, 126.2, 123.8, 120.5, 111.3, 61.8, 55.7, 36.5, 32.5.
HRMS (ESI-TOF): *m/z* calculated for $C_{17}H_{19}ClNO_2$ $[M+H]^+$ = 304.1099; found 304.1104.

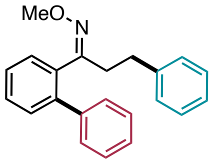

(*Z*)-1-([1,1'-biphenyl]-2-yl)-3-phenylpropan-1-one *O*-methyl oxime (2ac)
Prepared according to general procedure **B**. The title compound was isolated via flash chromatography (gradient 0 –20% EtOAc/hexanes) (22% yield).
TLC (SiO_2) R_f = 0.22 in 4:1 hexanes/EtOAc
^{1}H NMR (500 MHz, $CDCl_3$) (δ, ppm): 7.45 – 7.33 (m, 8H), 7.23 – 7.16 (m, 3H), 7.16 – 7.10 (m, 1H), 7.00 – 6.94 (m, 2H), 3.85 (s, 3H), 2.58 (t, *J* = 8.4 Hz, 2H), 2.43 – 2.14 (m, 2H).
^{13}C{H} NMR (126 MHz, $CDCl_3$) (δ, ppm): 159.3, 141.3, 140.6, 139.3, 134.1, 129.9, 128.7, 128.7, 128.6, 128.5, 128.4, 128.4, 127.7, 127.1, 126.0, 61.7, 36.9, 32.8.
HRMS (ESI-TOF): *m/z* calculated for $C_{22}H_{22}NO$ $[M+H]^+$ = 316.1701; found 316.1693.

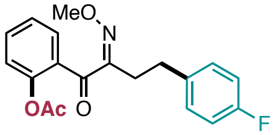

(Z)-2-(4-(4-fluorophenyl)-2-(methoxyimino)butanoyl)phenyl acetate (10)
Following General Procedure **N**. The title compound was isolated via flash chromatography (Hex:DCM:Et_2O 7:2:1 to 5:3:2) (9.9 mg, 44 % yield).
TLC (SiO_2) R_f = 0.19 in Hex: EtOAc = 9:1.
^{1}H NMR (500 MHz, $CDCl_3$) (δ, ppm): 7.73 (dd, *J* = 7.8, 1.7 Hz, 1H), 7.63 – 7.59 (m, 1H), 7.34 (td, *J* = 7.6, 1.1 Hz, 1H), 7.15 (ddd, *J* = 8.5, 3.5, 2.4 Hz, 3H), 6.95 (t, *J* = 8.7 Hz, 2H), 3.74 (s, 3H), 2.90 – 2.86 (m, 2H), 2.71 – 2.67 (m, 2H), 2.36 (s, 3H).
^{13}C NMR (126 MHz, $CDCl_3$) (δ, ppm) 192.4, 169.1, 162.5, 160.5 (d, *J* = 244.0 Hz), 157.1, 150.2, 136.1 (d, *J* = 3.2 Hz), 135.0, 131.9, 129.9 (d, *J* = 7.8 Hz), 126.7, 126.3, 124.1, 115.2 (d, *J* = 21.2 Hz), 62.1, 33.0, 31.2, 29.7, 21.0.
^{9}F NMR (471 MHz, $CDCl_3$) (δ, ppm): -117.1.
HRMS (ESI-TOF): *m/z* calculated for $C_{19}H_{19}FNO_4$ $[M+H]^+$ = 344.1289; found 344.1298.

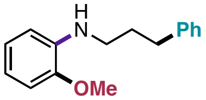

2-Methoxy-*N*-(3-phenylpropyl)aniline (11)
Prepared according to general procedure **H**. The title compound was isolated via flash chromatography (gradient 0–20% EtOAc/hexanes) (82 mg, 91% yield).
TLC (SiO_2) R_f = 0.38 (1:9 EtOAc/Hex)
^{1}H NMR (600 MHz, $CDCl_3$) (δ, ppm): 7.22 – 7.14 (m, 4H), 6.90 (td, *J* = 7.4, 1.1 Hz, 1H), 6.86 (dd, *J* = 8.2, 1.1 Hz, 1H), 6.68 (tt, *J* = 7.3, 1.1 Hz, 1H), 6.61 – 6.58 (m, 2H), 3.83 (s, 3H), 3.14 (t, *J* = 6.9 Hz, 2H), 2.73 (d, *J* = 7.5 Hz, 2H), 1.92 (dq, *J* = 8.6, 6.9 Hz, 2H).
^{13}C NMR (151 MHz, $CDCl_3$) (δ, ppm): 157.6, 148.7, 130.2, 130.0, 129.3, 127.3, 120.6, 117.2, 112.9, 110.4, 55.4, 43.6, 29.6, 27.8.
HRMS (ESI-TOF): *m/z* calculated for $C_{16}H_{20}NO$ $[M+H]^+$ = 242.1545; found 242.1425.

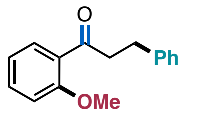

1-(2-methoxyphenyl)-3-phenylpropan-1-one (12)
Prepared according to general procedure **I**. The title compound was isolated via flash chromatography (gradient 0–20% EtOAc/hexanes) (7.0 mg, 29% yield).
TLC (SiO_2) R_f = 0.40 (10% EA/Hex)
^{1}H NMR (400 MHz, $CDCl_3$) (δ, ppm): 7.99 (dt, *J* = 8.3, 1.1 Hz, 2H), 7.61 – 7.50 (m, 1H), 7.50 – 7.42 (m, 2H), 7.22 (ddd, *J* = 7.1, 4.4, 2.6 Hz, 2H), 6.94 – 6.84 (m, 2H), 3.84 (s, 3H), 3.33 – 3.25 (m, 2H), 3.07 (dd, *J* = 8.9, 6.6 Hz, 2H).
^{13}C NMR (101 MHz, $CDCl_3$) (δ, ppm): 200.1, 157.6, 137.1, 133.0, 130.3, 129.6, 128.6, 128.2, 127.6, 120.6, 110.3, 55.3, 39.0, 25.8.

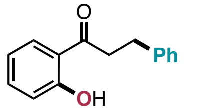

1-(2-hydroxyphenyl)-3-phenylpropan-1-one (14)
Prepared according to general procedure **J**. The title compound was isolated via flash chromatography (gradient 0–20% EtOAc/hexanes) (7.1 mg, 93% yield).
TLC (SiO_2) R_f = 0.46 (10% EA/Hex)
^{1}H NMR (400 MHz, $CDCl_3$) (δ, ppm): 12.30 (s, 1H), 7.75 (dd, *J* = 8.0, 1.6 Hz, 1H), 7.47 (ddd, *J* = 8.7, 7.2, 1.7 Hz, 1H), 7.34 – 7.28 (m, 2H), 7.26 – 7.20 (m, 3H), 6.99 (dd, *J* = 8.5, 1.2 Hz, 1H), 6.88 (ddd, *J* = 8.2, 7.2, 1.2 Hz, 1H), 3.34 (dd, *J* = 8.5, 6.9 Hz, 2H), 3.08 (dd, *J* = 8.4, 6.9 Hz, 2H).
^{13}C NMR (101 MHz, $CDCl_3$) (δ, ppm): 205.5, 162.6, 140.9, 136.5, 130.0, 128.8, 128.5, 126.5, 119.4, 119.1, 118.7, 40.2, 30.2.

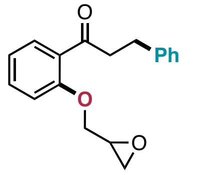

1-(2-(oxiran-2-ylmethoxy)phenyl)-3-phenylpropan-1-one
Prepared according to general procedure **K**. The title compound was isolated via flash chromatography (gradient 0–20% EtOAc/hexanes) (120 mg, 49% yield).
TLC (SiO_2) R_f = 0.35 (20% EA/Hex)
^{1}H NMR (400 MHz, $CDCl_3$) (δ, ppm): 7.66 (dd, *J* = 7.7, 1.9 Hz, 1H), 7.43 (ddd, *J* = 8.4, 7.3, 1.9 Hz, 1H), 7.31 – 7.22 (m, 4H), 7.21 – 7.15 (m, 1H), 7.03 (td, *J* = 7.5, 1.0 Hz, 1H), 6.94 (dd, *J* = 8.4, 1.0 Hz, 1H), 4.31 (dd, *J* = 11.0, 3.1 Hz, 1H), 4.02 (dd, *J* = 11.0, 5.7 Hz, 1H), 3.40 – 3.30 (m, 3H), 3.05 (t, *J* = 7.7 Hz, 2H), 2.90 – 2.85 (m, 1H), 2.71 (dd, *J* = 4.9, 2.6 Hz, 1H).
^{13}C NMR (101 MHz, $CDCl_3$) (δ, ppm): 201.9, 157.3, 141.7, 133.4, 130.5, 129.0, 128.5, 128.5, 126.0, 121.5, 112.8, 69.6, 50.0, 45.4, 44.7, 30.4.

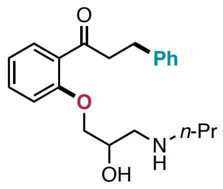

Propafenone (15)
Prepared according to general procedure **L**. The title compound was isolated via flash chromatography (gradient 0–20% EtOAc/hexanes) (12.5 mg, 50% yield).
TLC (SiO_2) R_f=0.24 (2% MeOH/DCM)
^{1}H NMR (600 MHz, $CDCl_3$) (δ, ppm): 7.69 (dd, J = 7.7, 1.8 Hz, 1H), 7.46 (ddd, J = 8.8, 7.3, 1.8 Hz, 1H), 7.28 (t, J = 7.6 Hz, 2H), 7.24 – 7.21 (m, 2H), 7.20 – 7.16 (m, 1H), 7.02 (td, J = 7.6, 0.9 Hz, 1H), 6.99 – 6.95 (m, 1H), 4.29 (dt, J = 8.8, 4.6 Hz, 1H), 4.15 – 4.09 (m, 2H), 3.38 – 3.24 (m, 2H), 3.10 (dd, J = 12.4, 4.2 Hz, 1H), 3.02 (t, J = 7.7 Hz, 2H), 2.93 (dd, J = 12.3, 6.5 Hz, 1H), 2.74 (ddt, J = 23.0, 12.0, 6.1 Hz, 2H), 1.73 (h, J = 7.4 Hz, 2H), 0.95 (t, J = 7.4 Hz, 3H).
^{13}C NMR (151 MHz, $CDCl_3$) (δ, ppm): 201.7, 157.6, 141.4, 134.1, 130.7, 128.7, 128.6, 128.5, 127.7, 126.2, 121.3, 113.3, 71.57, 66.3, 51.8, 51.2, 44.5, 30.4, 21.3, 11.5.

Characterization Data of Difunctionalized Products (^{1}H NMR Yield)

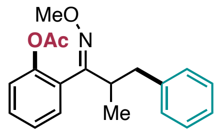

(*Z*)-2-(1-(methoxyimino)-2-methyl-3-phenylpropyl)phenyl acetate (2l)

Following General Procedure **A**, prepared from 1.0 equiv. of **1l**, the title compound is reported as 38 % ^{1}H NMR yield (1.0 equiv. of CH_2Br_2 was used as an internal standard).

^{1}H NMR (Crude) (500 MHz, $CDCl_3$) (δ, ppm): 3.47 (tq, J = 13.2, 6.6 Hz, 1H).

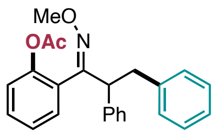

(*Z*)-2-(1-(methoxyimino)-2,3-diphenylpropyl)phenyl acetate (2m)

Following General Procedure **A**, prepared from 1.0 equiv. of **1m**, the title compound is reported as 33 % ^{1}H NMR yield (1.0 equiv. of CH_2Br_2 was used as an internal standard).

^{1}H NMR (Crude) (500 MHz, $CDCl_3$) (δ, ppm): 4.69 (q, J = 6.9 Hz, 2H).

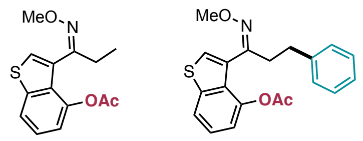

2o- P1 **2o- P2**

(*Z*)-3-(1-(methoxyimino)-3-phenylpropyl)benzo[*b*]thiophen-4-yl acetate (2o)

Following General Procedure **A**, prepared from 1.0 equiv. of **1o**, the title compound is reported as 9% ^{1}H NMR yield of the desired product along with 33% ^{1}H NMR yield of product after second step (1.0 equiv. of CH_2Br_2 was used as an internal standard).

^{1}H NMR (Crude) (500 MHz, $CDCl_3$) (δ, ppm): 2.05 (s, 3H) and 2.11 (s, 3H).

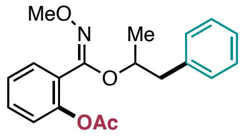

(*E*)-2-((methoxyimino)((1-phenylpropan-2-yl)oxy)methyl)phenyl acetate (2r)

Following General Procedure **A**, prepared from 1.0 equiv. of **1r**, the title compound is reported as 41 % ^{1}H NMR yield (1.0 equiv. of CH_2Br_2 was used as an internal standard).

^{1}H NMR (Crude) (500 MHz, $CDCl_3$) (δ, ppm): 4.15– 4.05 (m, 1H).

Spectral Data: ^{1}H and ^{13}C NMR Spectra

^{1}H NMR (400 MHz, $CDCl_3$) of (E)-1-(4-(tert-butyl)phenyl)propan-1-one O-methyl oxime (1c)

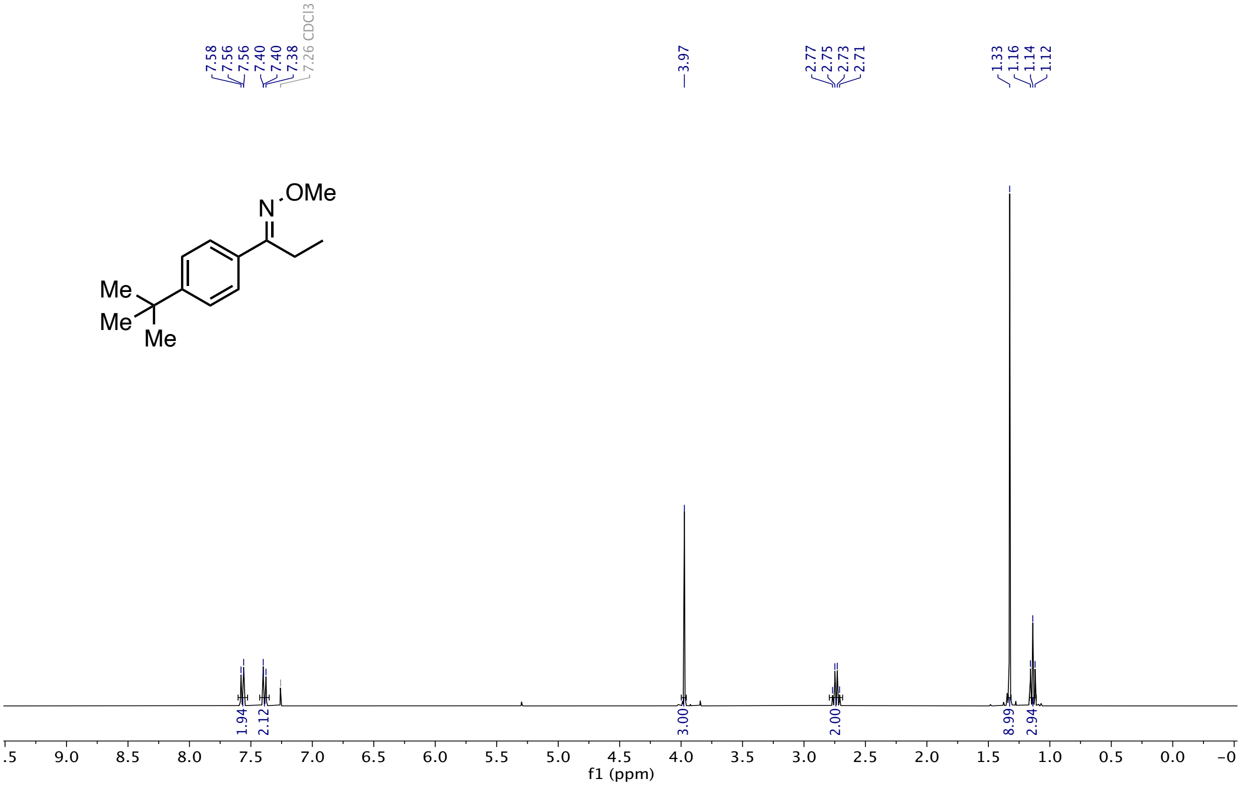

^{13}C NMR (101 MHz, $CDCl_3$) of (E)-1-(4-(tert-butyl)phenyl)propan-1-one O-methyl oxime (1c)

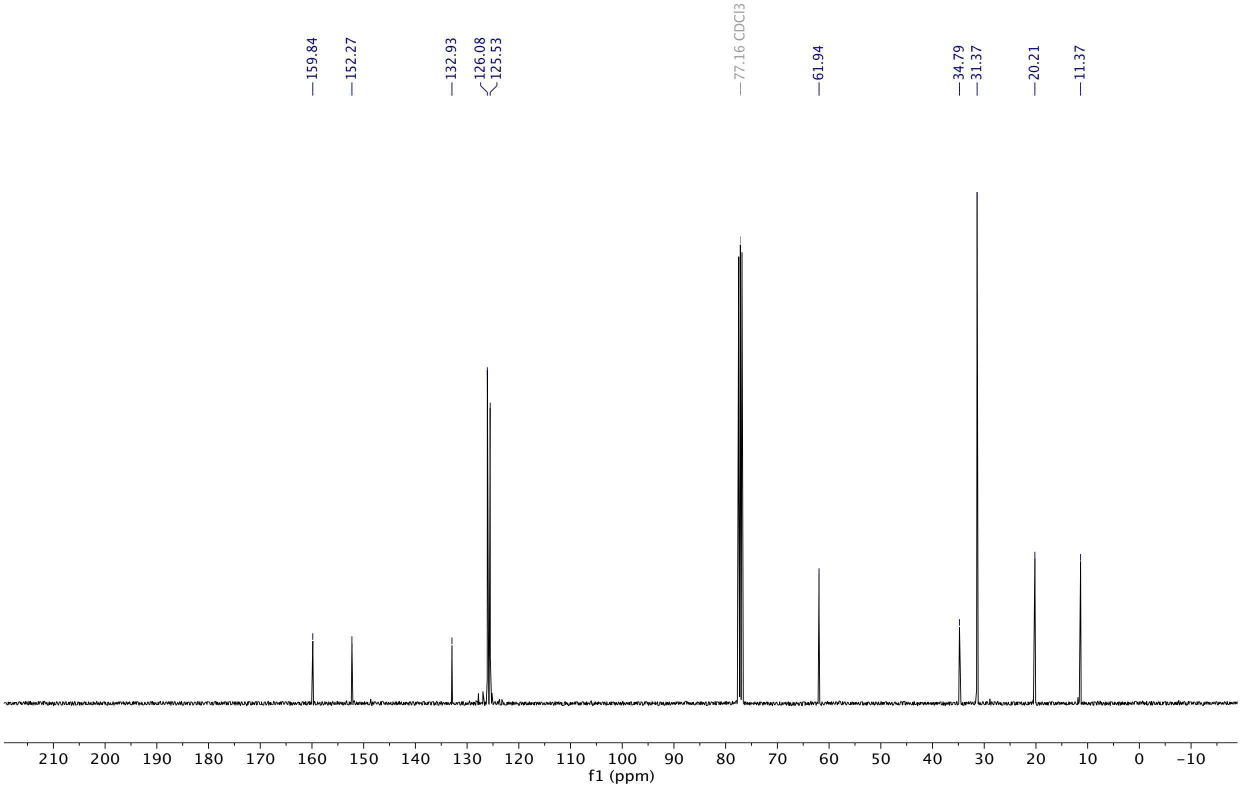

^{1}H NMR (400 MHz, $CDCl_3$) of (*E*)-1-(5,6,7,8-tetrahydronaphthalen-2-yl)propan-1-one *O*-methyl oxime (1i)

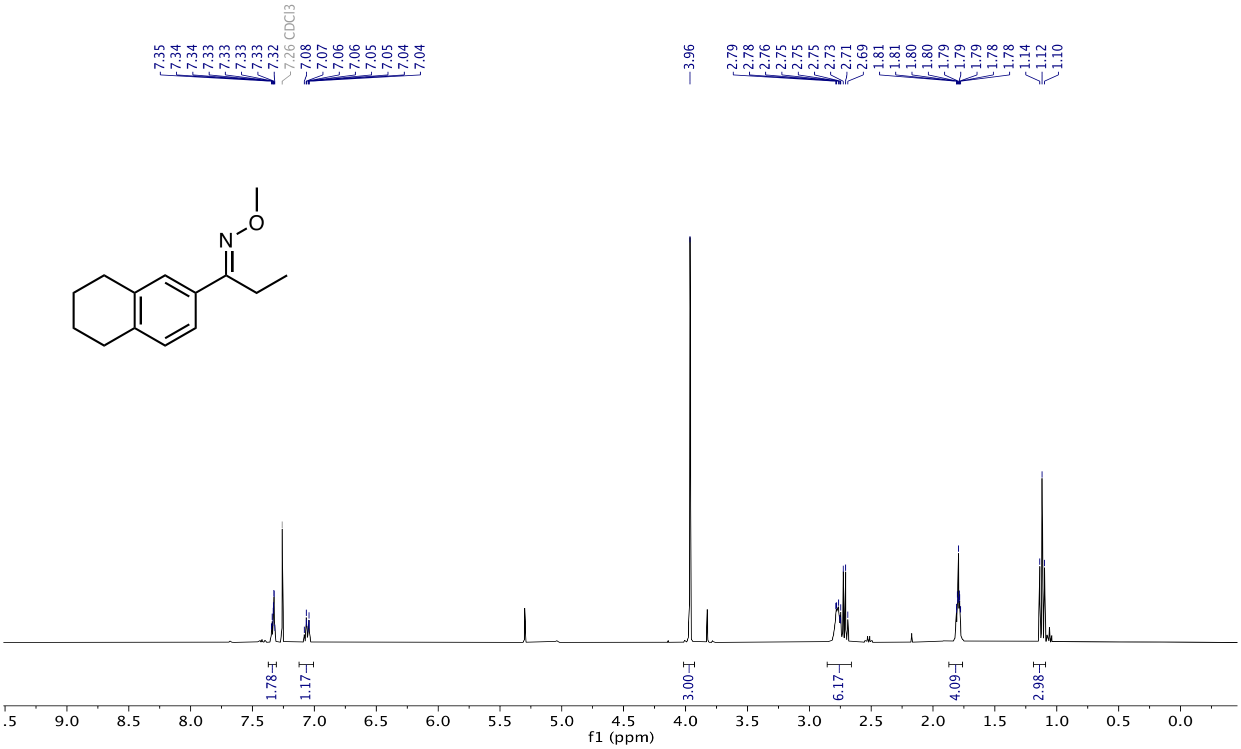

^{13}C{H} NMR (101 MHz, $CDCl_3$) of (*E*)-1-(5,6,7,8-tetrahydronaphthalen-2-yl)propan-1-one *O*-methyl oxime (1i)

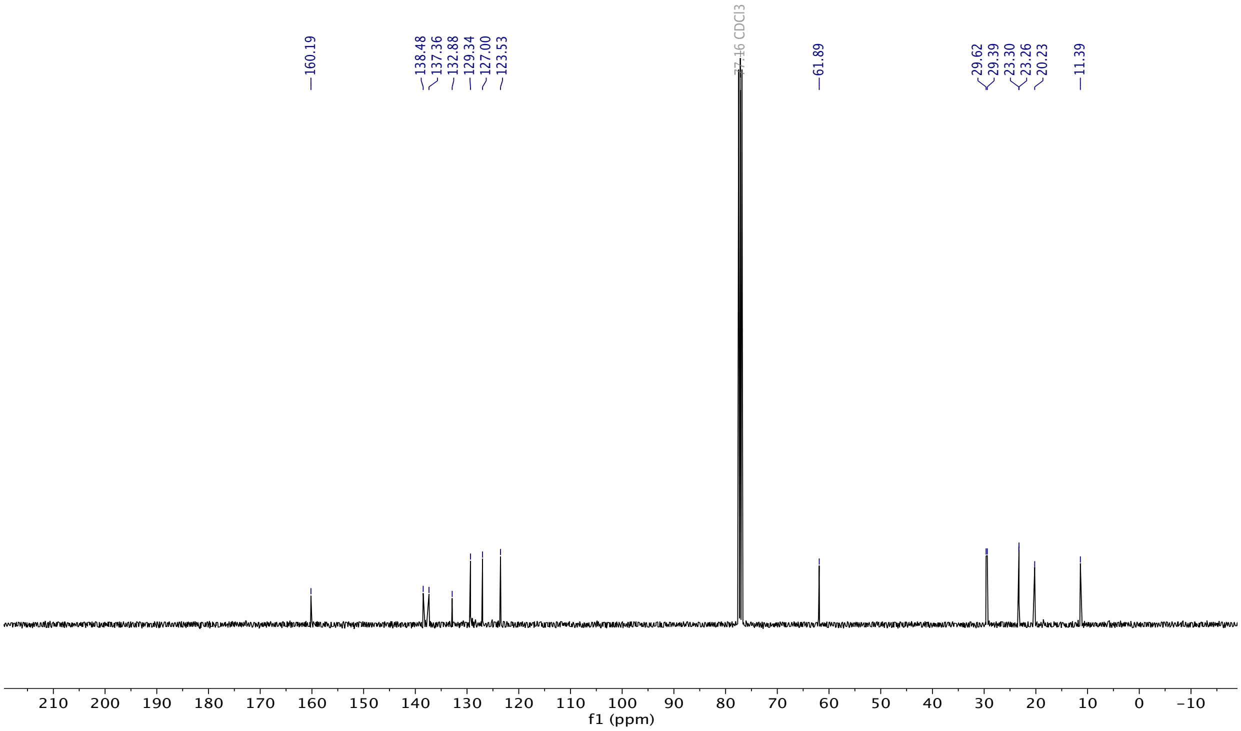

^{1}H NMR (400 MHz, $CDCl_3$) of (E)-1-(benzo[d][1,3]dioxol-5-yl)propan-1-one O-methyl oxime (1j)

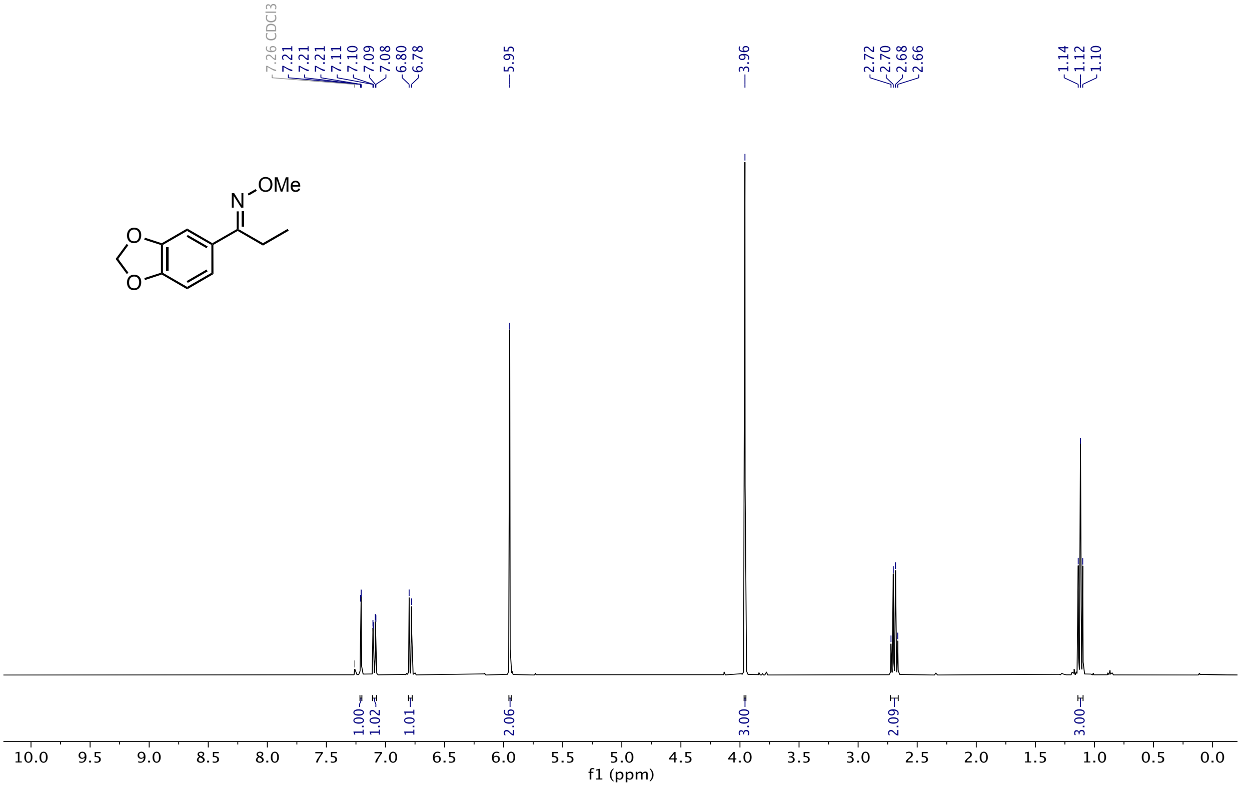

^{13}C NMR (101 MHz, $CDCl_3$) of (E)-1-(benzo[d][1,3]dioxol-5-yl)propan-1-one O-methyl oxime (1j)

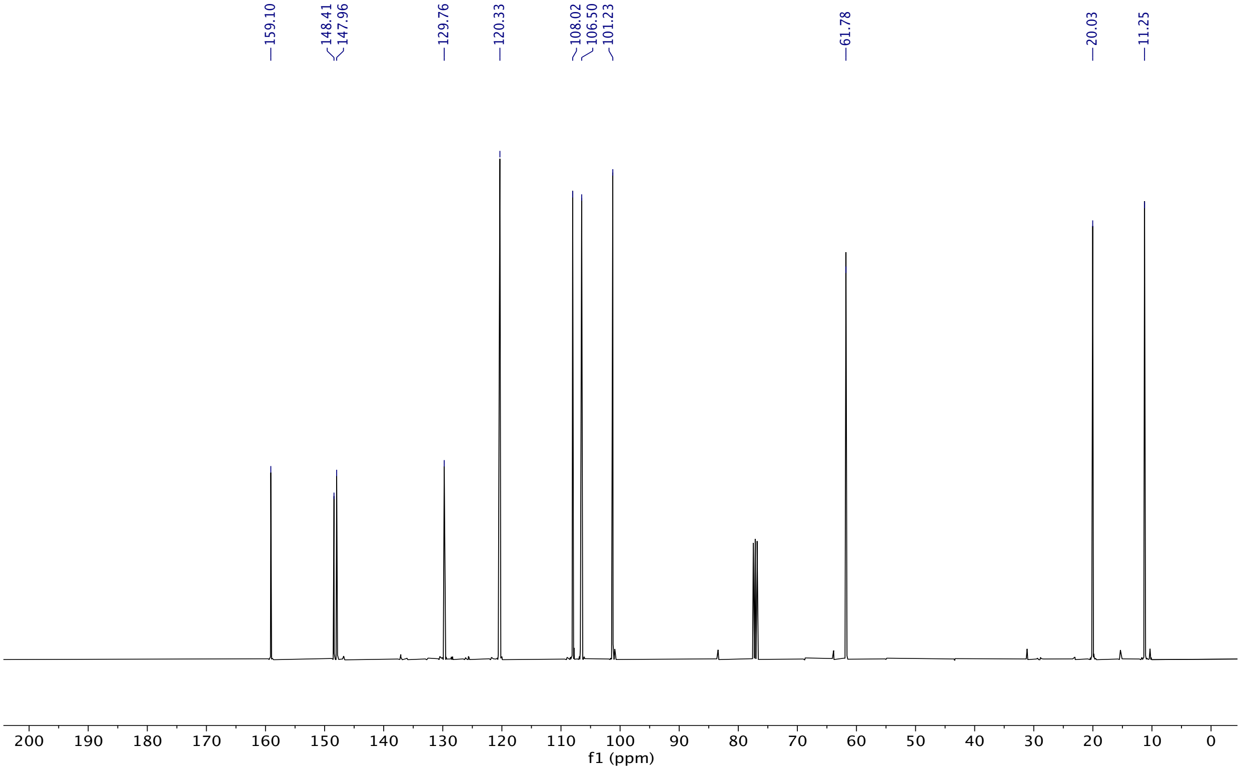

^{1}H NMR (400 MHz, $CDCl_3$) of (*Z*)-2-methyl-3,4-dihydronaphthalen-1(2*H*)-one *O*-methyl oxime (1n)

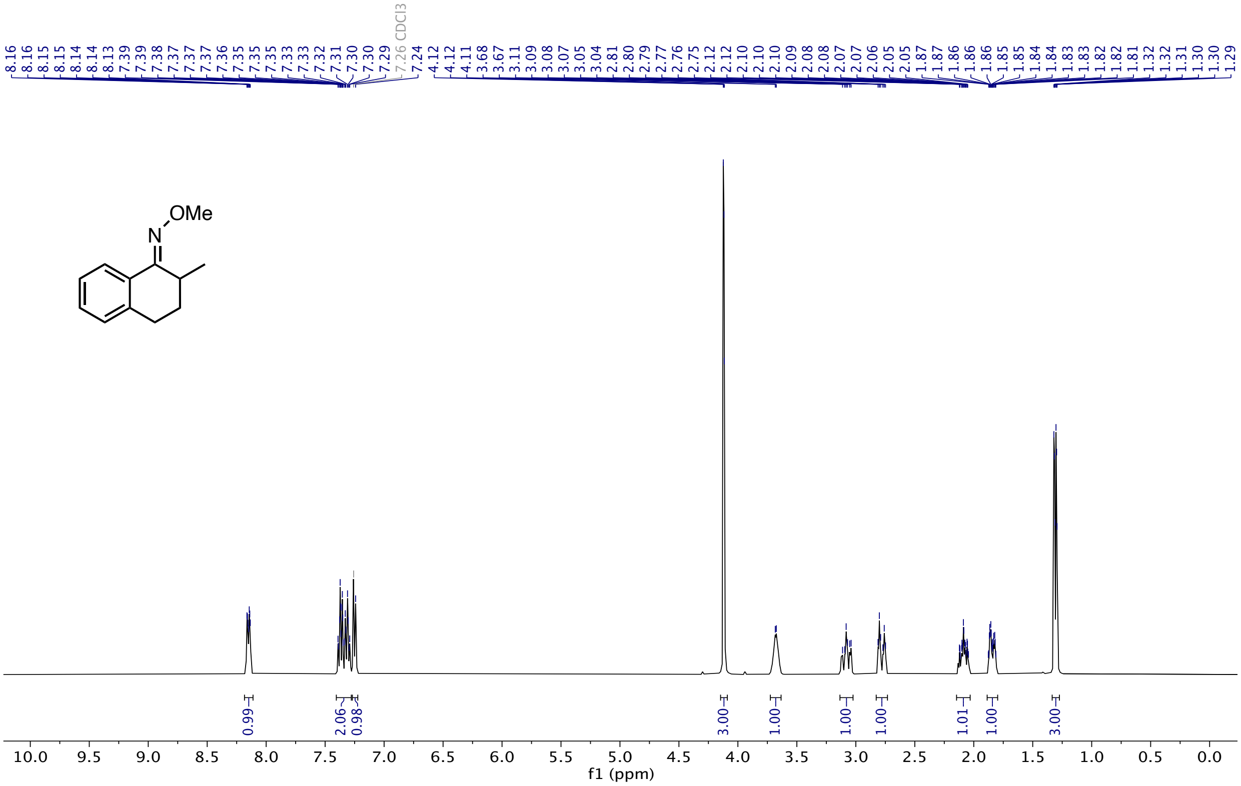

^{13}C NMR (101 MHz, $CDCl_3$) of (*Z*)-2-methyl-3,4-dihydronaphthalen-1(2*H*)-one *O*-methyl oxime (1n)

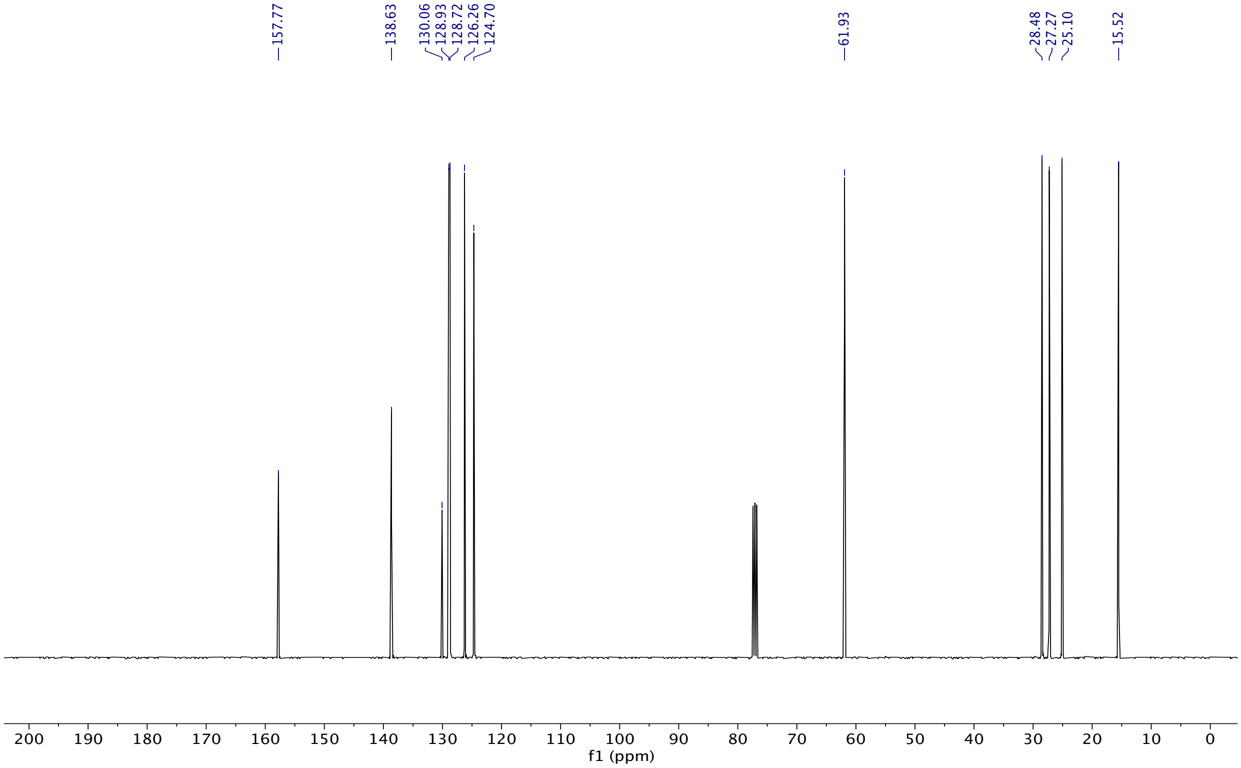

^{1}H NMR (400 MHz, CDCl$_3$) of (*E*)-1-(benzo[*b*]thiophen-3-yl)propan-1-one *O*-methyl oxime (1o)

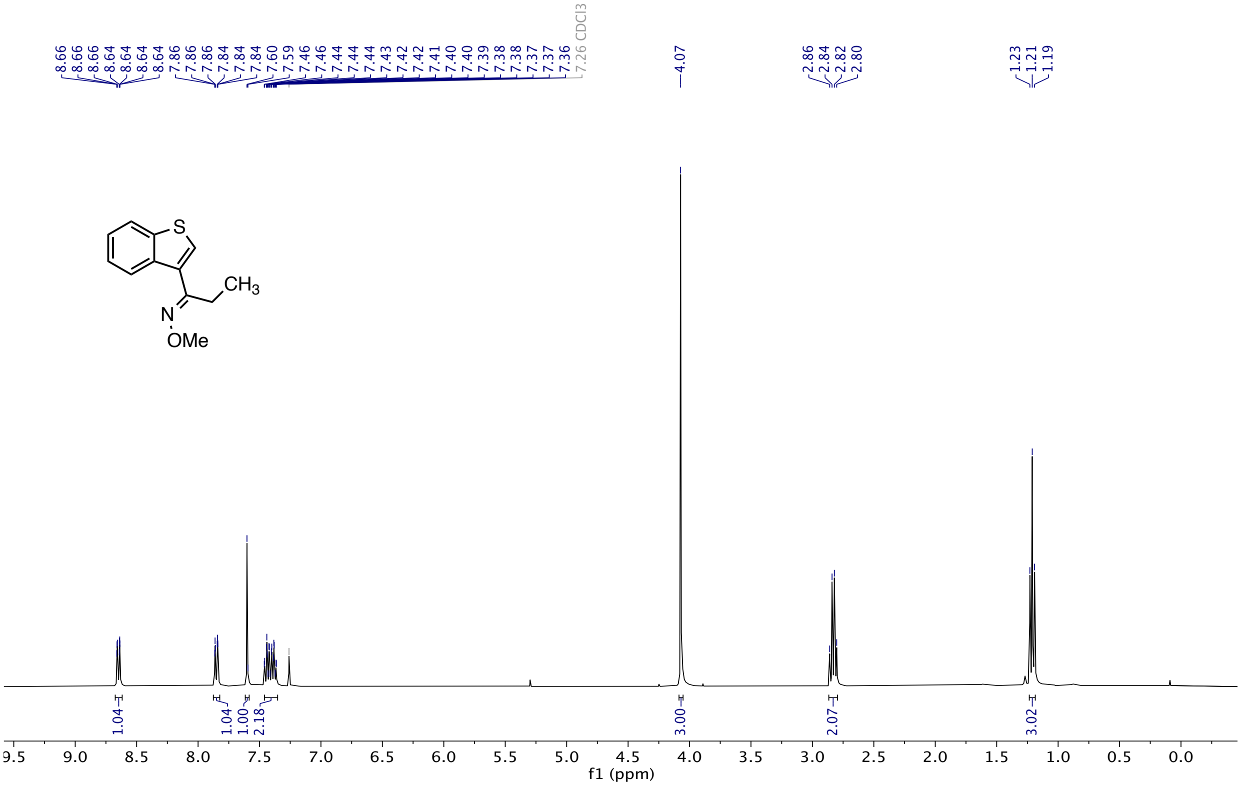

^{13}C NMR (101 MHz, CDCl$_3$) of (*E*)-1-(benzo[*b*]thiophen-3-yl)propan-1-one *O*-methyl oxime (1o)

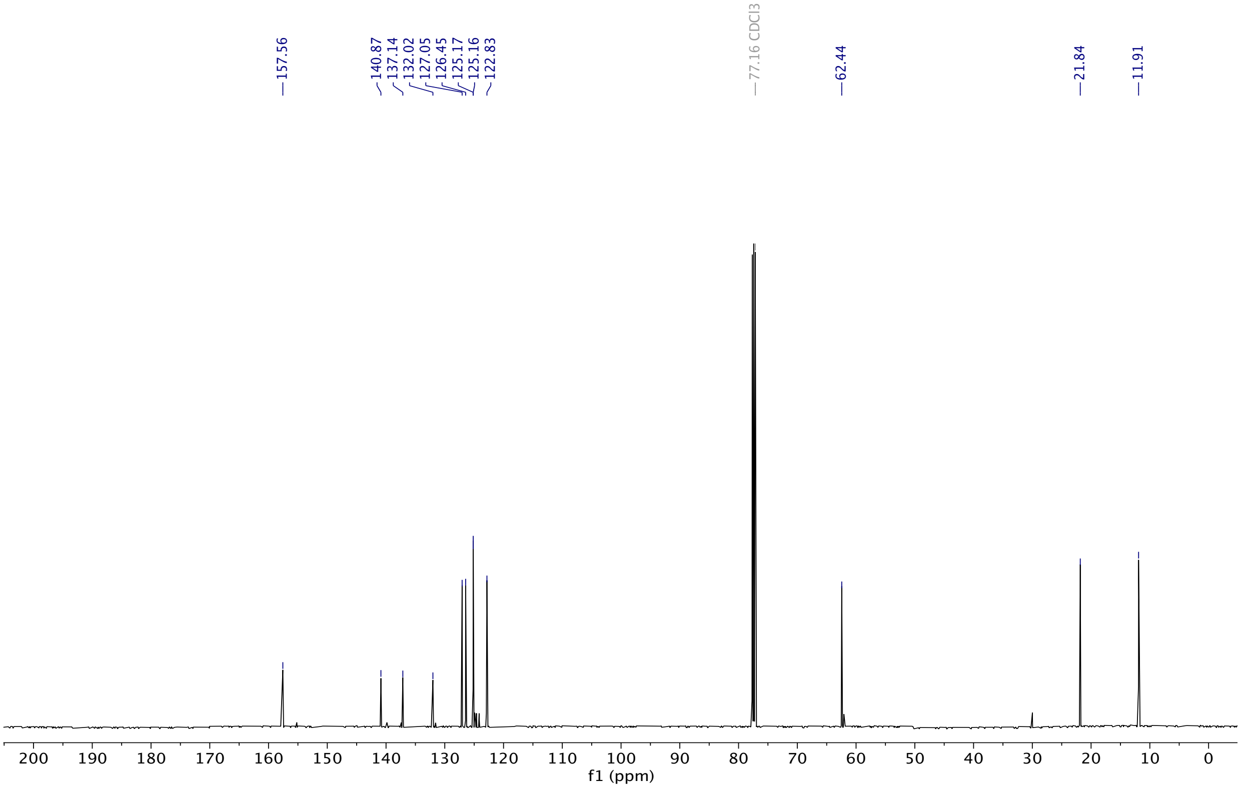

^{1}H NMR (500 MHz, $CDCl_3$) of (*E*)-2-(methoxyimino)-1-phenylbutan-1-one (1p)

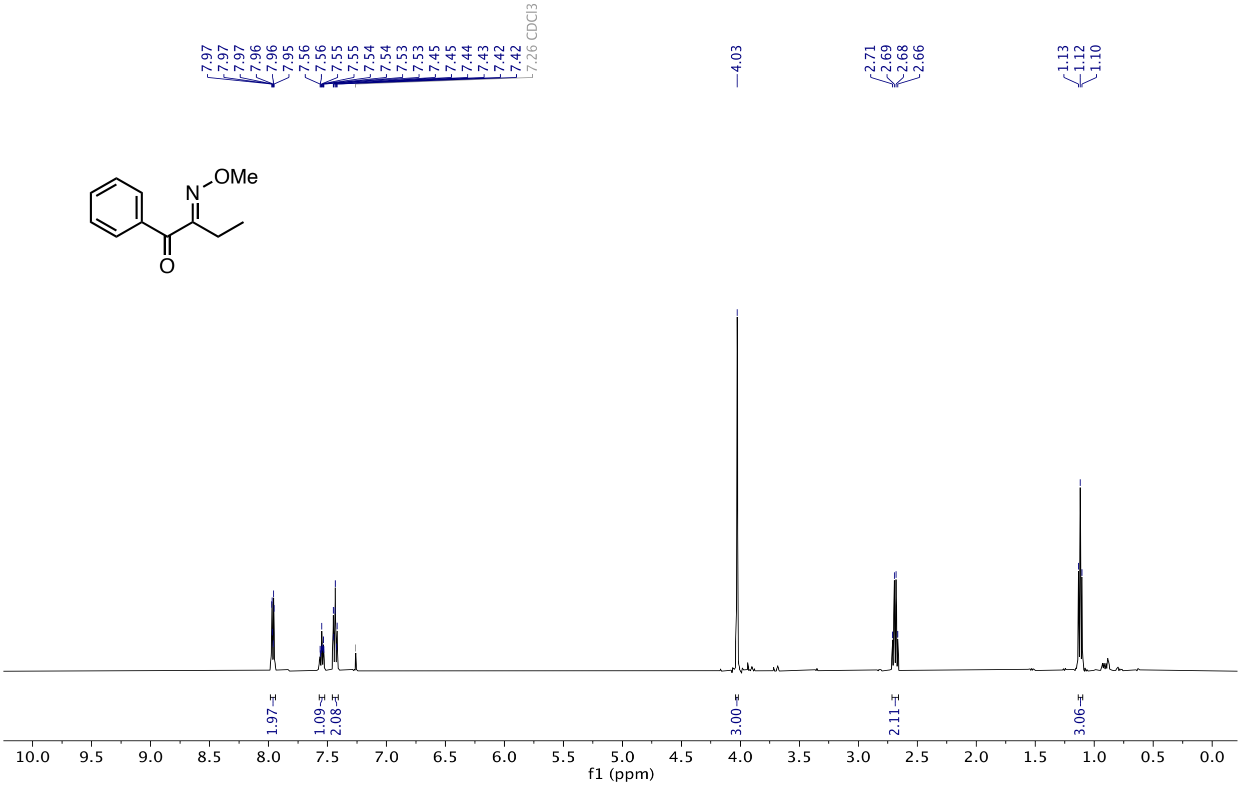

^{13}C NMR (101 MHz, $CDCl_3$) of (*E*)-2-(methoxyimino)-1-phenylbutan-1-one (1p)

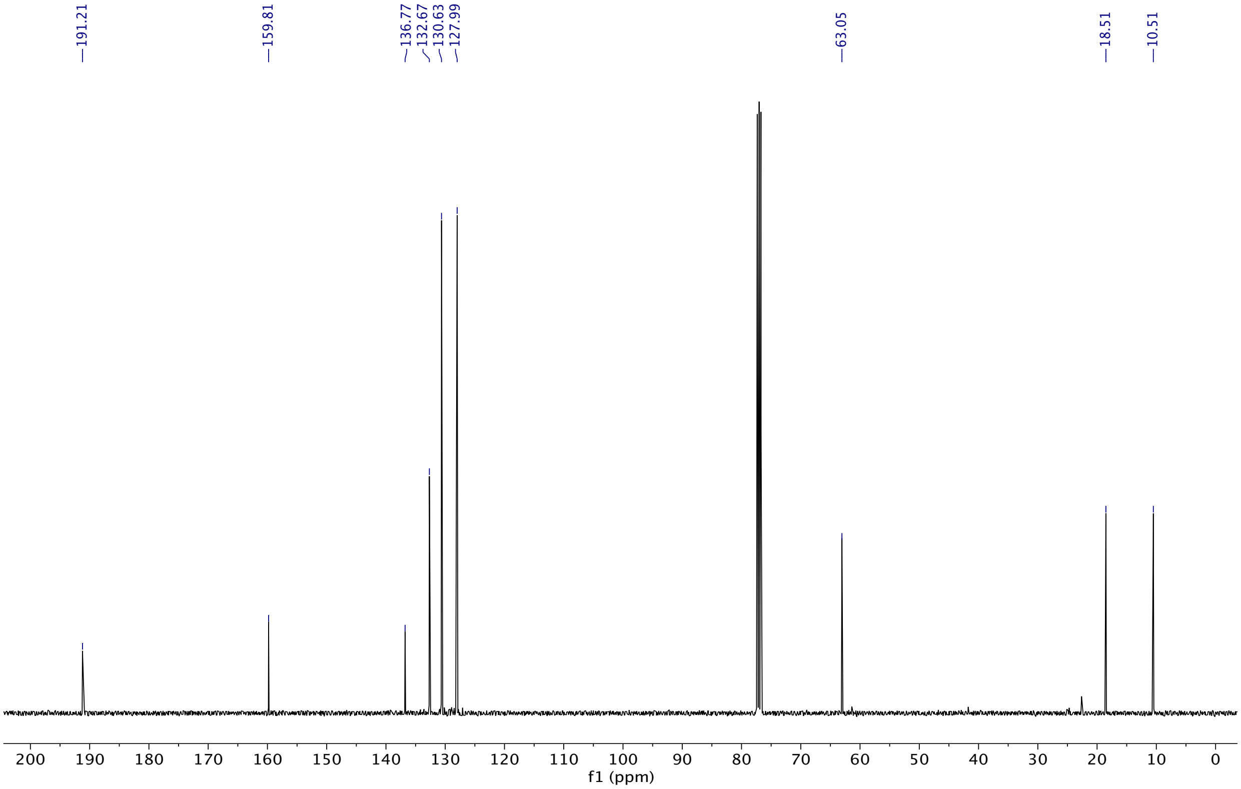

^{1}H NMR (500 MHz, $CDCl_3$) of Methyl (*E*)-2-(2-(methoxyimino)-2-phenylacetamido)butanoate (1q)

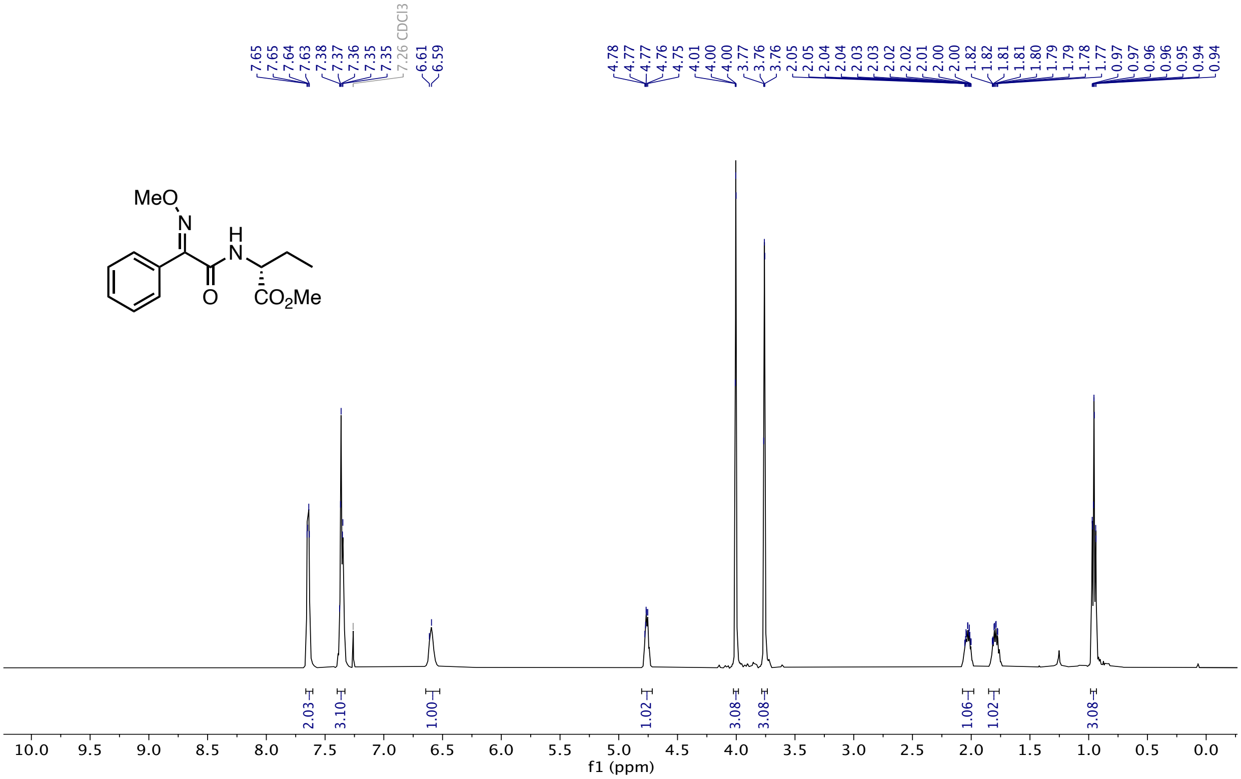

^{13}C NMR (101 MHz, $CDCl_3$) of Methyl (*E*)-2-(2-(methoxyimino)-2-phenylacetamido)butanoate (1q)

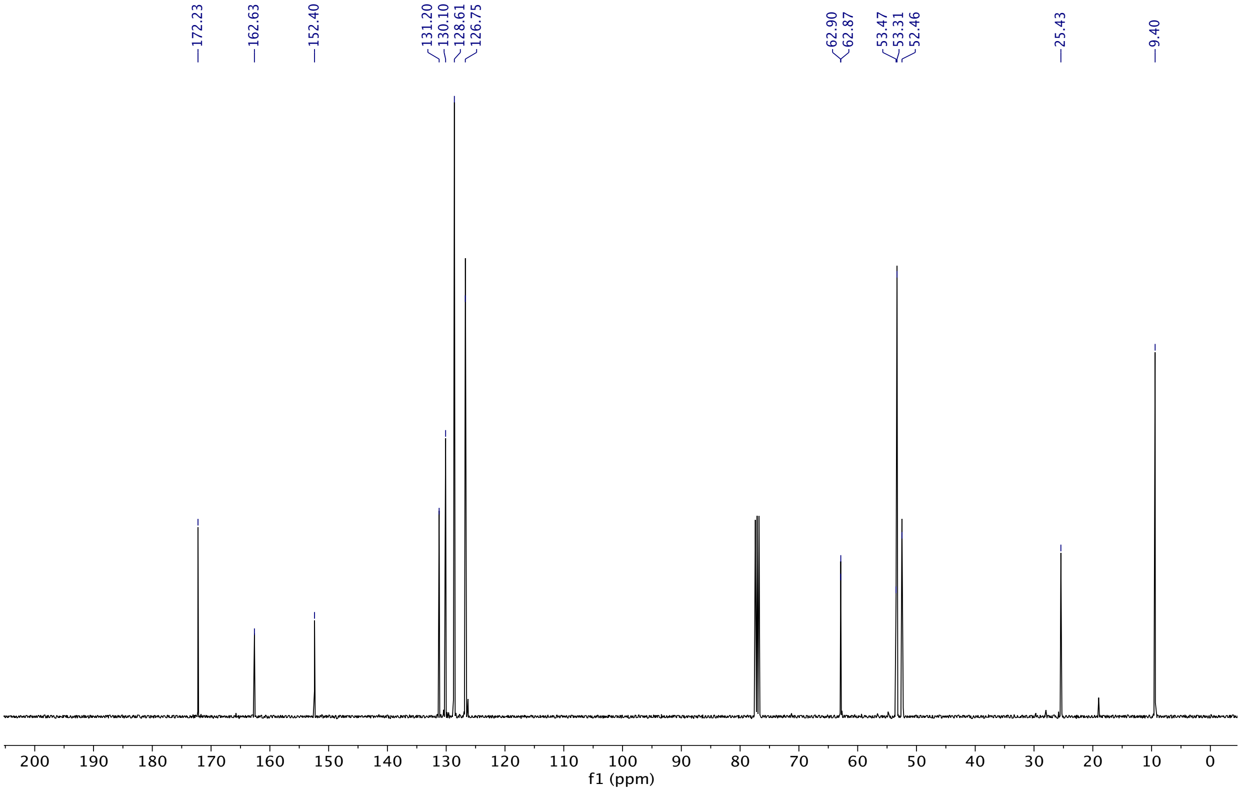

^{1}H NMR (500 MHz, $CDCl_3$) of Isopropyl (E)-N-methoxybenzimidate (1r)

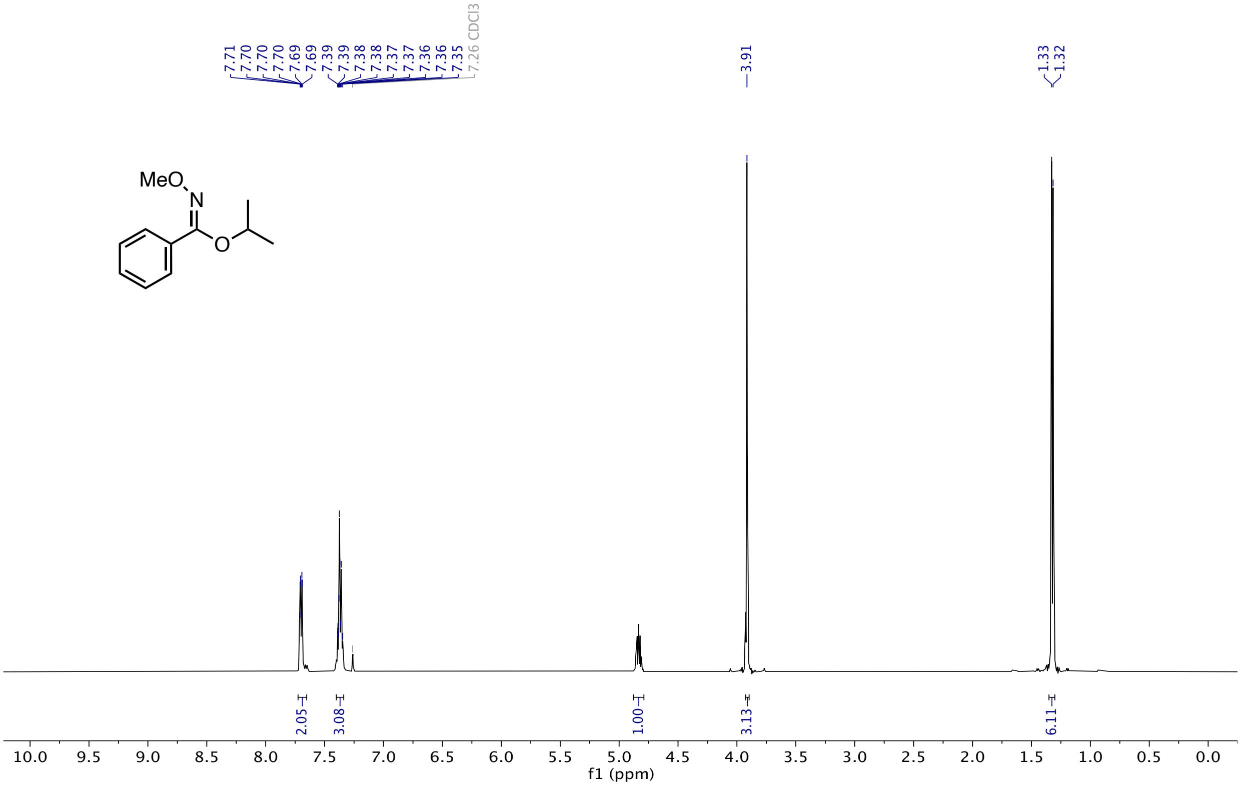

^{13}C NMR (101 MHz, $CDCl_3$) of Isopropyl (E)-N-methoxybenzimidate (1r)

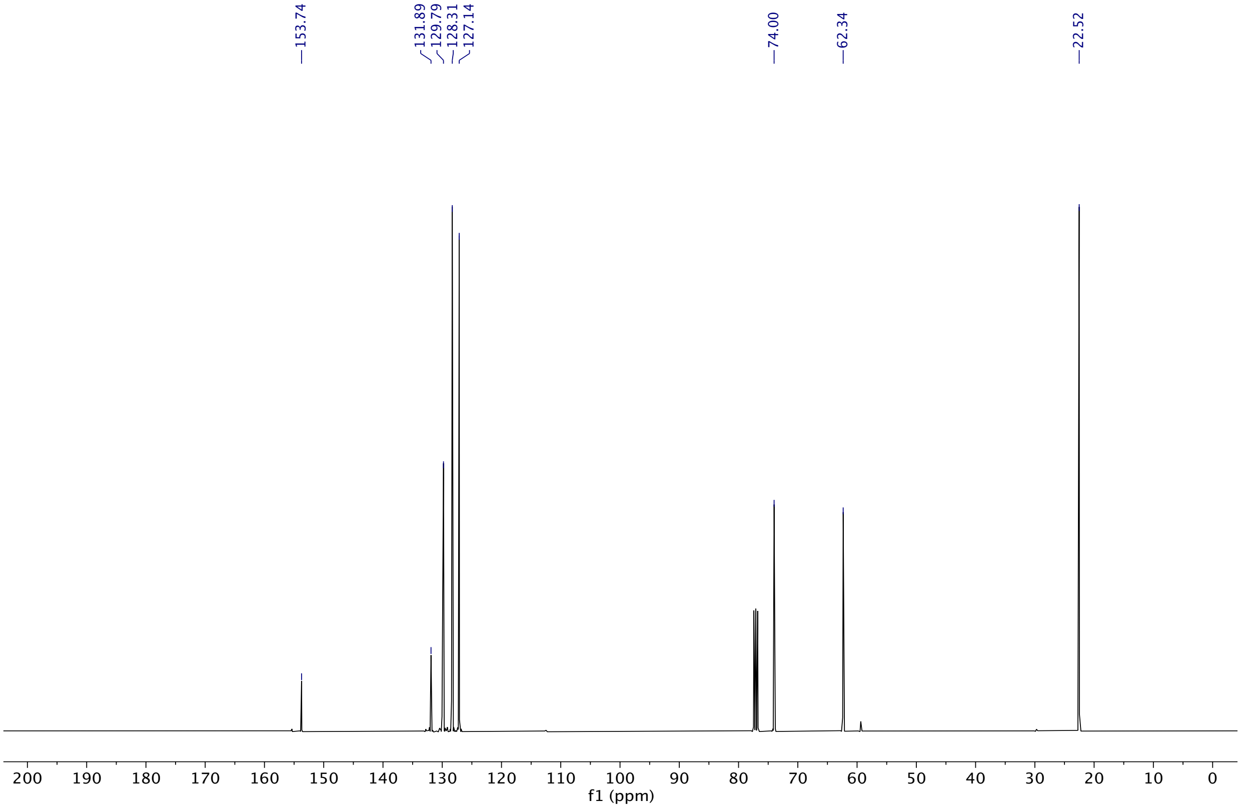

^{1}H NMR (500 MHz, CDCl$_3$) of (*Z*)-2-(1-(methoxyimino)-3-phenylpropyl)phenyl acetate (2a)

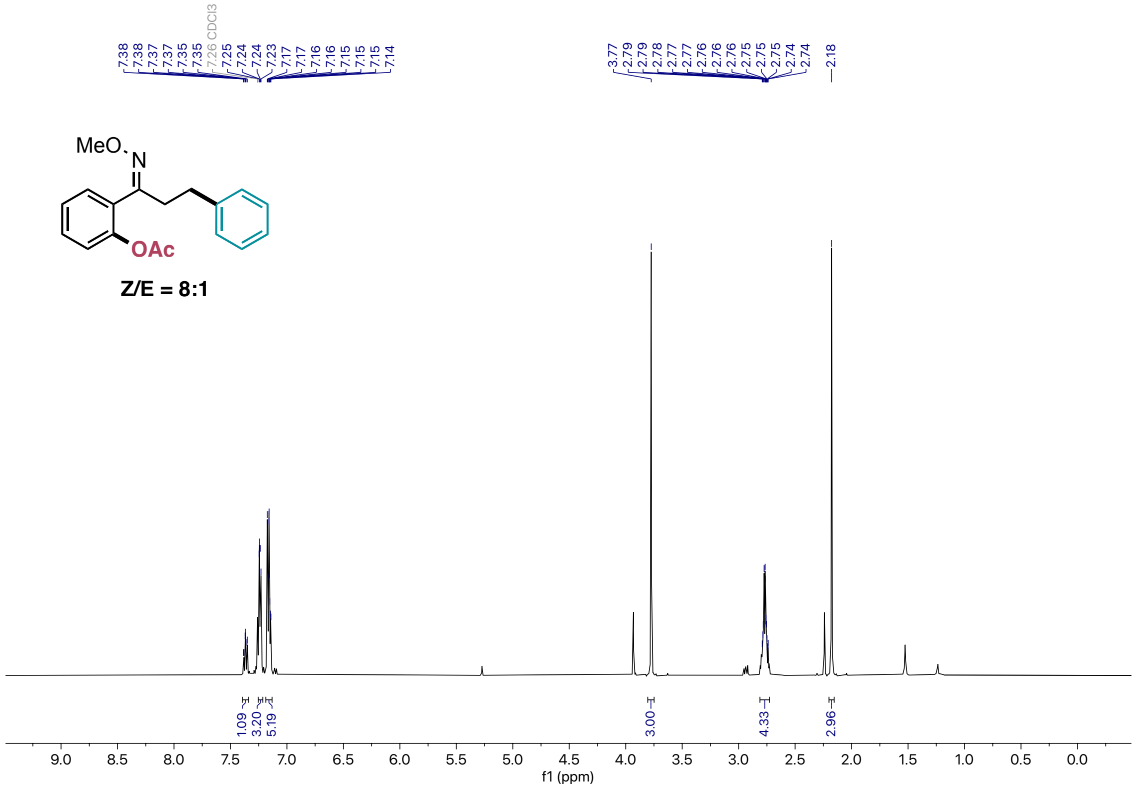

^{13}C{H} NMR (126 MHz, CDCl$_3$) of (*Z*)-2-(1-(methoxyimino)-3-phenylpropyl)phenyl acetate (2a)

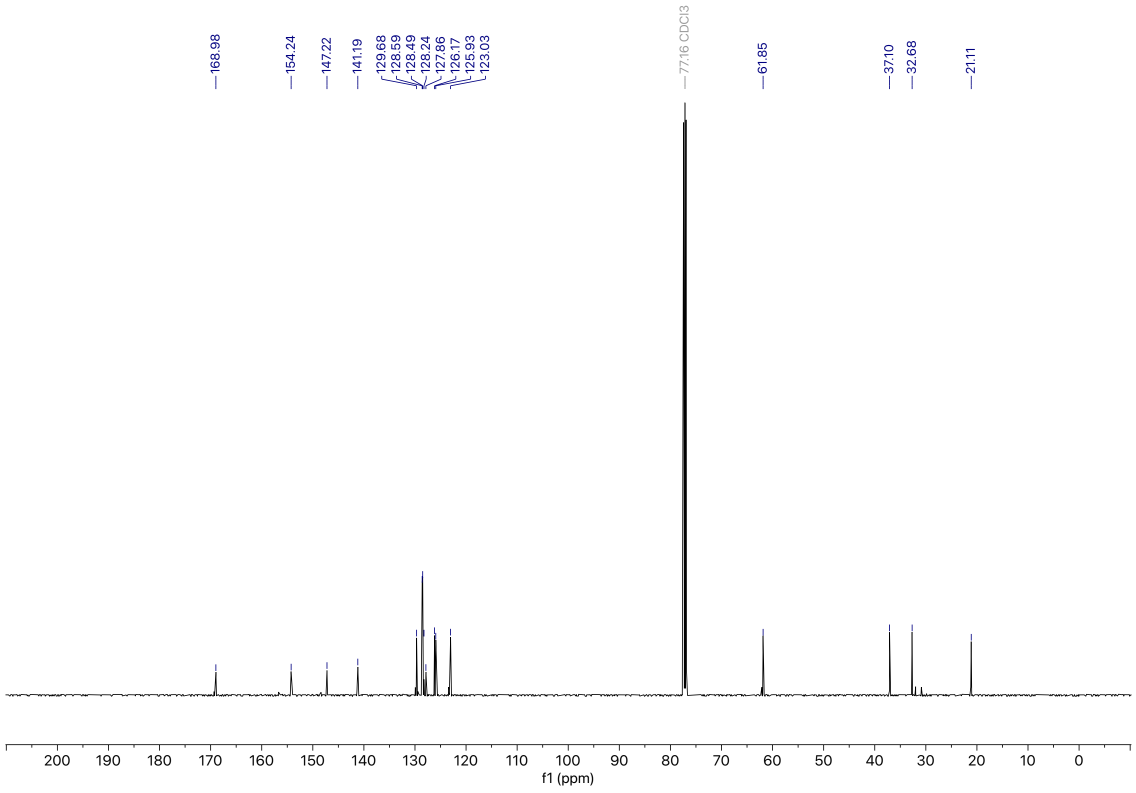

^{1}H NMR (400 MHz, $CDCl_3$) of (*Z*)-5-methoxy-2-(1-(methoxyimino)-3-phenylpropyl)phenyl acetate (2b)

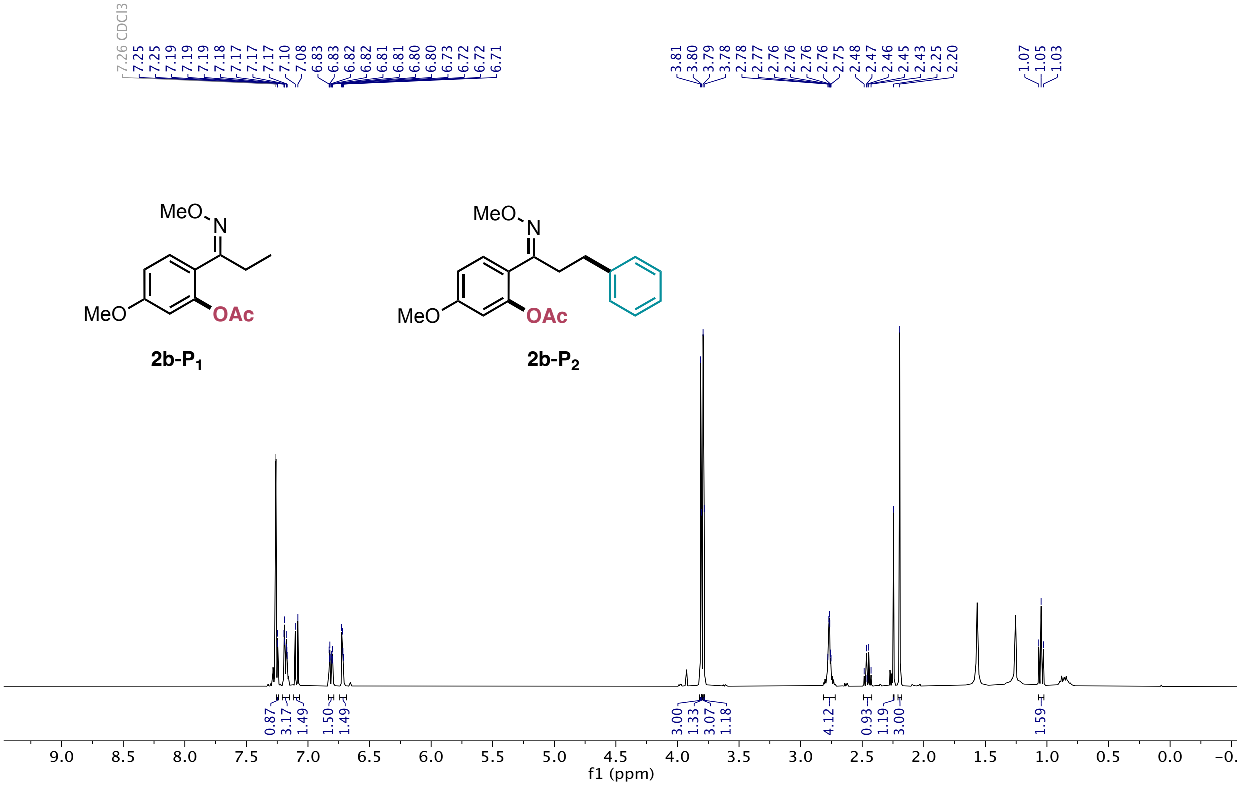

^{13}C{H} NMR (101 MHz, $CDCl_3$) of (*Z*)-5-methoxy-2-(1-(methoxyimino)-3-phenylpropyl)phenyl acetate (2b)

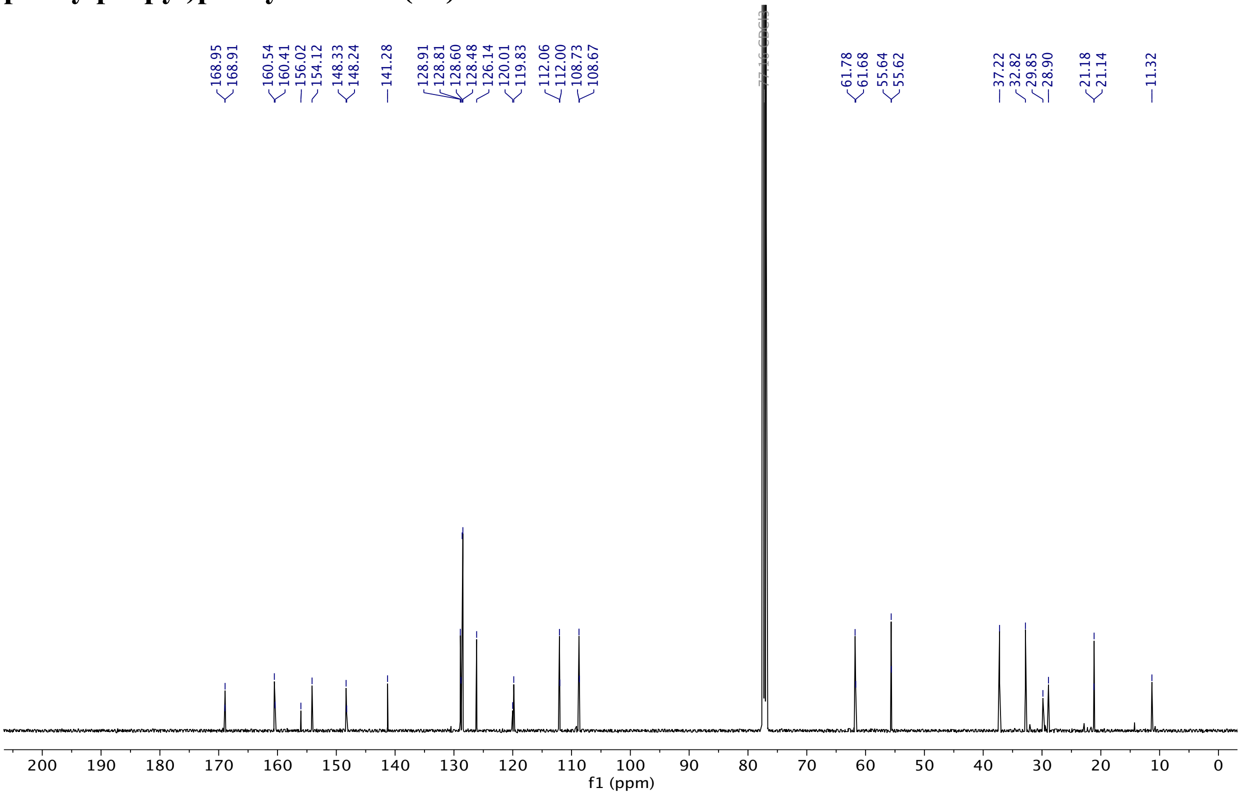

^1H NMR (400 MHz, CD_2Cl_2) of (Z)-5-(tert-butyl)-2-(1-(methoxyimino)-3-phenylpropyl)phenyl acetate (2c)

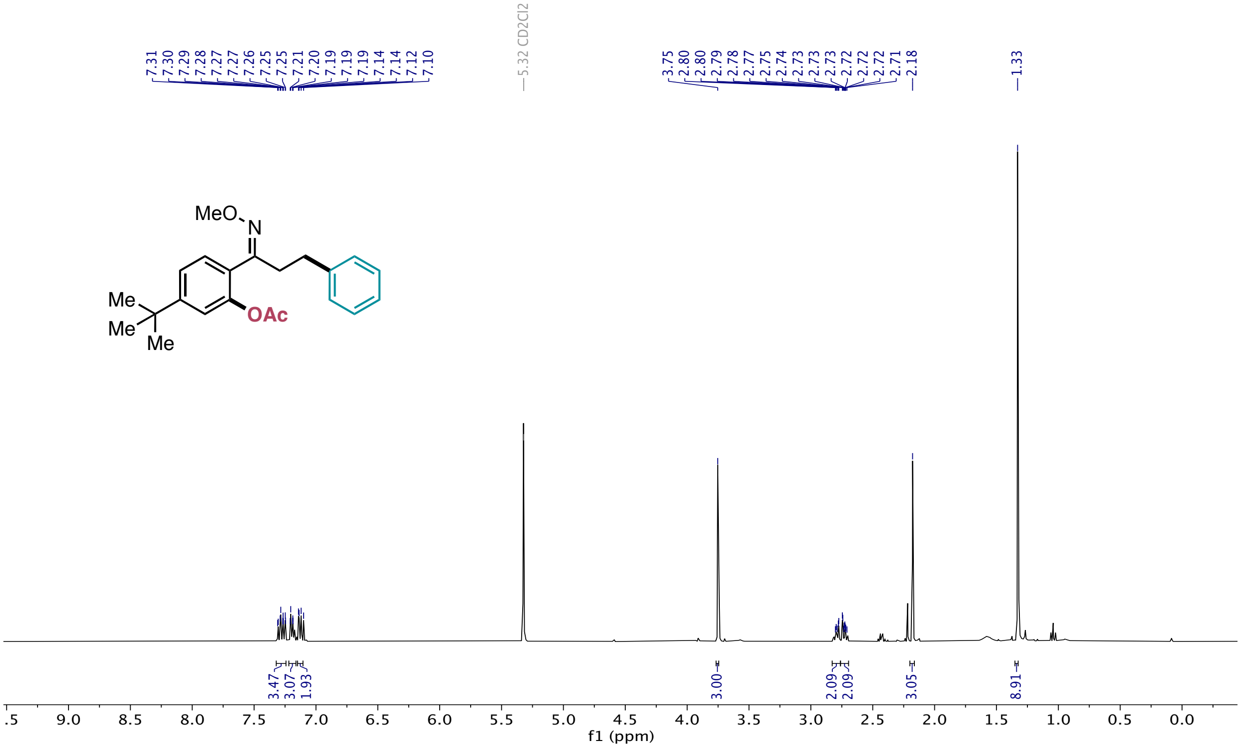

$^{13}C\{H\}$ NMR (101 MHz, $CDCl_3$) of (Z)-5-(tert-butyl)-2-(1-(methoxyimino)-3-phenylpropyl)phenyl acetate (2c)

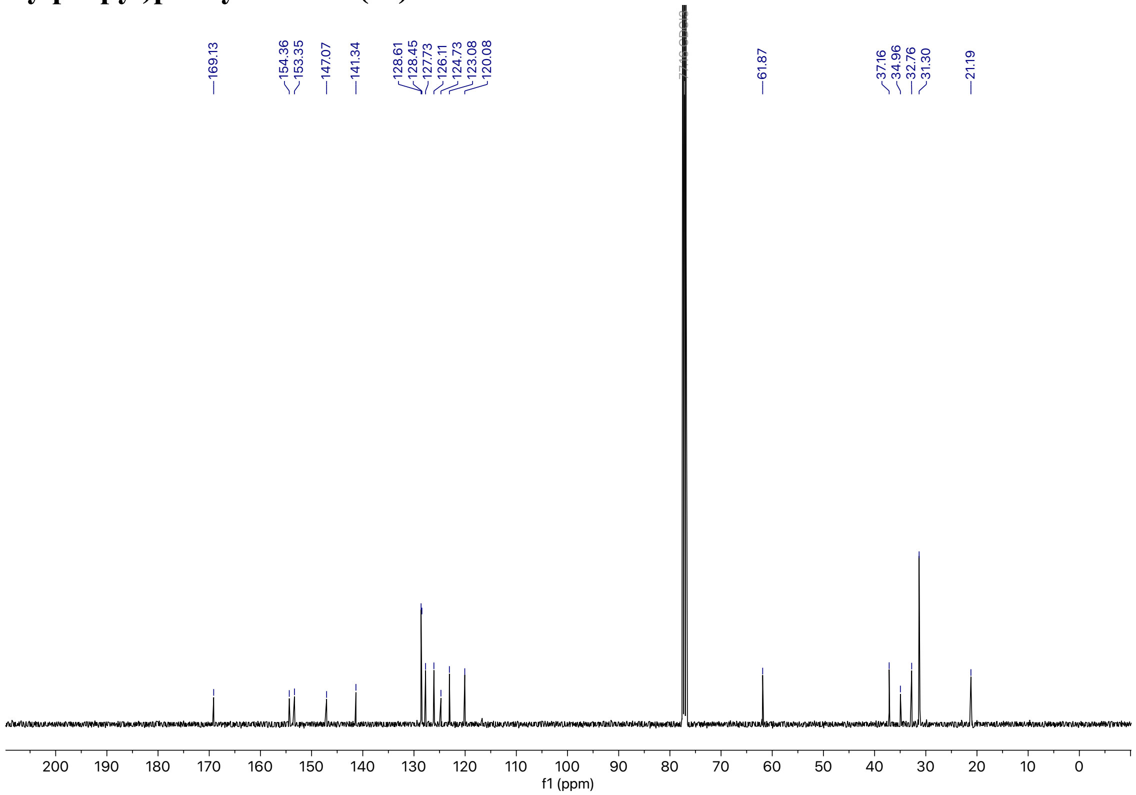

^{1}H NMR (400 MHz, $CDCl_3$) of (Z)-5-fluoro-2-(1-(methoxyimino)-3-phenylpropyl)phenyl acetate (2d)

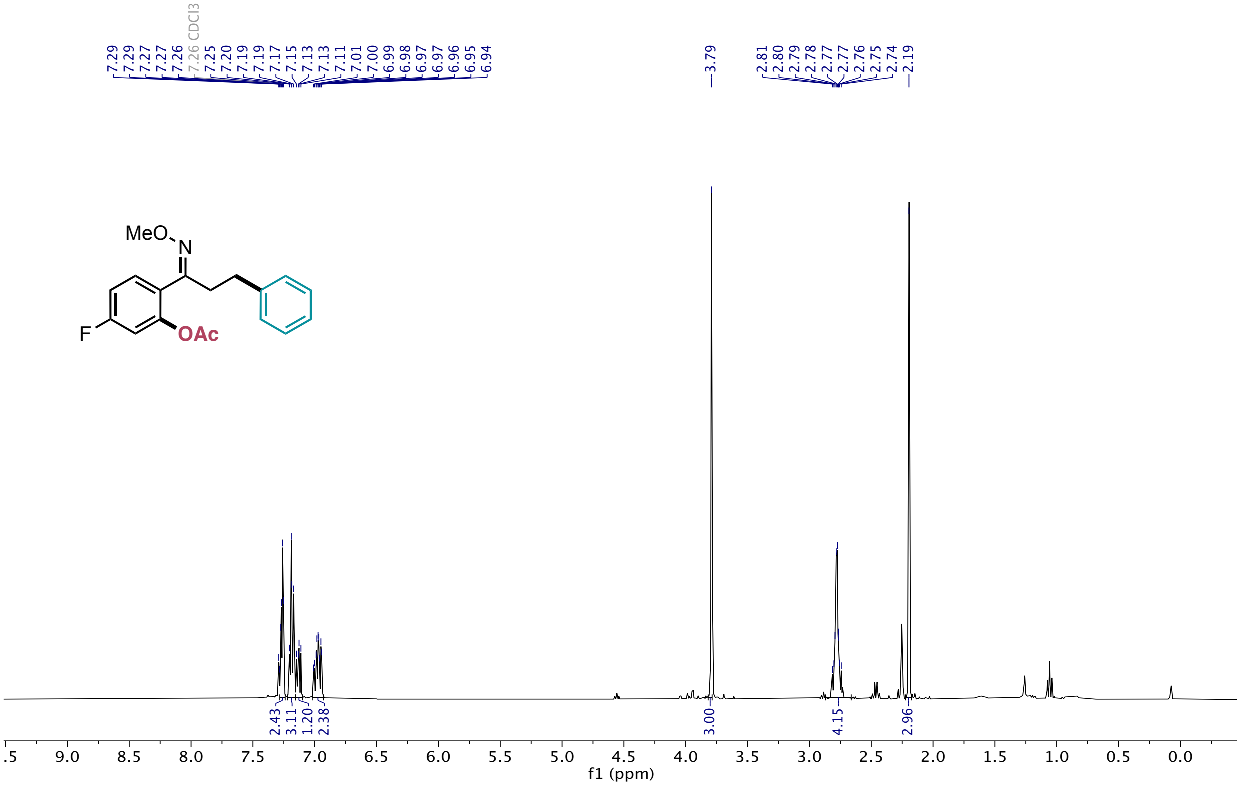

^{13}C{H} NMR (101 MHz, $CDCl_3$) of (Z)-5-fluoro-2-(1-(methoxyimino)-3-phenylpropyl)phenyl acetate (2d)

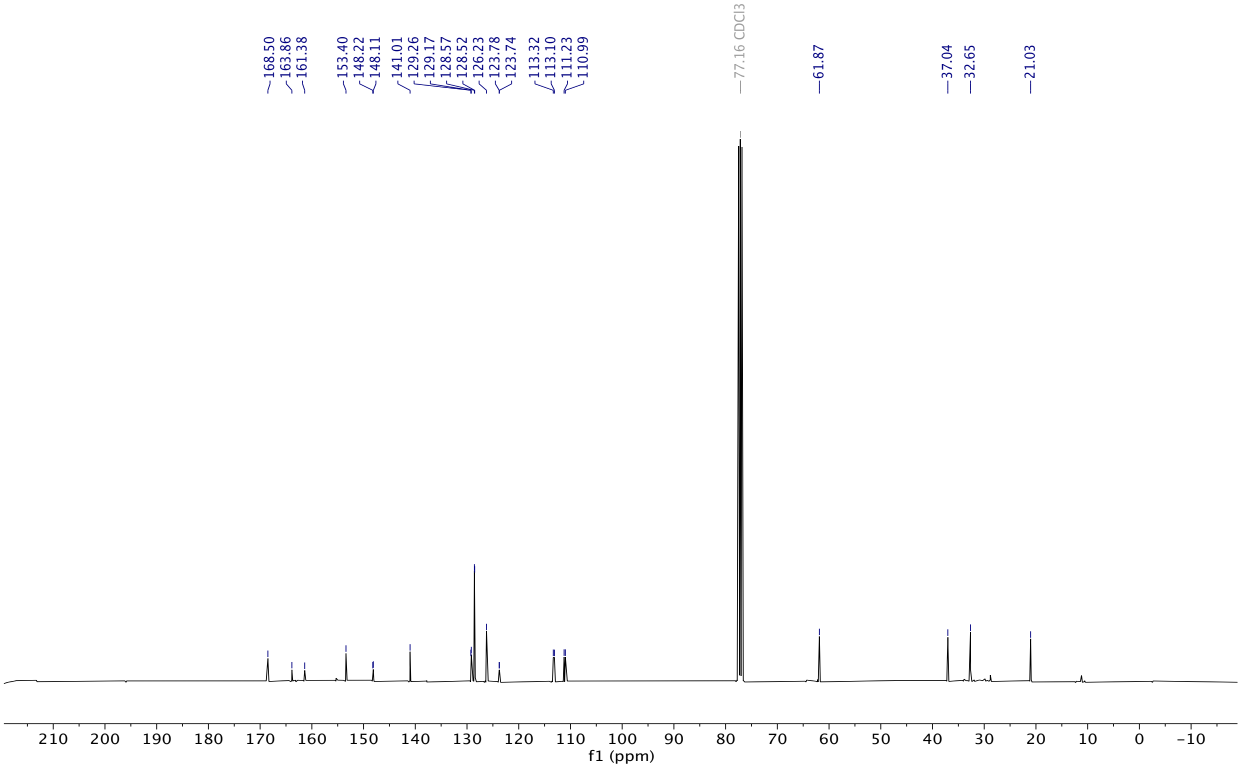

^{19}F NMR (377 MHz, $CDCl_3$) of (Z)-5-fluoro-2-(1-(methoxyimino)-3-phenylpropyl)phenyl acetate (2d)

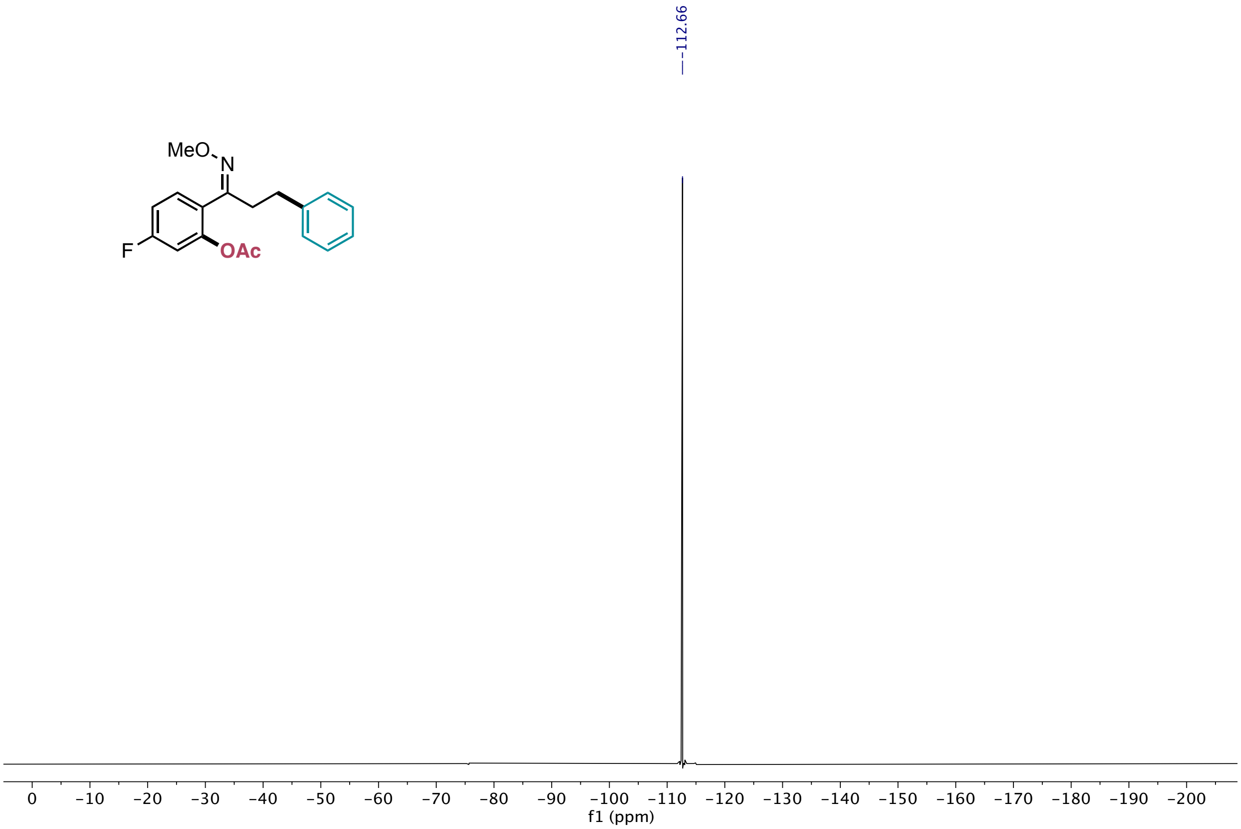

^{1}H NMR (400 MHz, $CDCl_3$) of (*Z*)-2-(1-(methoxyimino)-3-phenylpropyl)-5-(trifluoromethyl) phenyl acetate (2e)

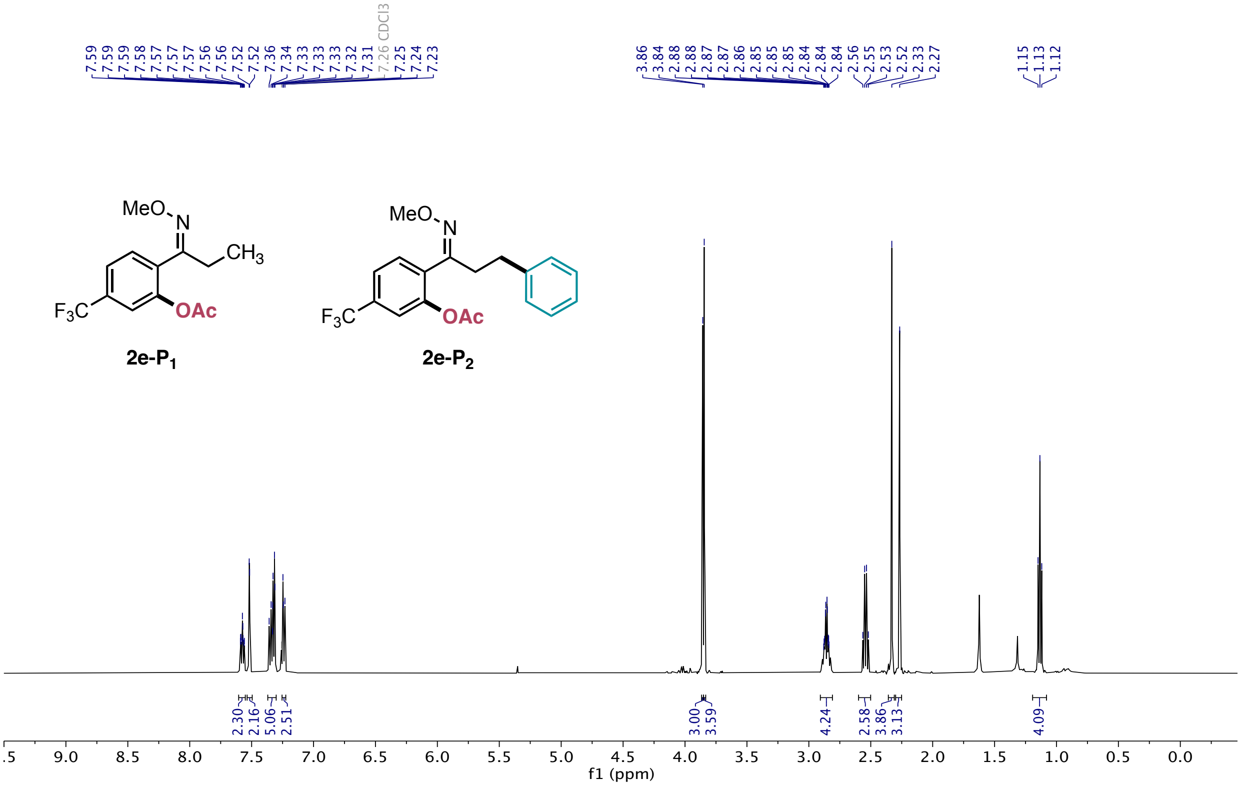

^{13}C NMR (101 MHz, $CDCl_3$) of (*Z*)-2-(1-(methoxyimino)-3-phenylpropyl)-5-(trifluoromethyl) phenyl acetate (2e)

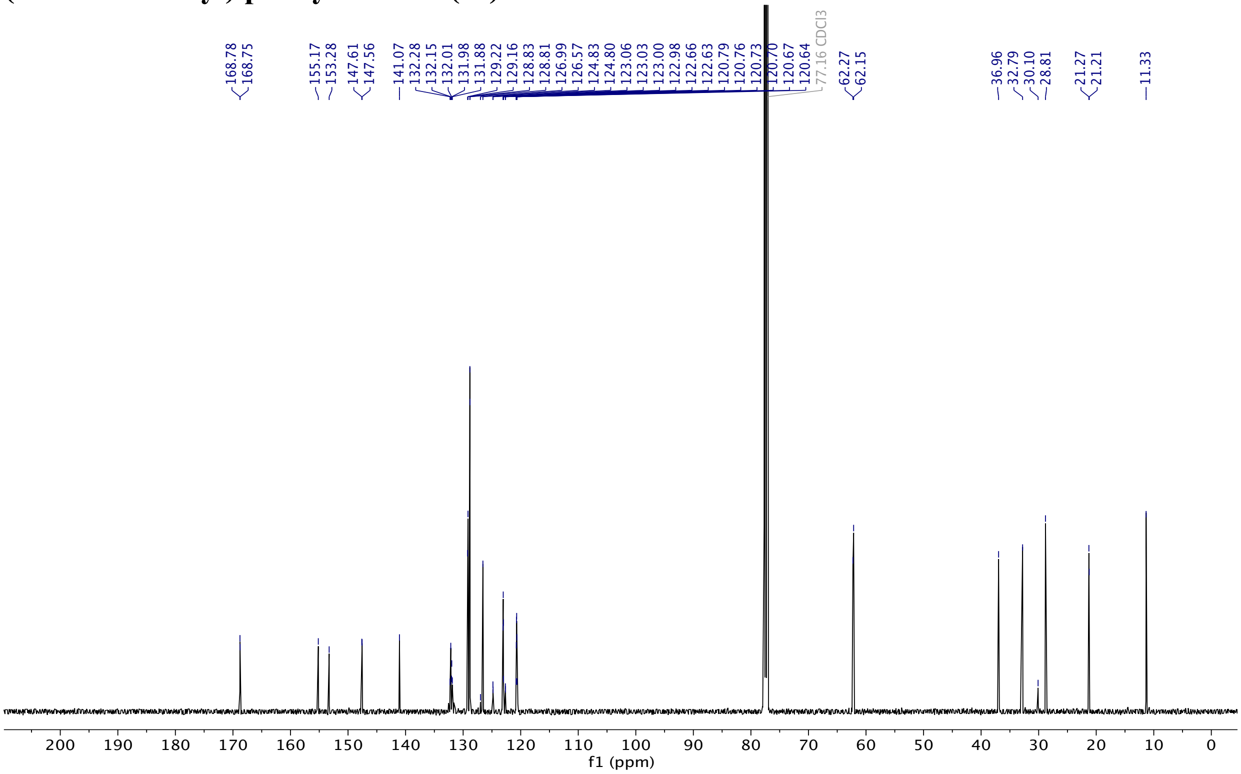

^{19}F NMR (471 MHz, $CDCl_3$) of (*Z*)-2-(1-(methoxyimino)-3-phenylpropyl)-5-(trifluoromethyl) phenyl acetate (2e)

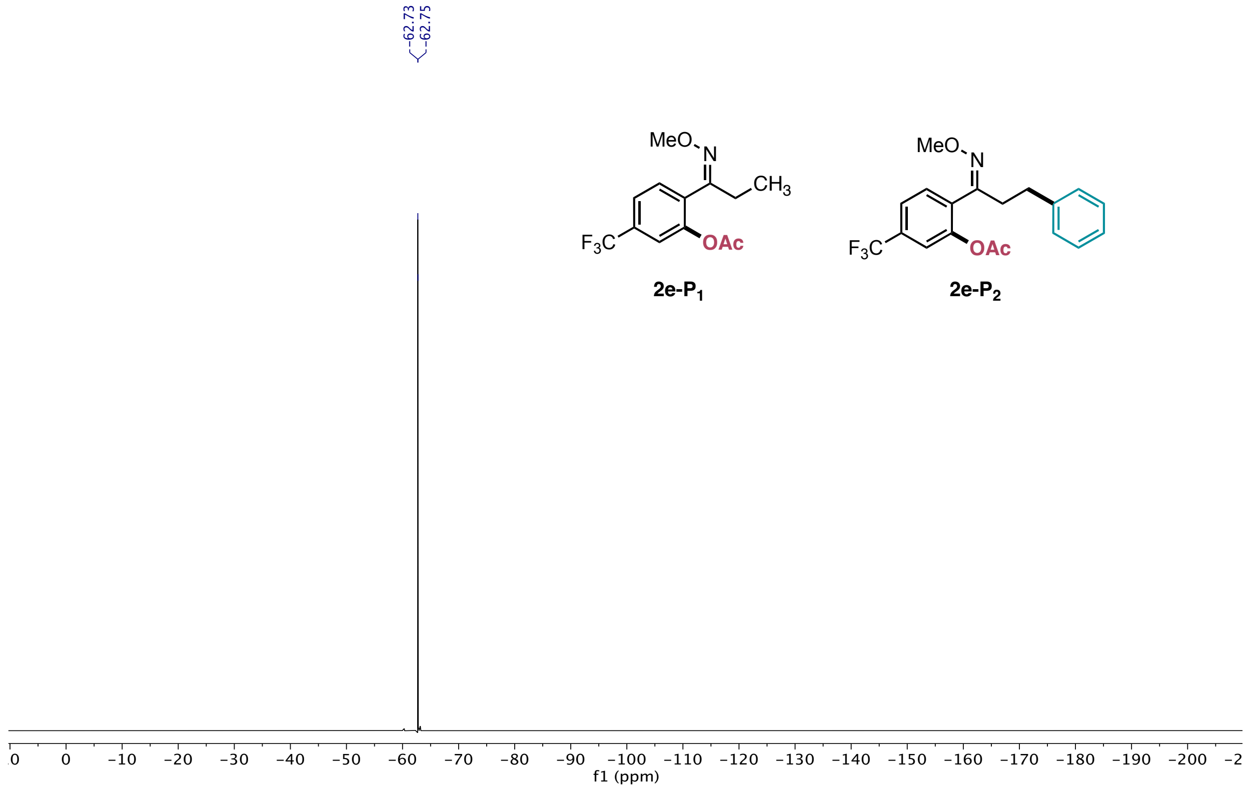

^{1}H NMR (500 MHz, $CDCl_3$) of (Z)-2-(1-(methoxyimino)-3-phenylpropyl)-6-methylphenyl acetate (2f)

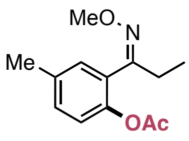

2f-P$_1$

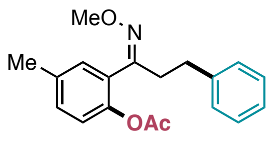

2f-P$_2$

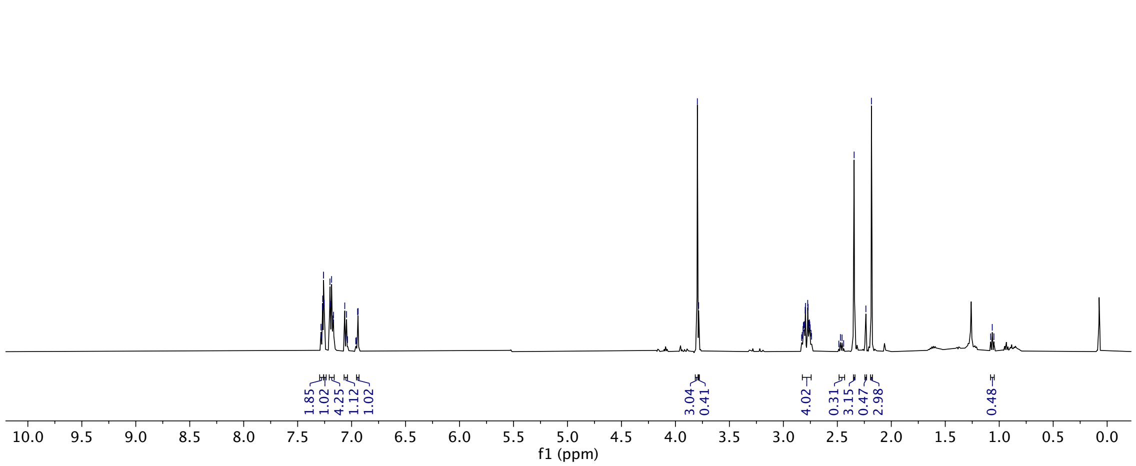

^{13}C NMR (126 MHz, $CDCl_3$) of (Z)-2-(1-(methoxyimino)-3-phenylpropyl)-6-methylphenyl acetate (2f)

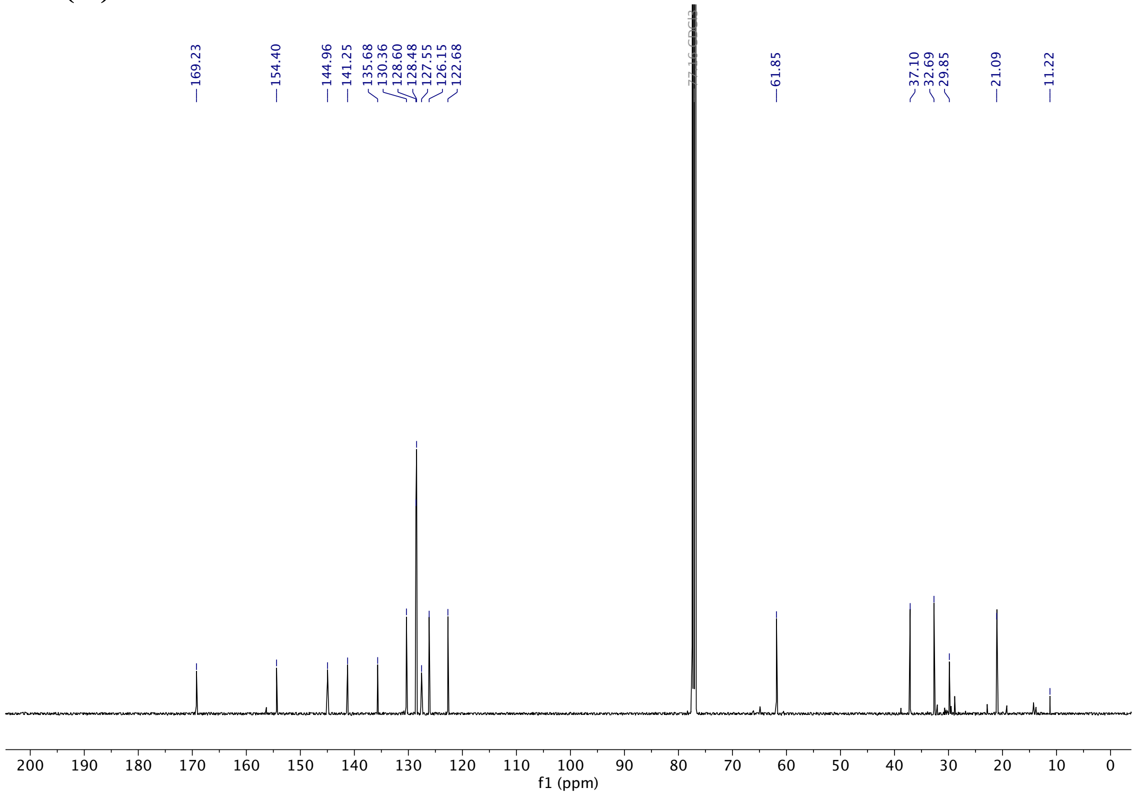

^{1}H NMR (500 MHz, CDCl$_3$) of (*Z*)-4-chloro-2-(1-(methoxyimino)-3-phenylpropyl) phenyl acetate (2g)

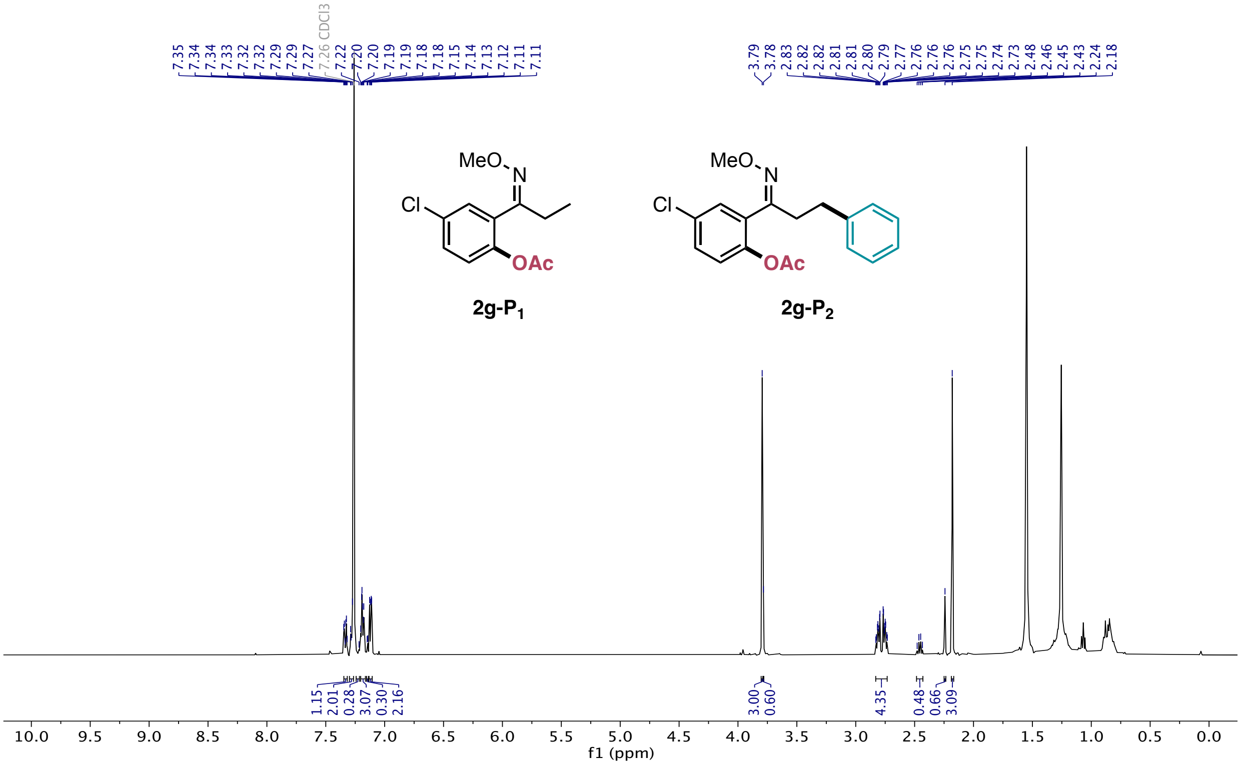

^{13}C NMR (126 MHz, CDCl$_3$) of (*Z*)-4-chloro-2-(1-(methoxyimino)-3-phenylpropyl) phenyl acetate (2g)

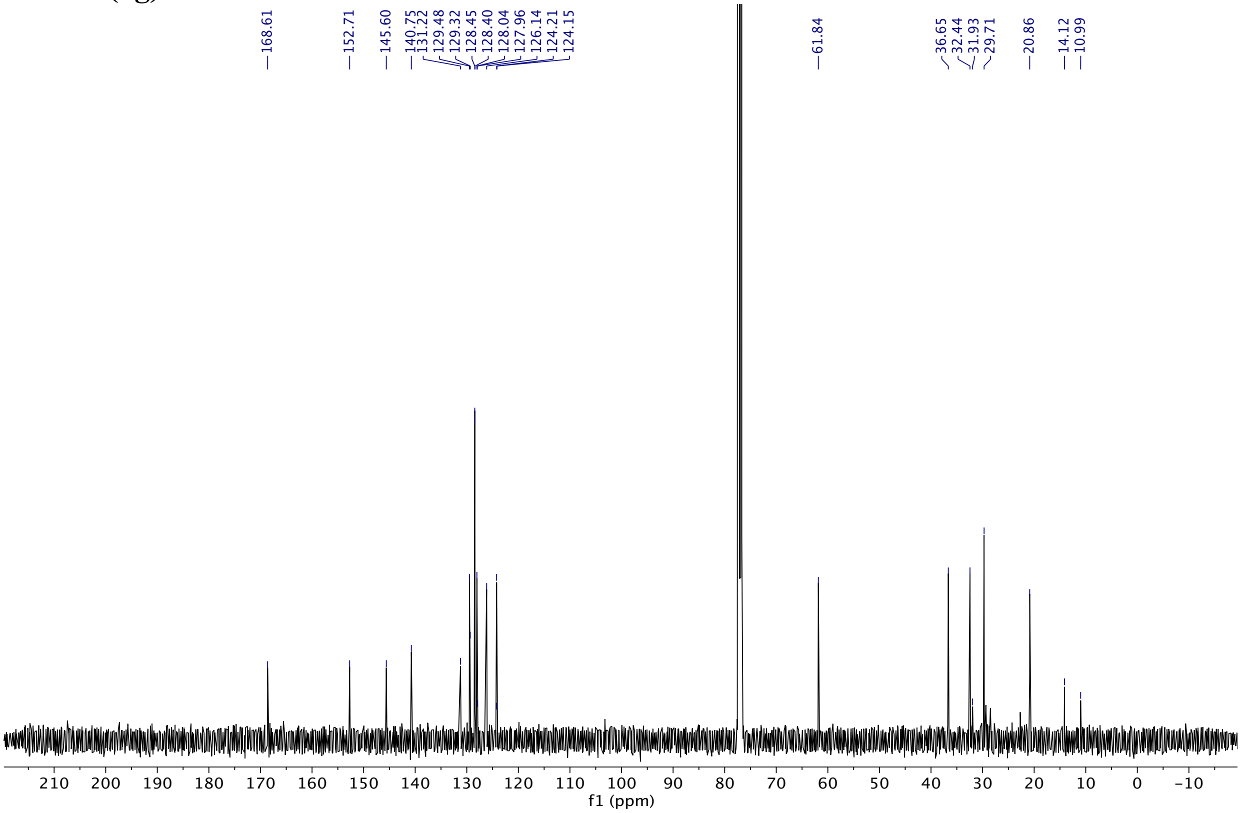

^{1}H NMR (400 MHz, $CDCl_3$) of (Z)-1-(2-methoxyphenyl)-3-phenylpropan-1-one O-methyl oxime (2h)

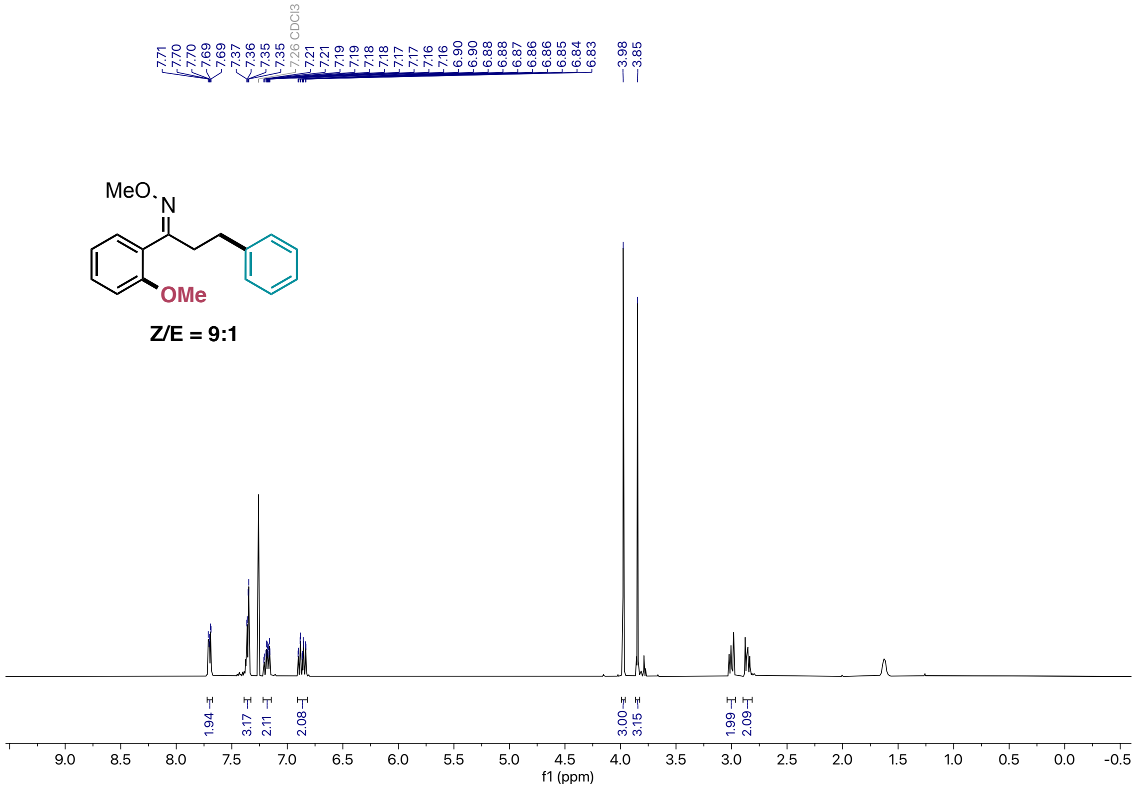

$^{13}C\{H\}$ NMR (101 MHz, $CDCl_3$) of (Z)-1-(2-methoxyphenyl)-3-phenylpropan-1-one O-methyl oxime (2h)

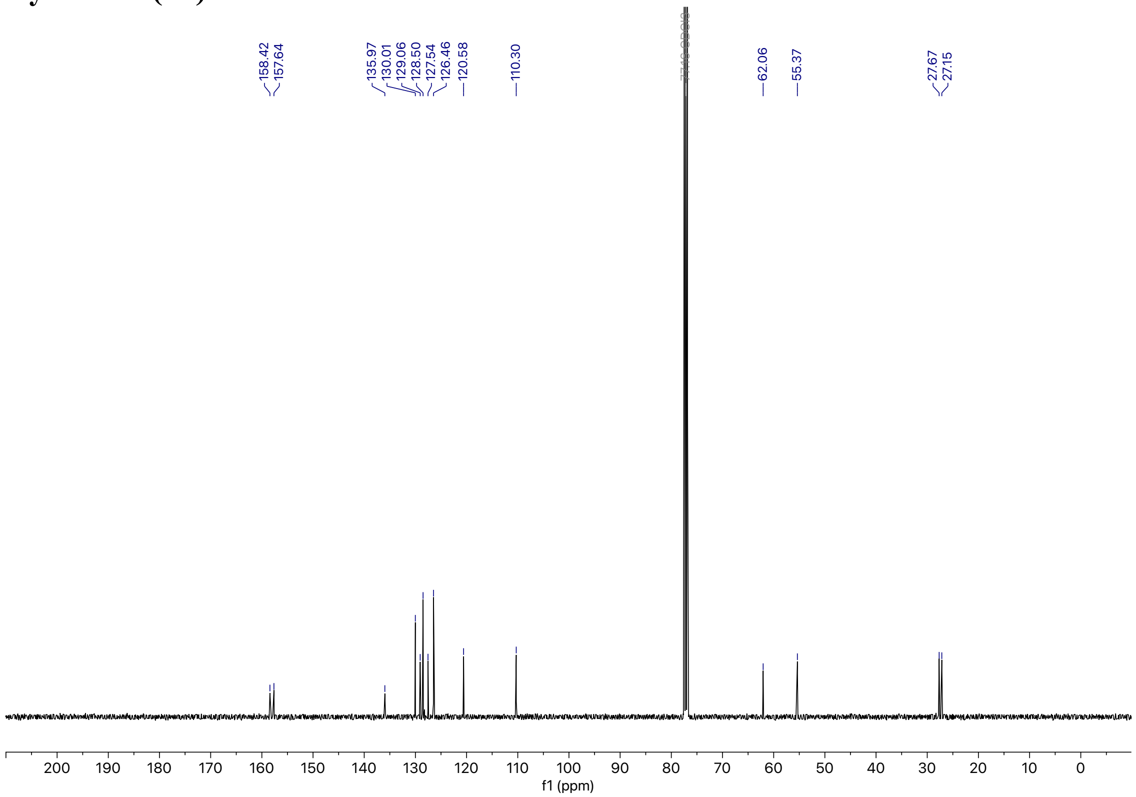

^{1}H NMR (400 MHz, CD_2Cl_2) of (*Z*)-3-(1-(methoxyimino)-3-phenylpropyl)-5,6,7,8-tetrahydronaphthalen-2-yl acetate (2i)

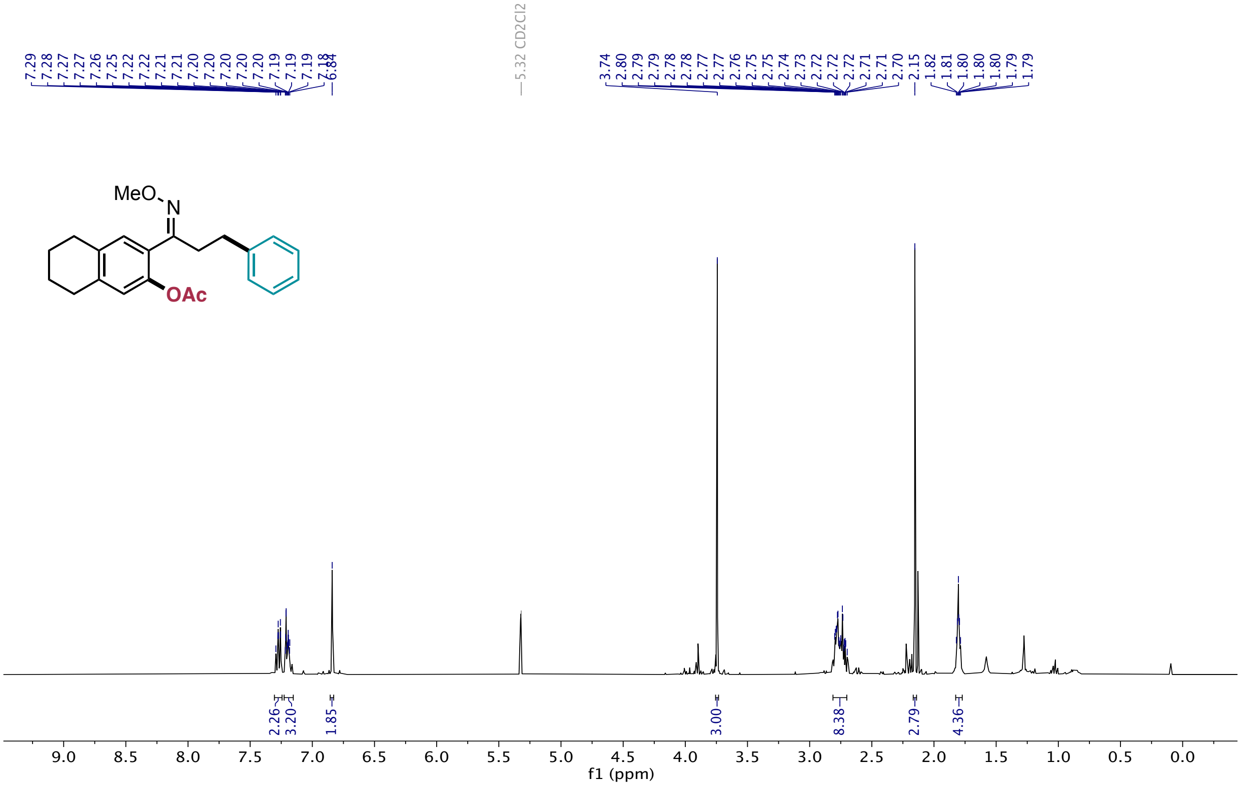

^{13}C{H} NMR (101 MHz, CD_2Cl_2) of (*Z*)-3-(1-(methoxyimino)-3-phenylpropyl)-5,6,7,8-tetrahydronaphthalen-2-yl acetate (2i)

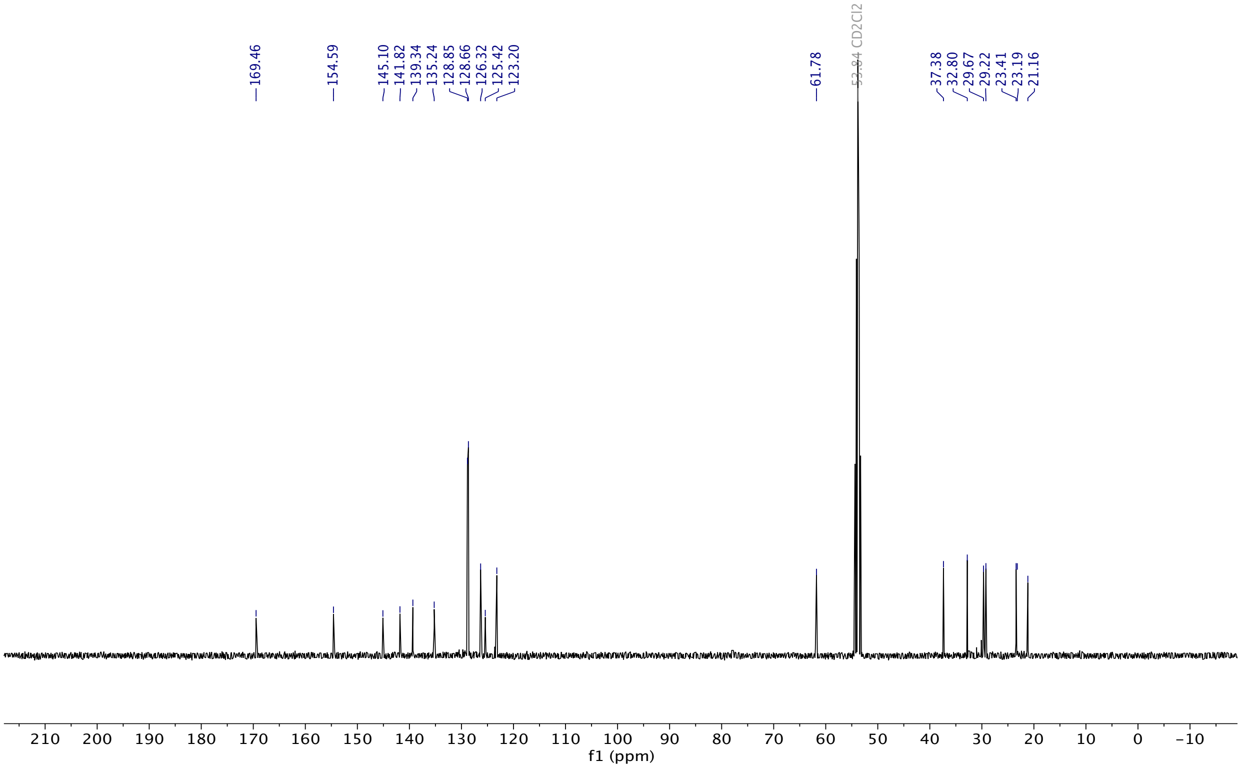

^{1}H NMR (400 MHz, $CDCl_3$) of (Z)-5-(1-(methoxyimino)-3-phenylpropyl)benzo[d][1,3]dioxol-4-yl acetate (2j)

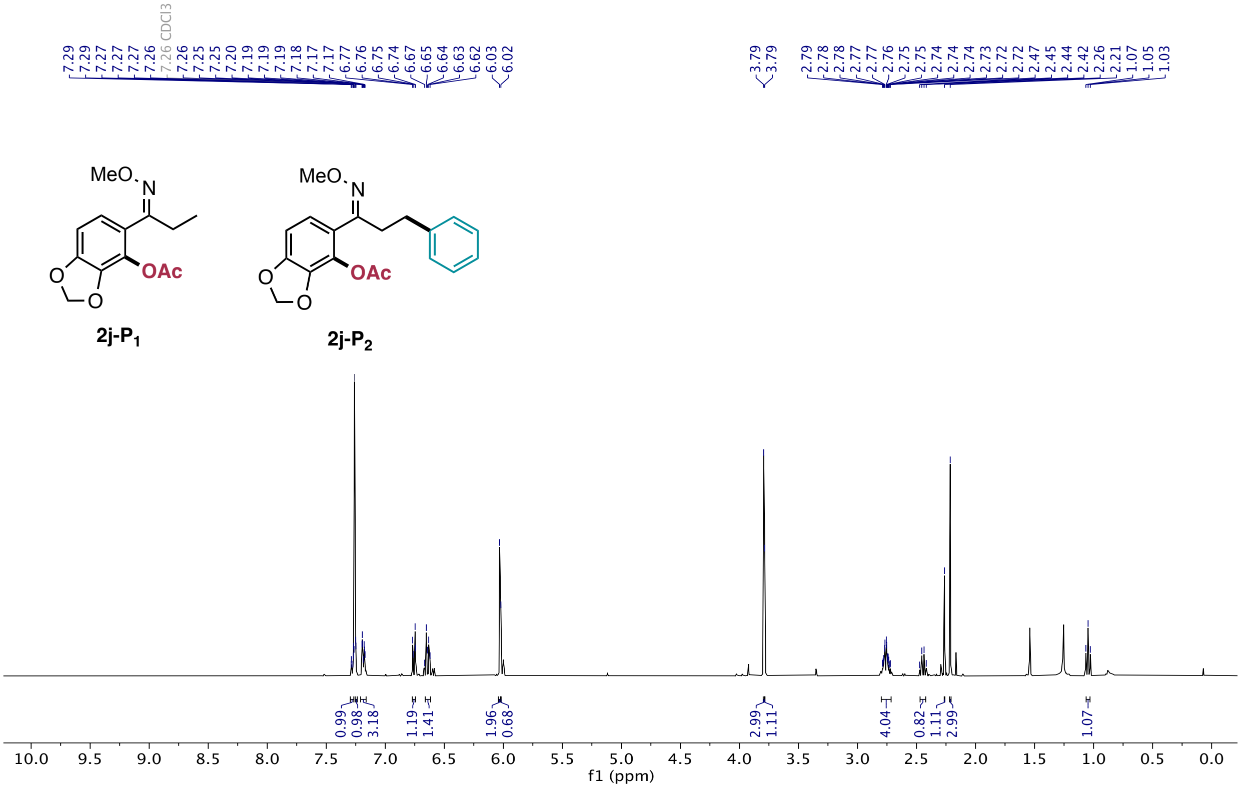

^{13}C NMR (101 MHz, $CDCl_3$) of (Z)-5-(1-(methoxyimino)-3-phenylpropyl)benzo[d][1,3]dioxol-4-yl acetate (2j)

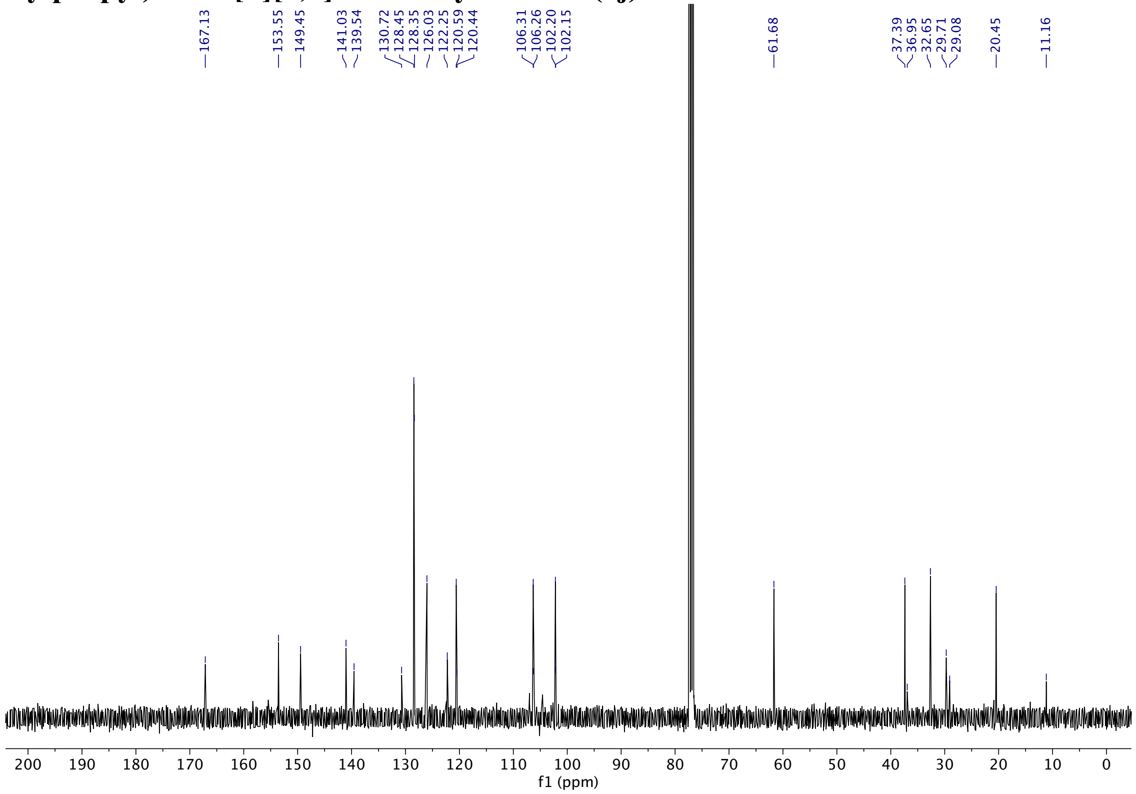

^{1}H NMR (500 MHz, $CDCl_3$) of (*Z*)-7-benzyl-8-(methoxyimino)-5,6,7,8-tetrahydronaphthalen-1-yl acetate (2n)

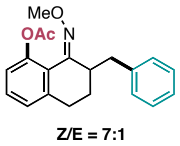

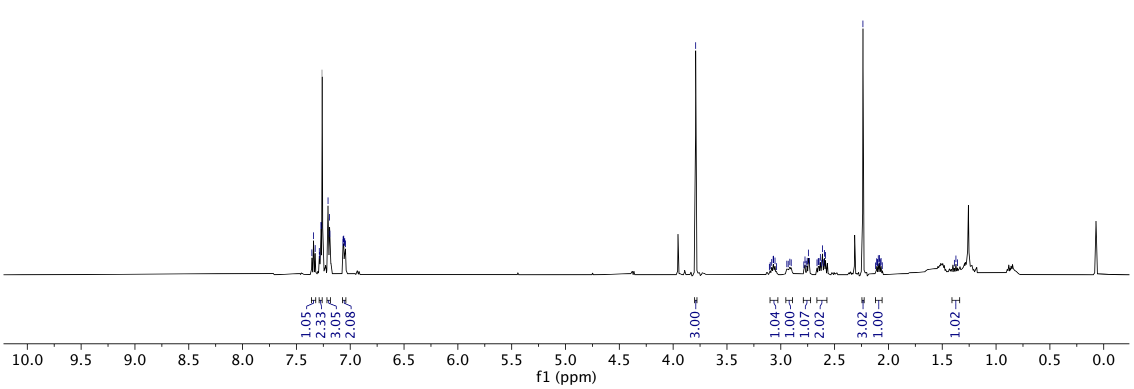

^{13}C NMR (126 MHz, $CDCl_3$) of (*Z*)-7-benzyl-8-(methoxyimino)-5,6,7,8-tetrahydronaphthalen-1-yl acetate (2n)

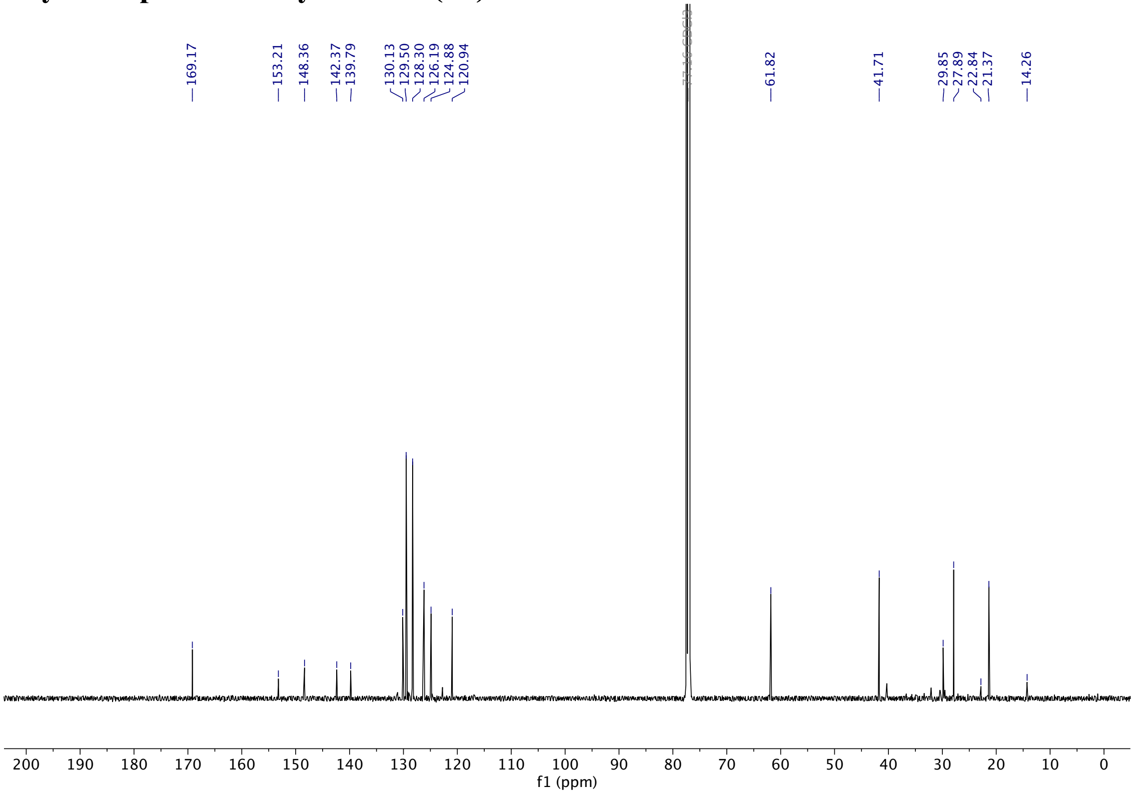

^{1}H NMR (500 MHz, CDCl$_3$) of (Z)-2-(2-(methoxyimino)-4-phenylbutanoyl)phenyl acetate (2p)

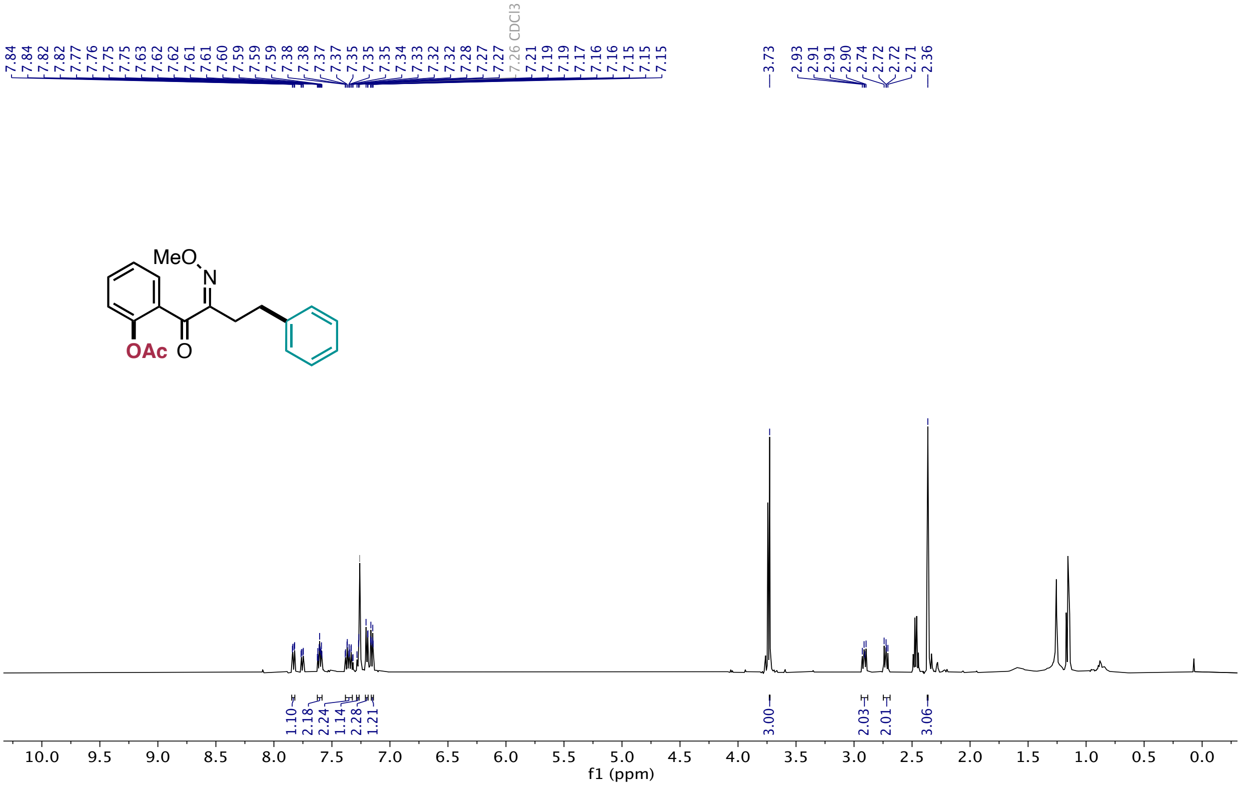

^{13}C NMR (126 MHz, CDCl$_3$) of (Z)-2-(2-(methoxyimino)-4-phenylbutanoyl)phenyl acetate (2p)

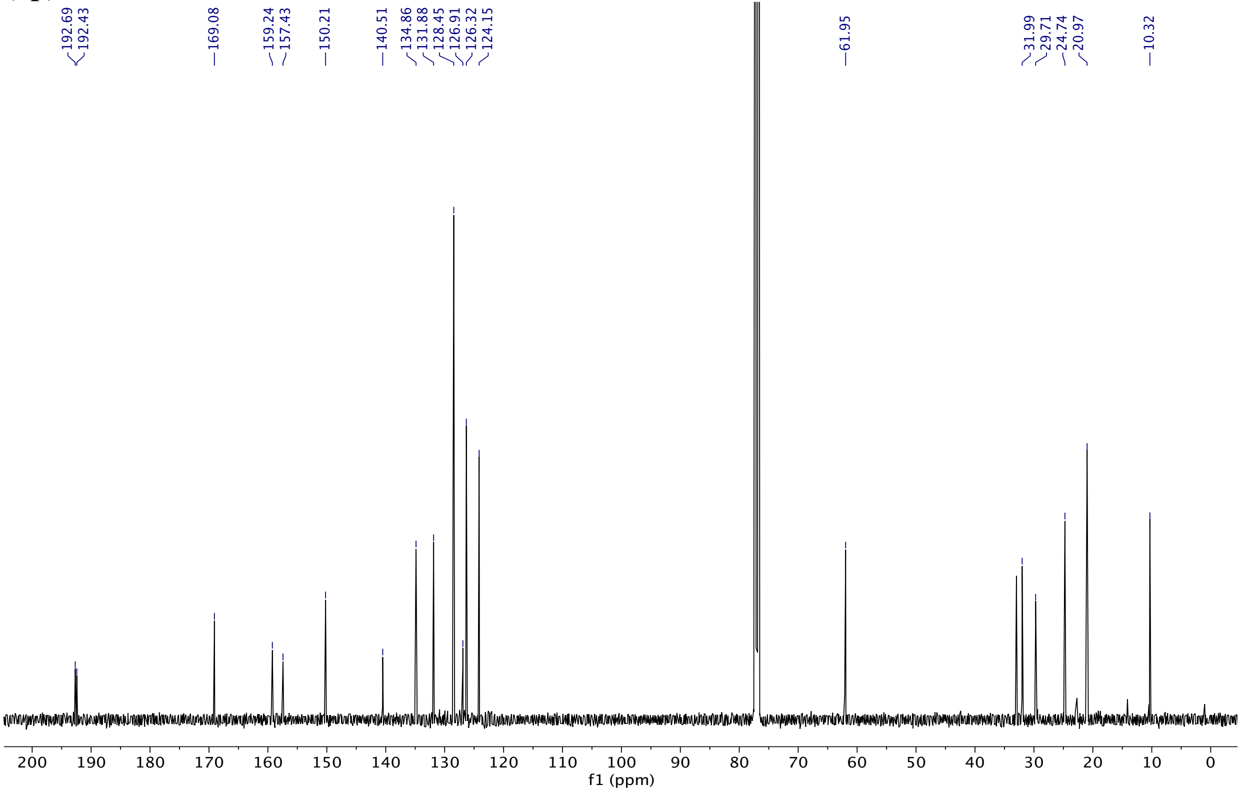

^{1}H NMR (500 MHz, $CDCl_3$) of Methyl (*R*,*E*)-2-(2-(2-acetoxyphenyl)-2-(methoxyimino)acetamido)-4-phenylbutanoate (2q)

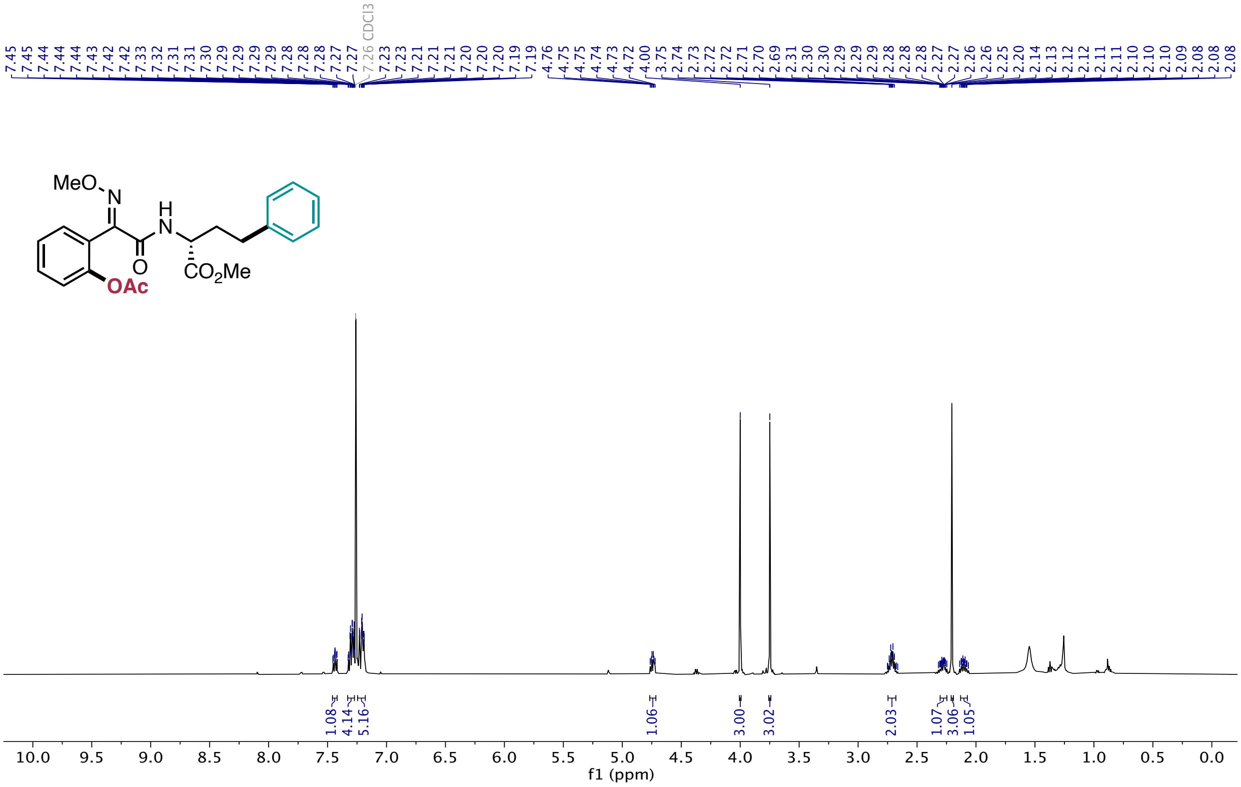

^{13}C NMR (126 MHz, $CDCl_3$) of Methyl (*R*,*E*)-2-(2-(2-acetoxyphenyl)-2-(methoxyimino)acetamido)-4-phenylbutanoate (2q)

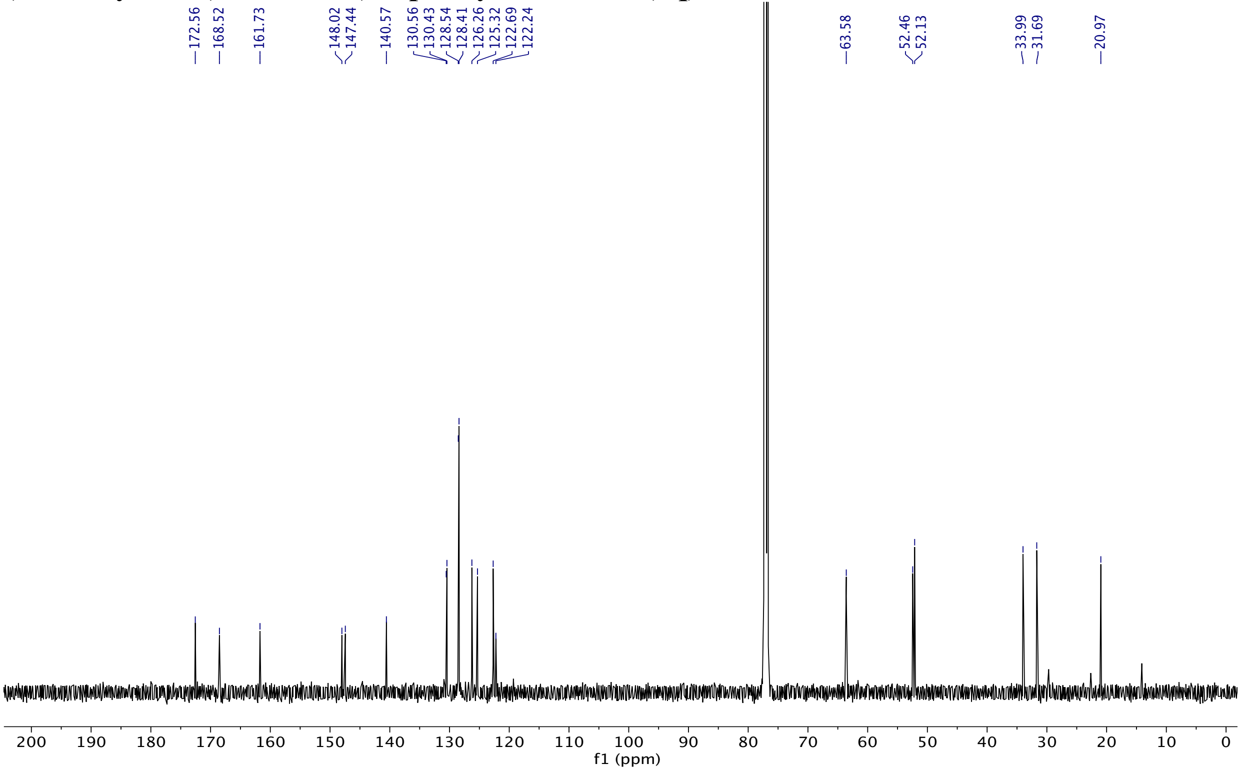

^{1}H NMR (400 MHz, CD_2Cl_2) of (Z)-2-(1-(methoxyimino)-3-phenylpropyl)phenyl pivalate (2s)

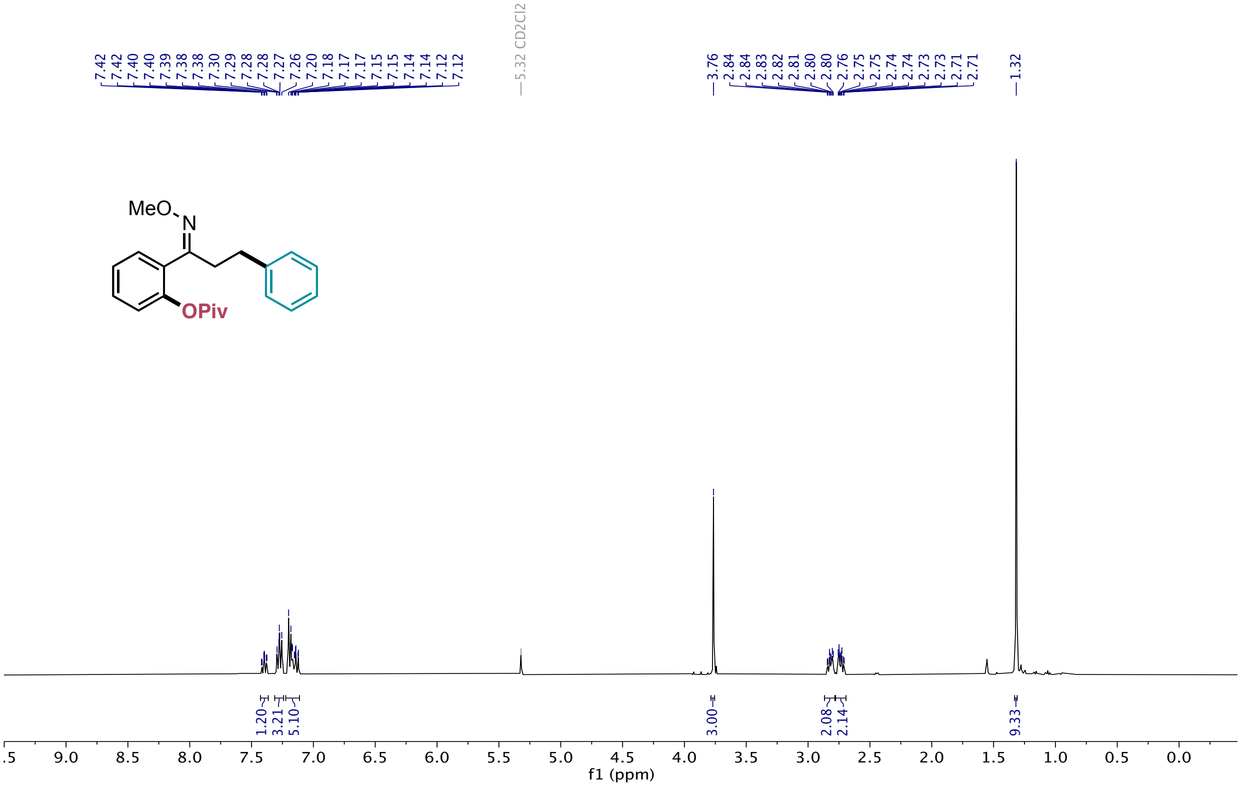

^{13}C{H} NMR (101 MHz, CD_2Cl_2) of (Z)-2-(1-(methoxyimino)-3-phenylpropyl)phenyl pivalate (2s)

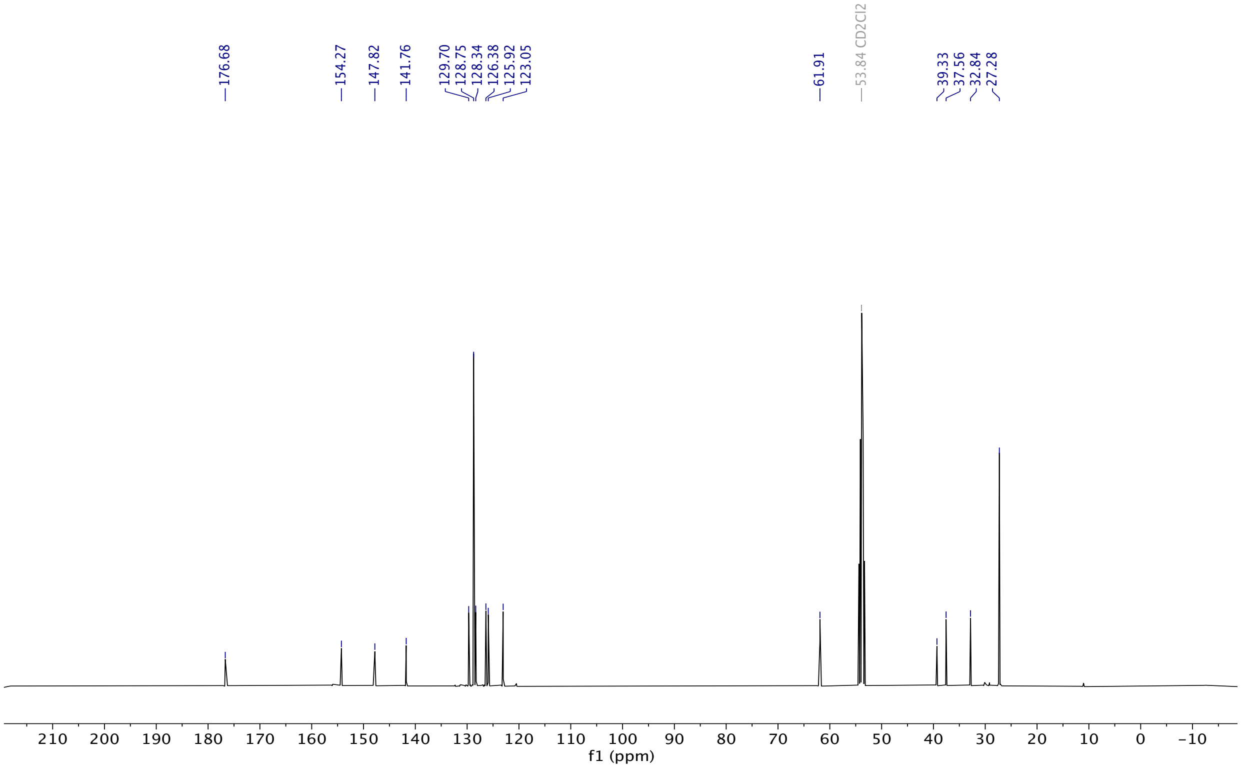

^{1}H NMR (400 MHz, CDCl$_3$) of (Z)-2-(1-(methoxyimino)-3-phenylpropyl)phenyl benzoate (2t)

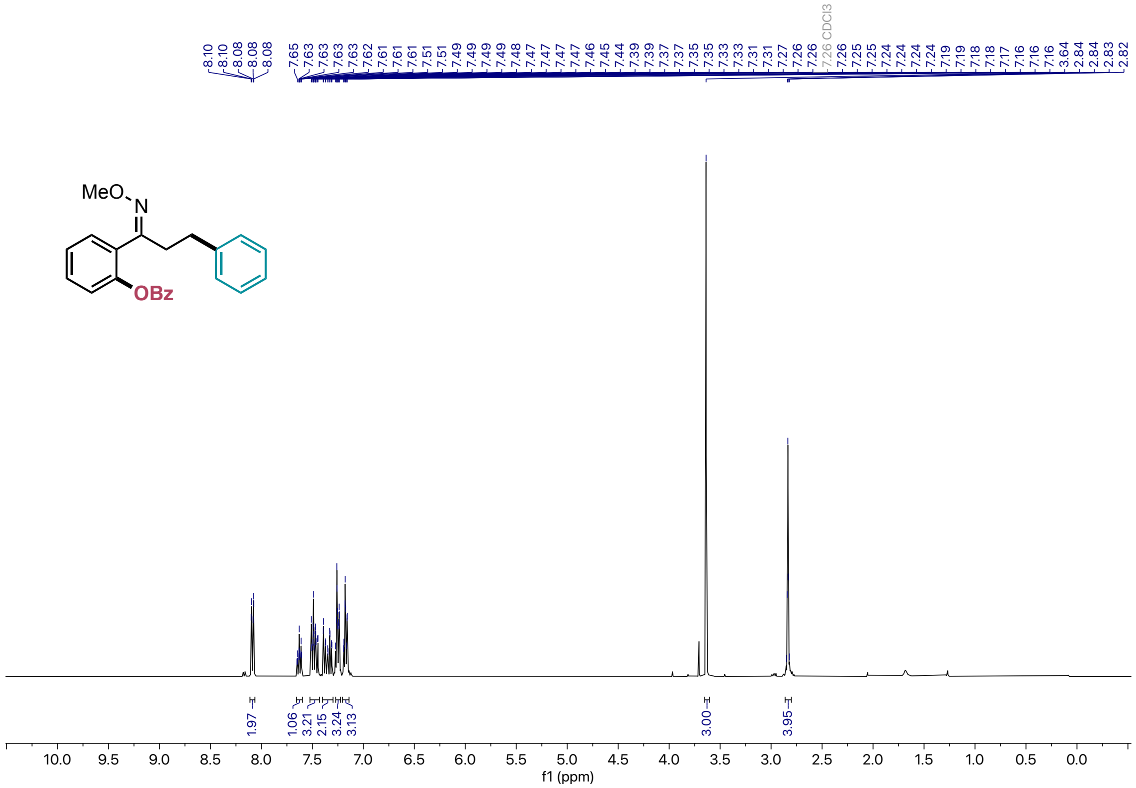

^{13}C{H} NMR (101 MHz, CDCl$_3$) of (Z)-2-(1-(methoxyimino)-3-phenylpropyl)phenyl benzoate (2t)

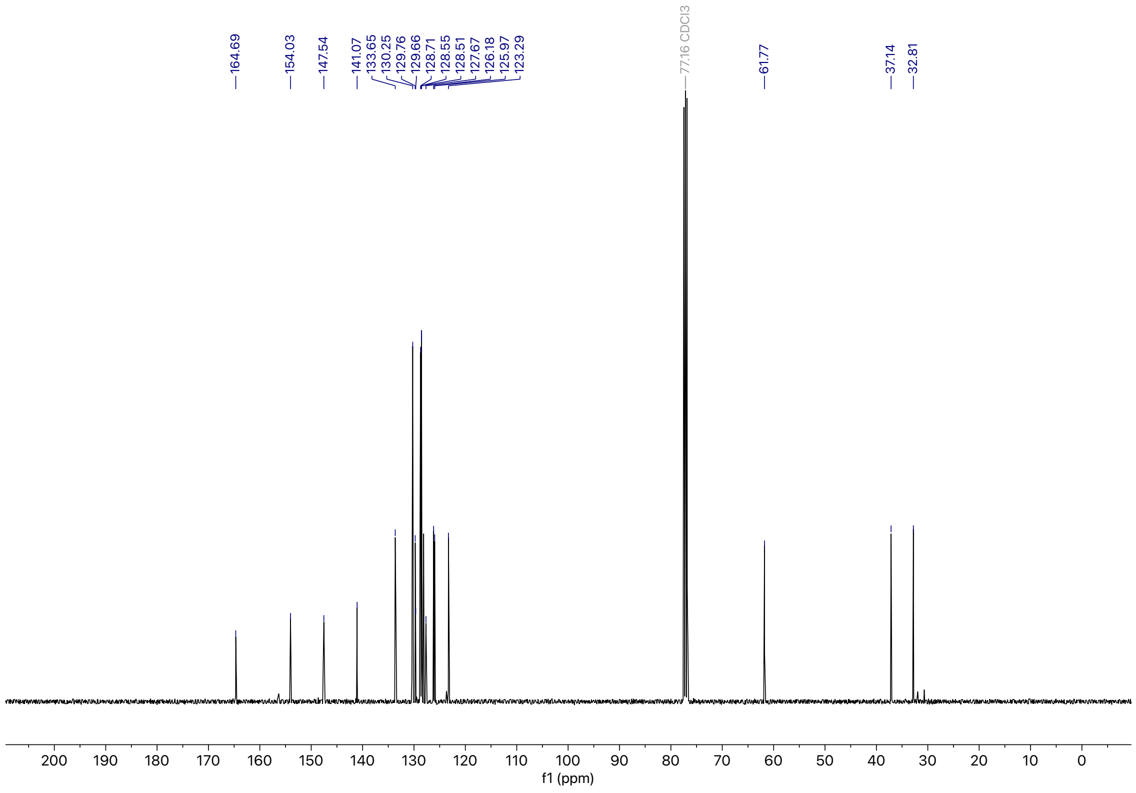

^{1}H NMR (500 MHz, $CDCl_3$) of (*Z*)-2-benzyl-8-(2,2,2-trifluoroethoxy)-3,4-dihydronaphthalen-1(2*H*)-one *O*-methyl oxime (2u)

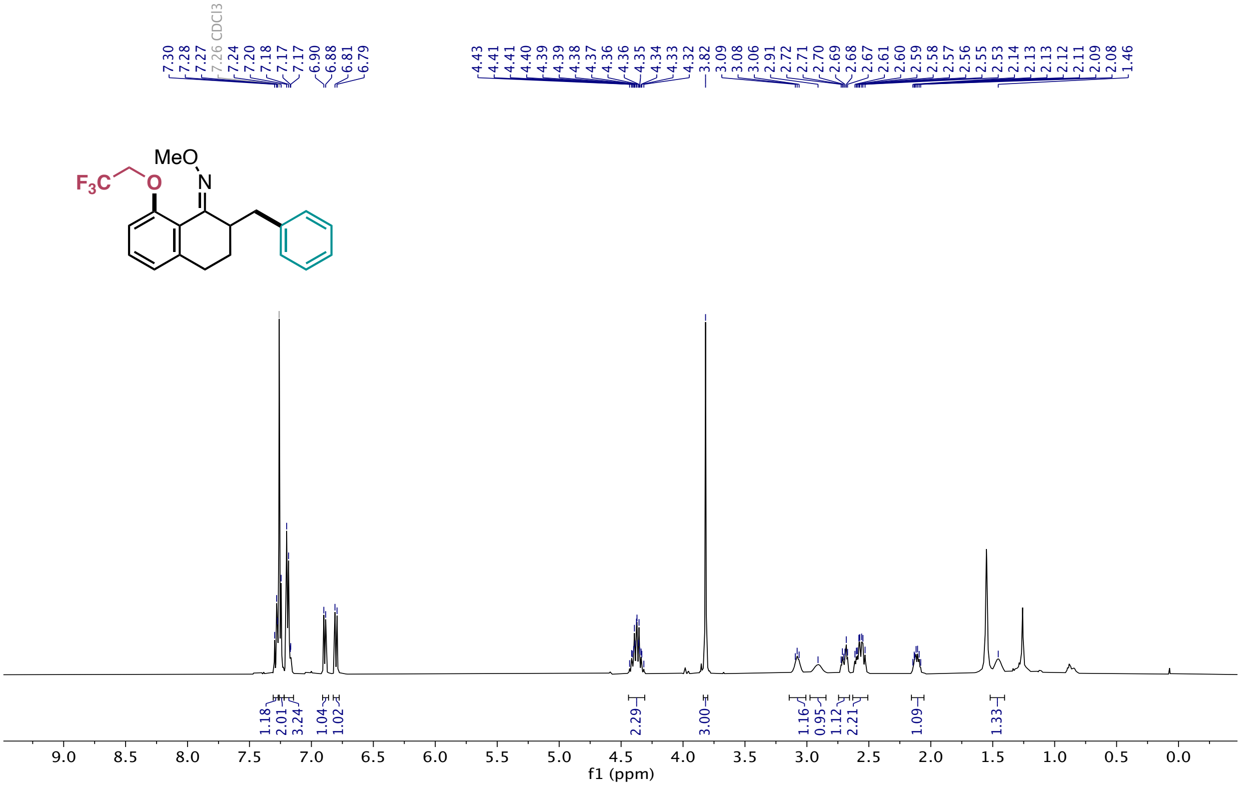

^{13}C{H} NMR (126 MHz, $CDCl_3$) of (*Z*)-2-benzyl-8-(2,2,2-trifluoroethoxy)-3,4-dihydronaphthalen-1(2*H*)-one *O*-methyl oxime (2u)

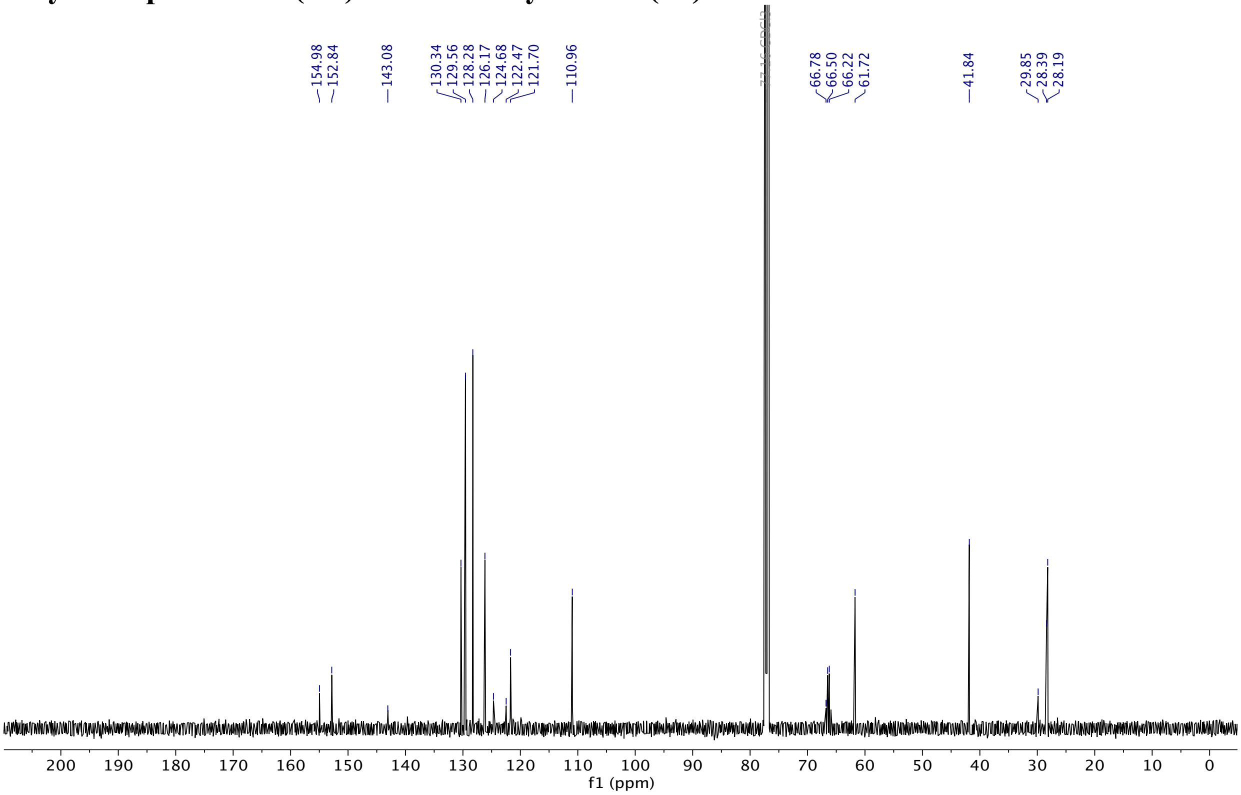

^{19}F NMR (471 MHz, $CDCl_3$) of (*Z*)-2-benzyl-8-(2,2,2-trifluoroethoxy)-3,4-dihydronaphthalen-1(2*H*)-one *O*-methyl oxime (2u)

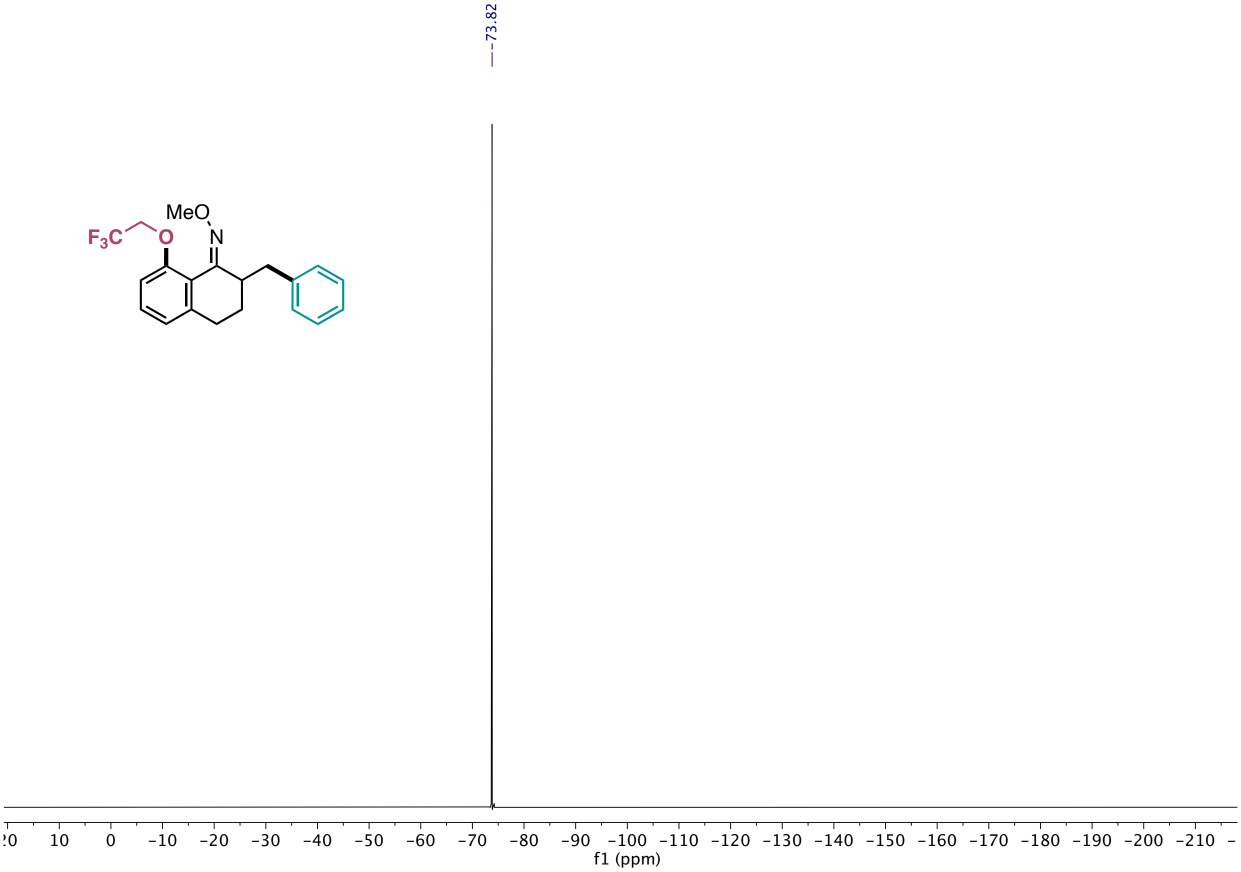

^{1}H NMR (400 MHz, $CDCl_3$) of (Z)-2-(4-(4-fluorophenyl)-2-(methoxyimino)butanoyl) phenyl acetate (2v)

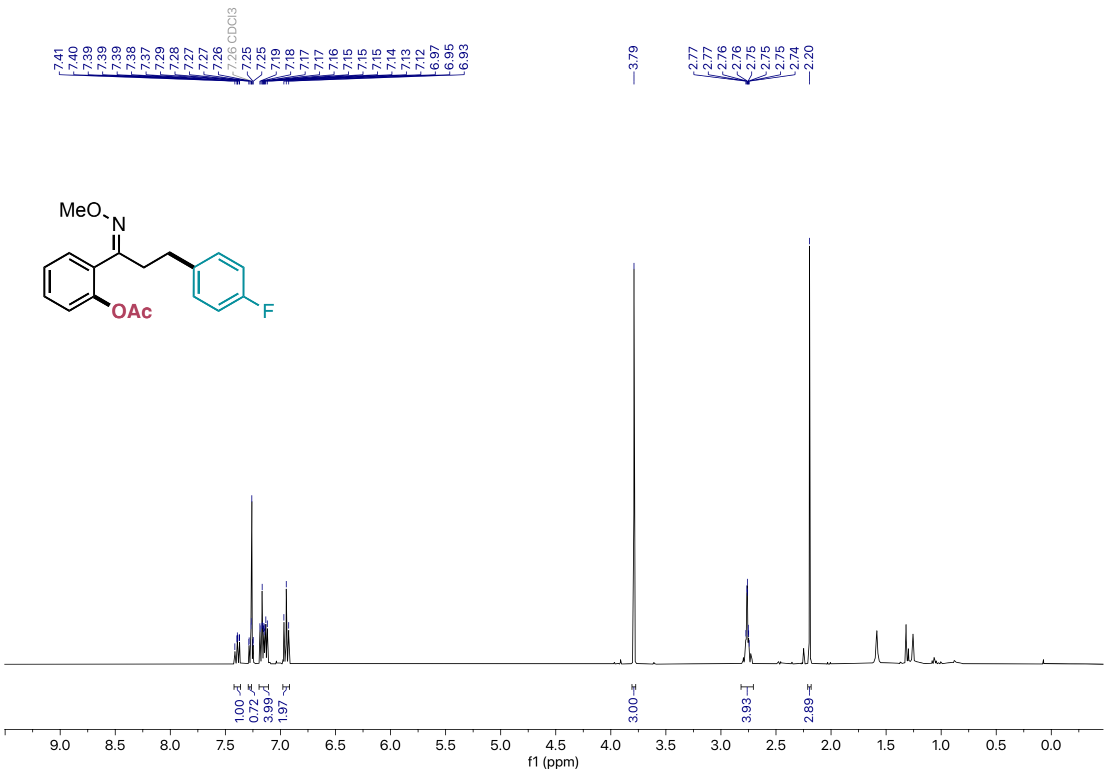

$^{13}C\{H\}$ NMR (101 MHz, $CDCl_3$) of (Z)-2-(4-(4-fluorophenyl)-2-(methoxyimino)butanoyl) phenyl acetate (2v)

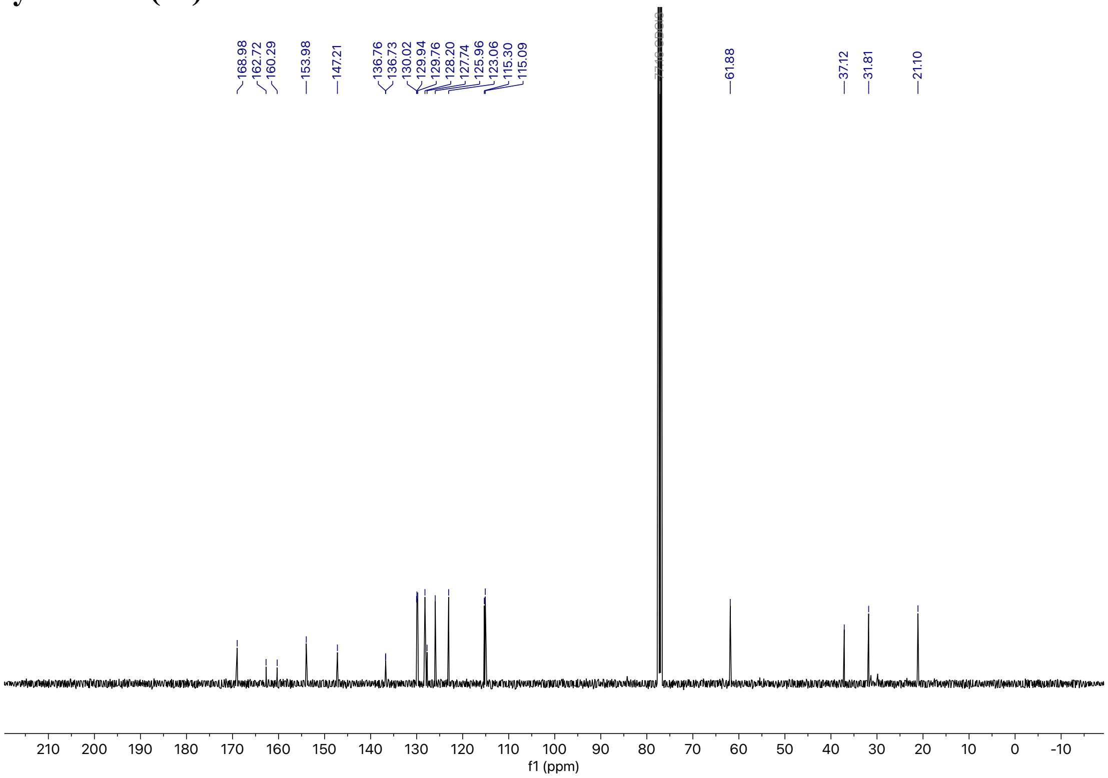

^{19}F NMR (377 MHz, $CDCl_3$) of (Z)-2-(4-(4-fluorophenyl)-2-(methoxyimino)butanoyl) phenyl acetate (2v)

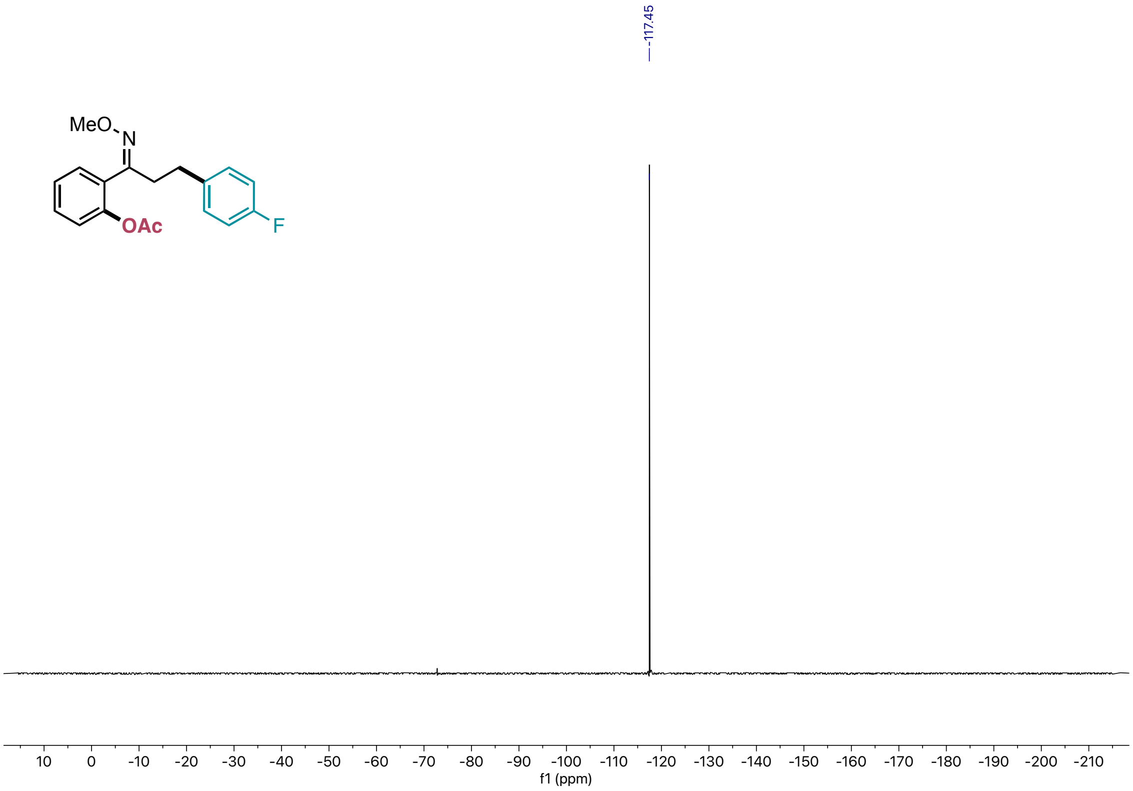

^{1}H NMR (500 MHz, $CDCl_3$) of (*Z*)-2-(3-(4-(*tert*-butyl)phenyl)-1-(methoxyimino)propyl)phenyl acetate (2w)

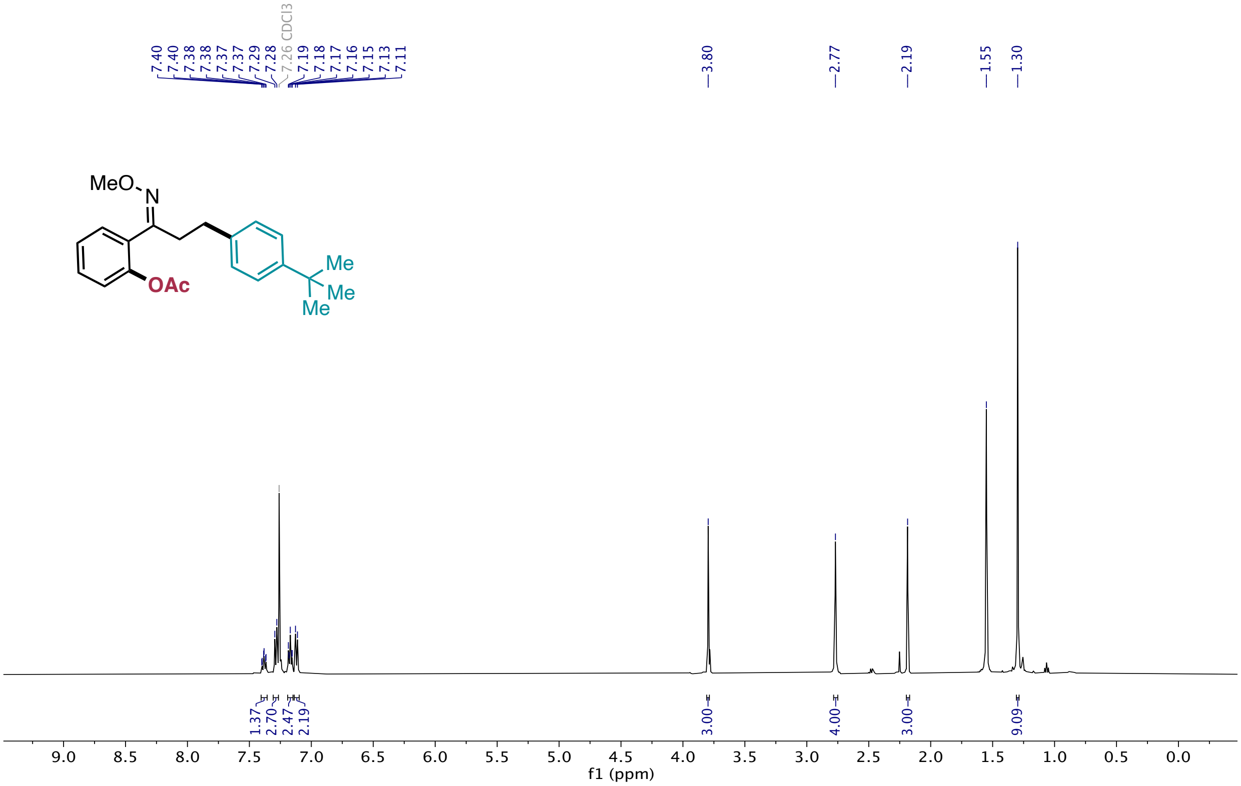

^{13}C NMR (126 MHz, $CDCl_3$) of (*Z*)-2-(3-(4-(*tert*-butyl)phenyl)-1-(methoxyimino)propyl)phenyl acetate (2w)

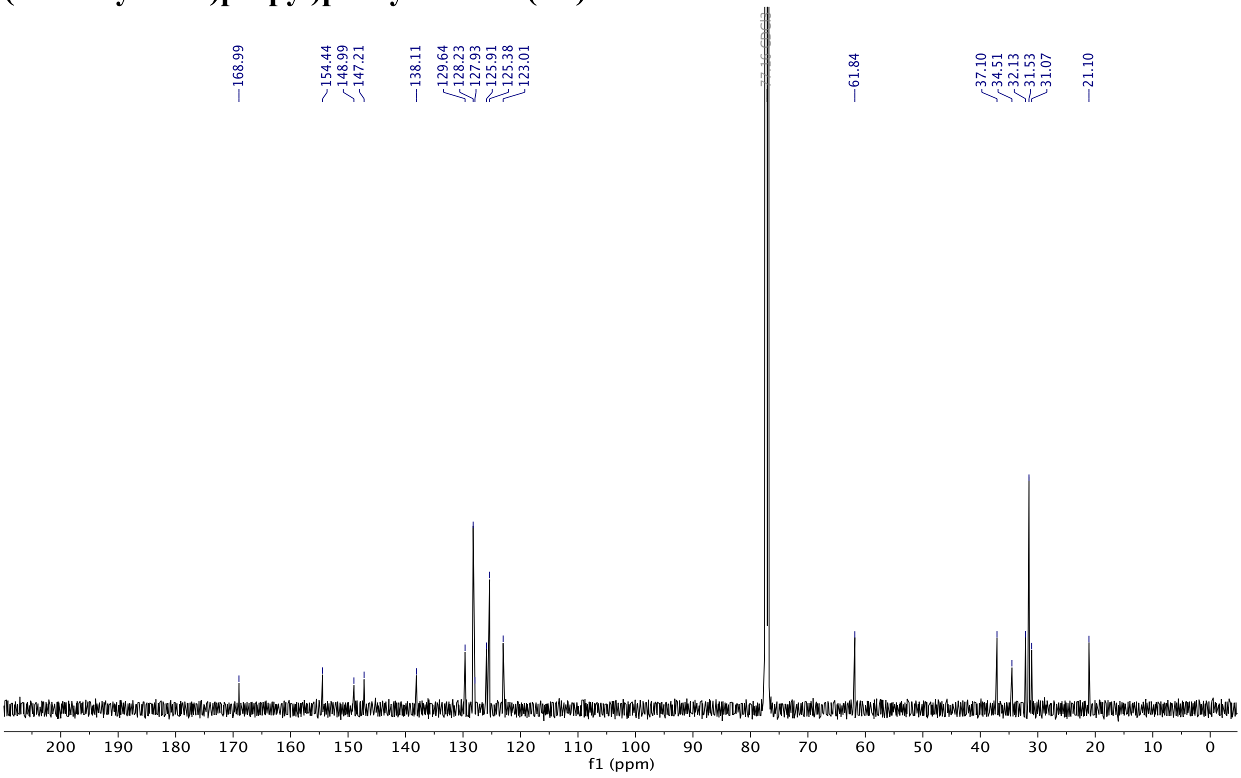

^{1}H NMR (500 MHz, $CDCl_3$) of (*Z*)-1-(2-methoxyphenyl)-3-(4-methoxyphenyl)propan-1-one *O*-methyl oxime (2x)

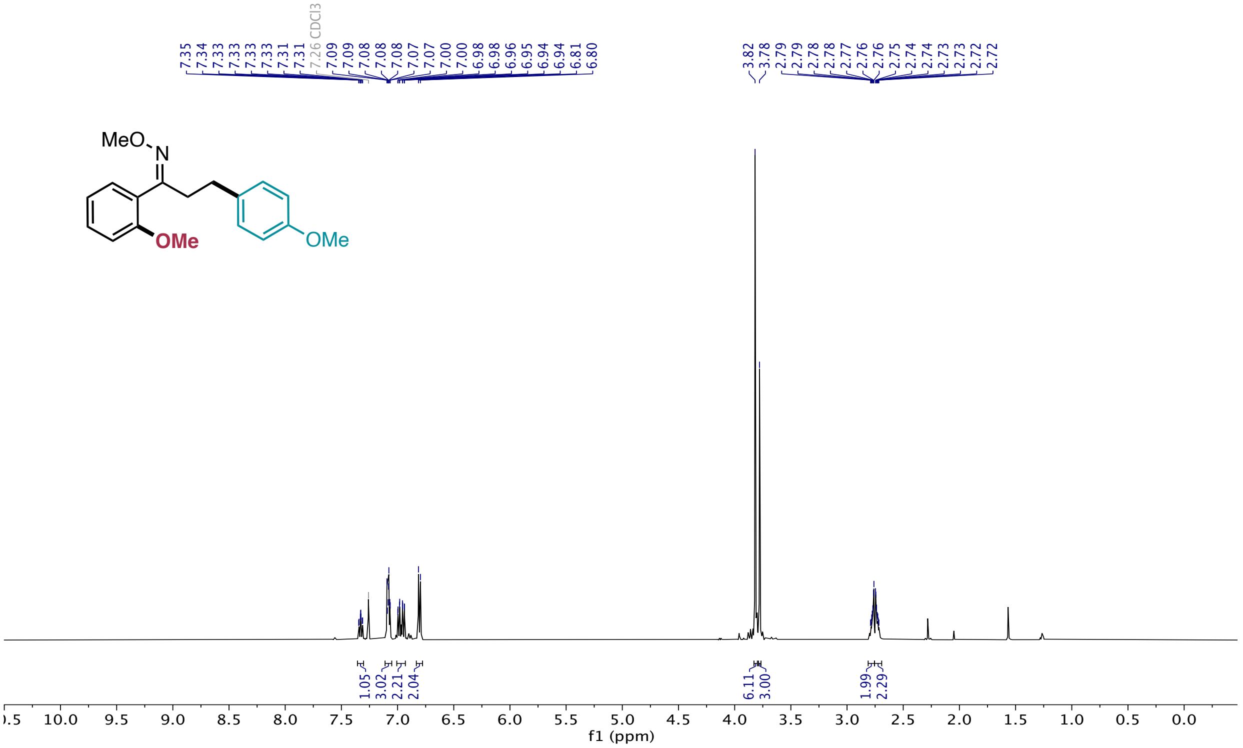

^{13}C{H} NMR (126 MHz, $CDCl_3$) of (*Z*)-1-(2-methoxyphenyl)-3-(4-methoxyphenyl)propan-1-one *O*-methyl oxime (2x)

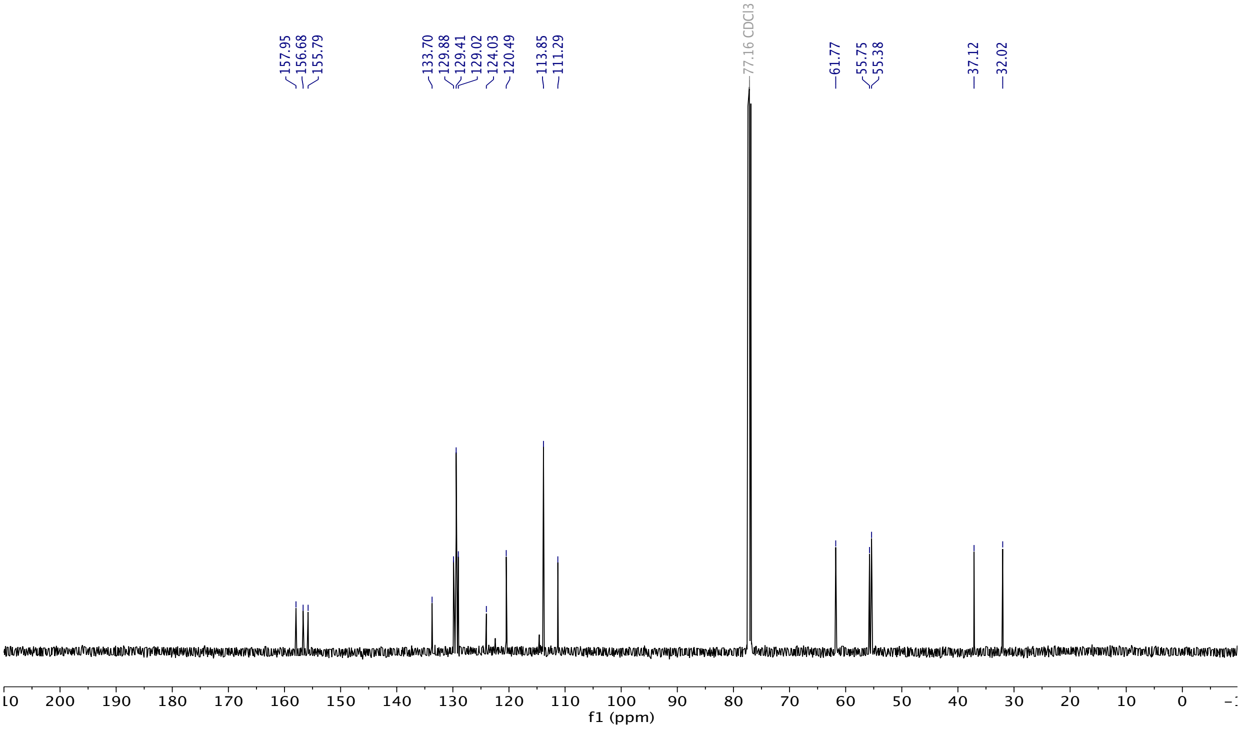

^{1}H NMR (500 MHz, CDCl$_3$) of (*Z*)-3-(4-(*tert*-butyl)phenyl)-1-(2-methoxyphenyl)propan-1-one *O*-methyl oxime (2y)

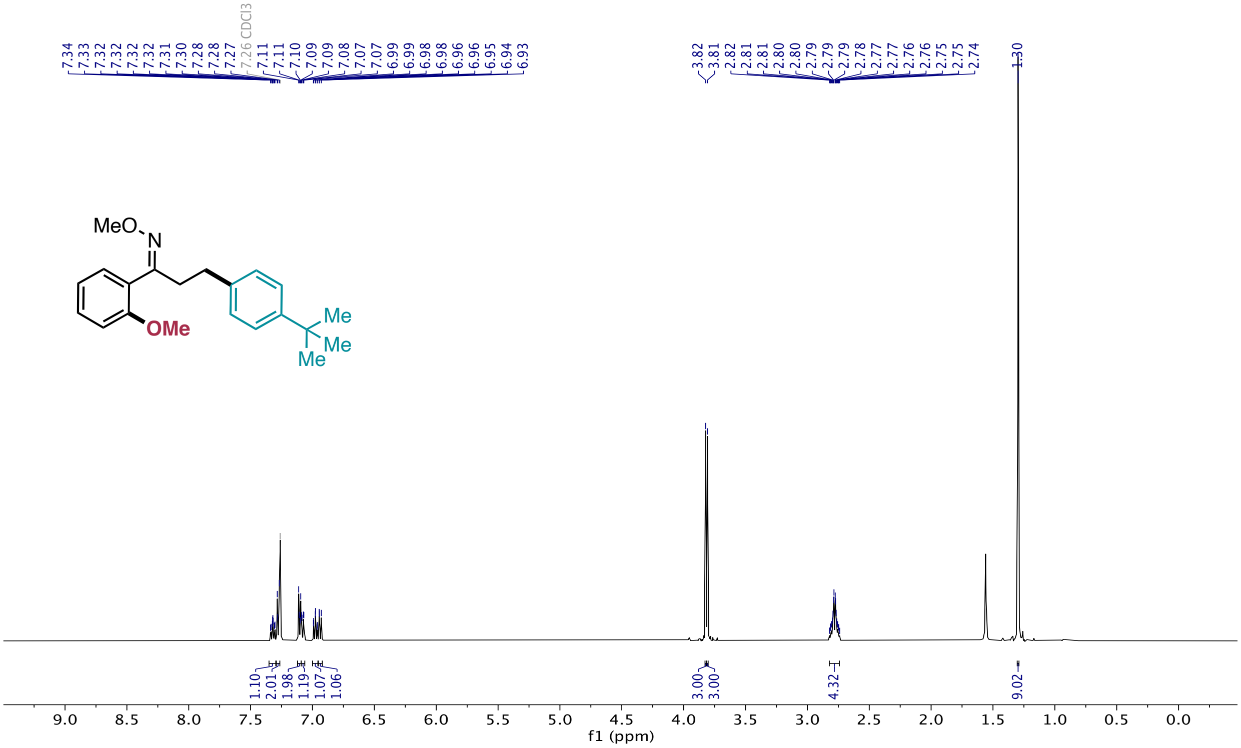

^{13}C NMR (151 MHz, CDCl$_3$) of (*Z*)-3-(4-(*tert*-butyl)phenyl)-1-(2-methoxyphenyl)propan-1-one *O*-methyl oxime (2y)

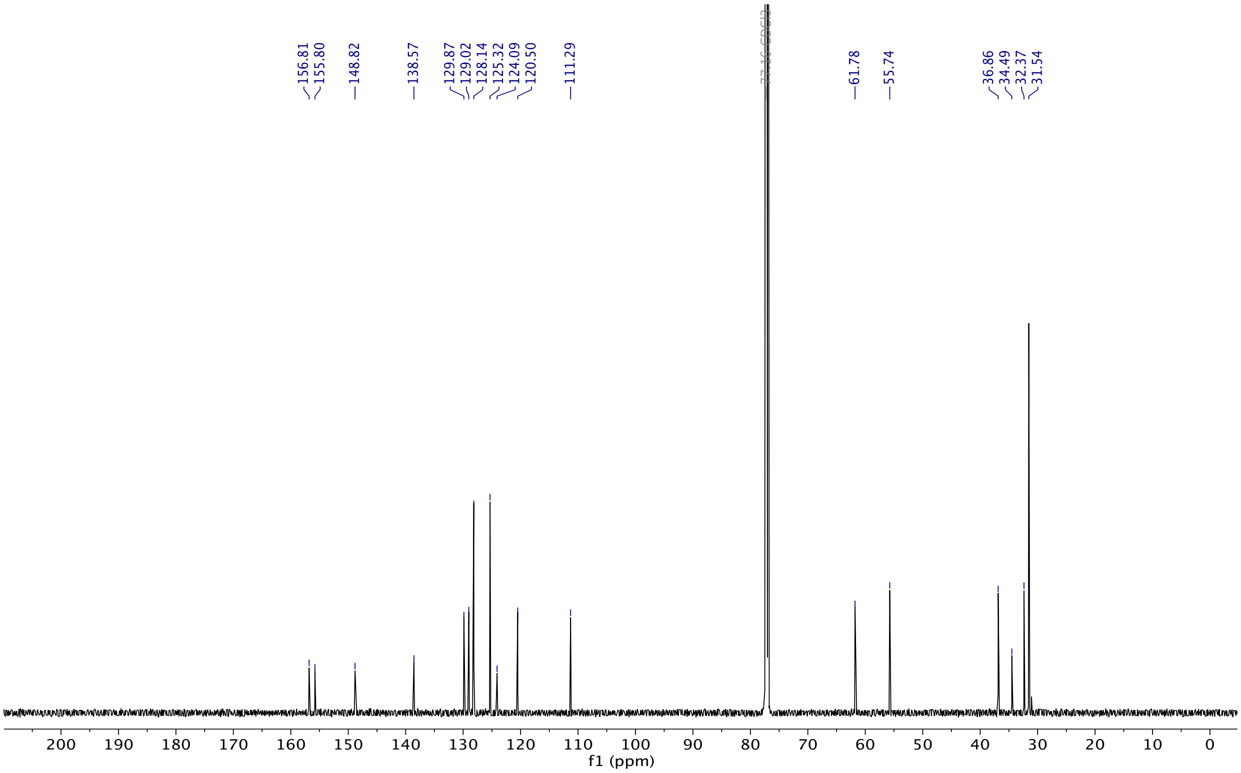

^{1}H NMR (600 MHz, CDCl$_3$) of (*Z*)-3-(4-ethylphenyl)-1-(2-methoxyphenyl)propan-1-one *O*-methyl oxime (2z)

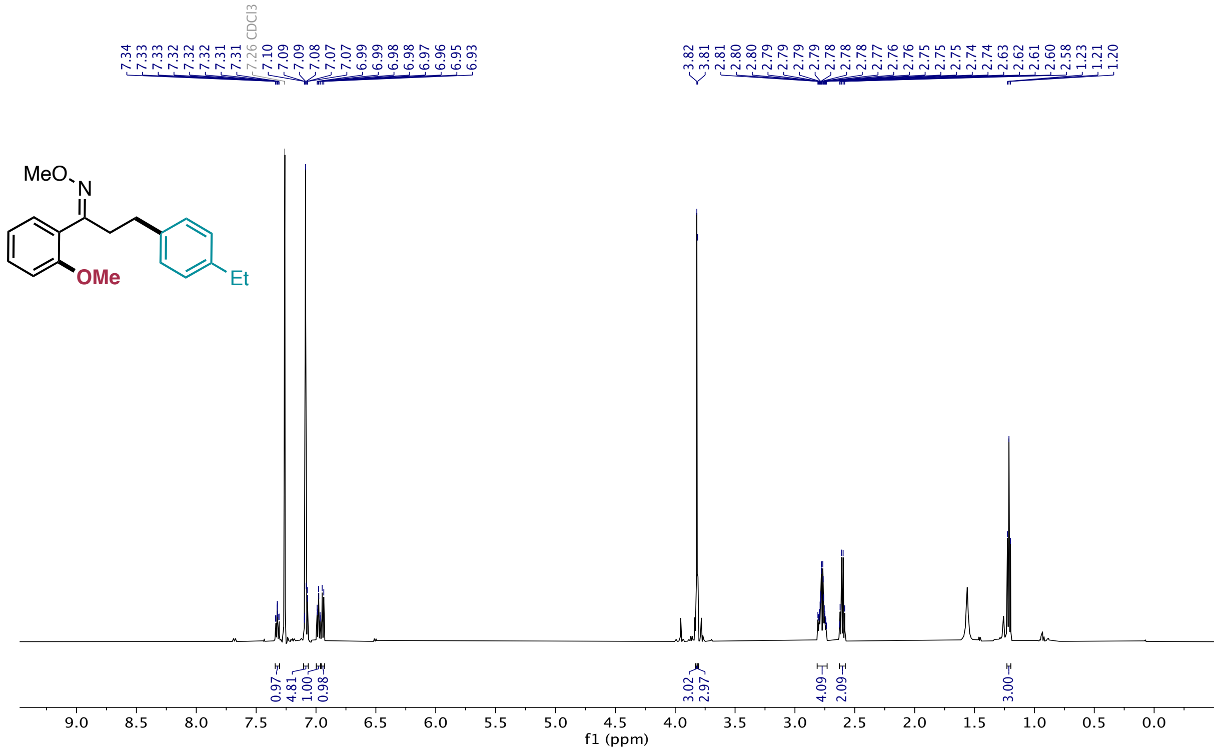

^{13}C NMR (151 MHz, CDCl$_3$) of (*Z*)-3-(4-ethylphenyl)-1-(2-methoxyphenyl)propan-1-one *O*-methyl oxime (2z)

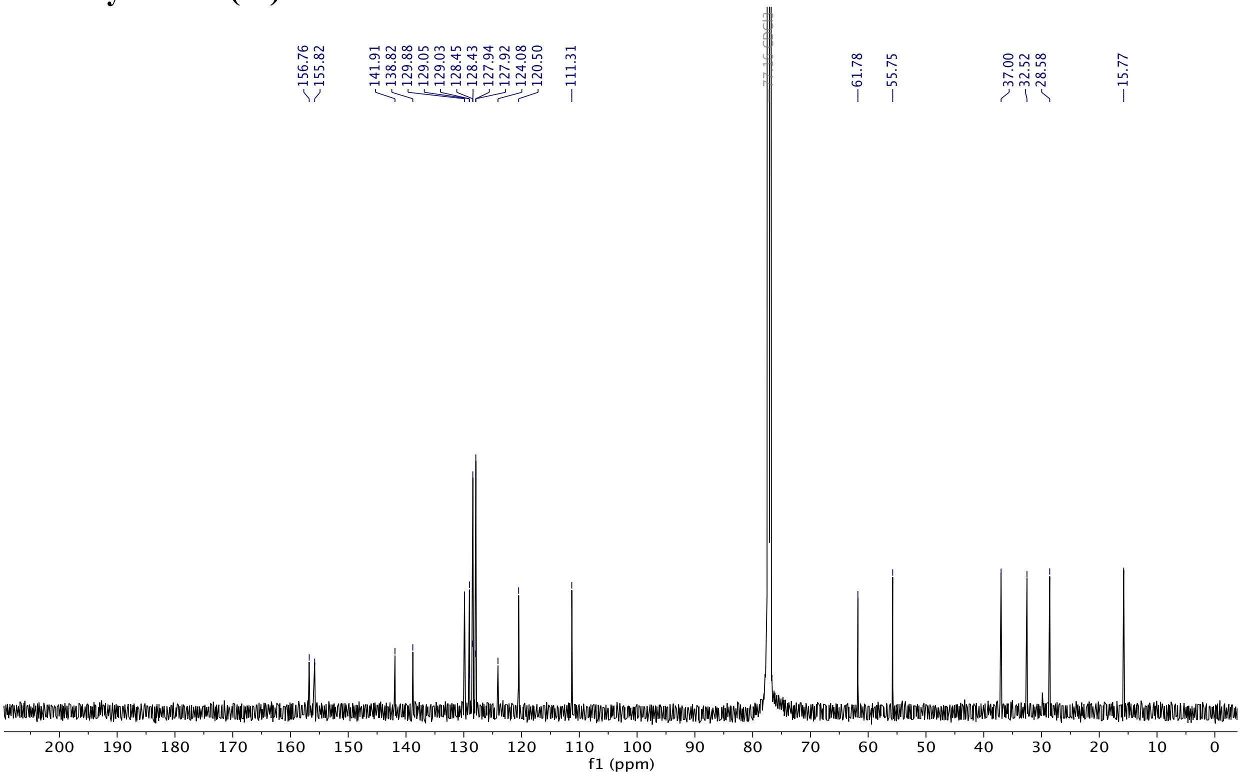

^{1}H NMR (500 MHz, CDCl$_3$) of (*Z*)-3-(4-fluorophenyl)-1-(2-methoxyphenyl)propan-1-one *O*-methyl oxime (2aa)

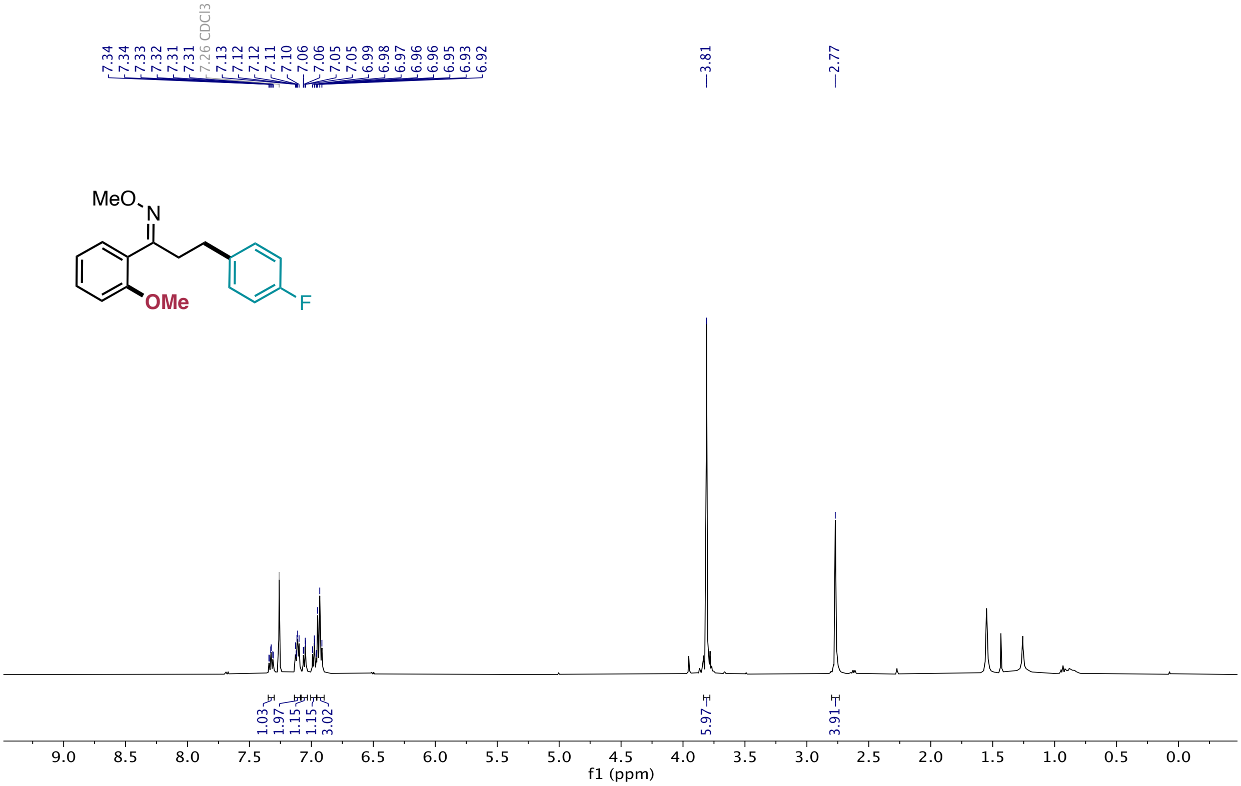

^{13}C NMR (126 MHz, CDCl$_3$) of (*Z*)-3-(4-fluorophenyl)-1-(2-methoxyphenyl)propan-1-one *O*-methyl oxime (2aa)

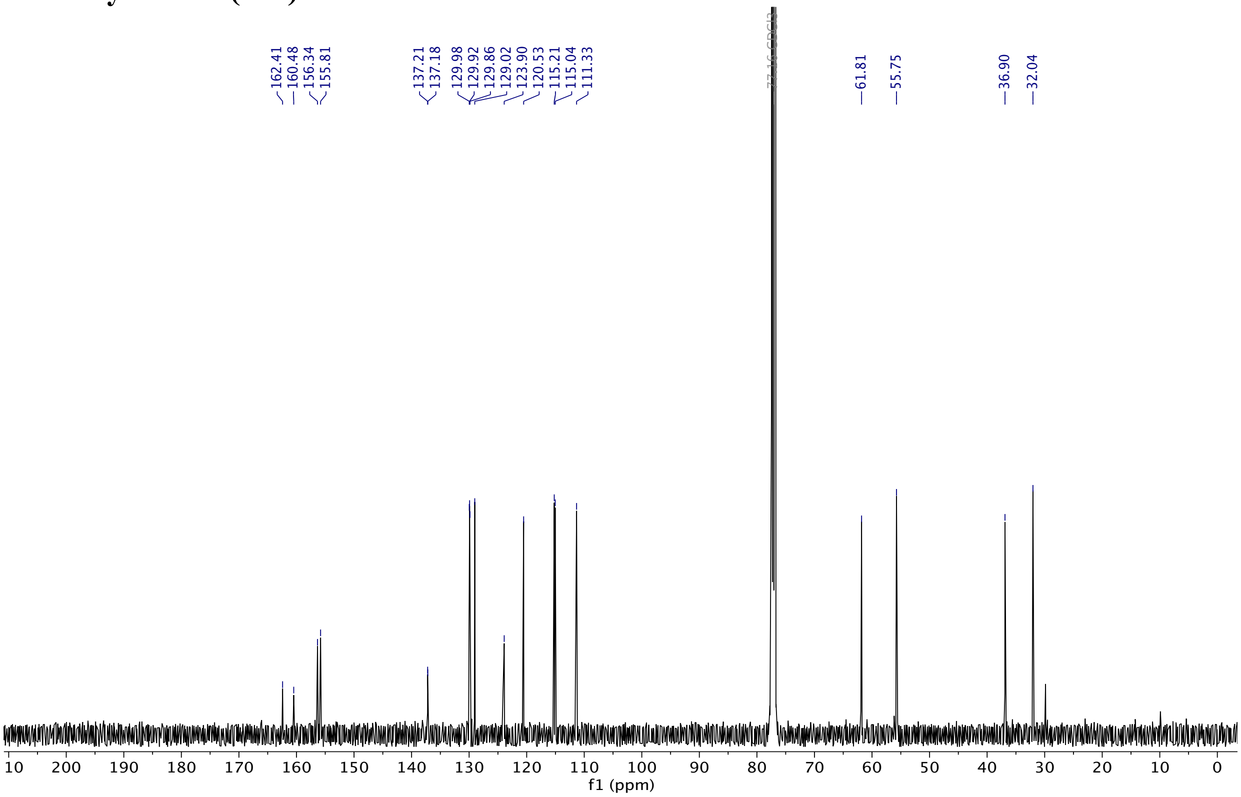

⁹F NMR (471 MHz, CDCl$_3$) of (*Z*)-3-(4-fluorophenyl)-1-(2-methoxyphenyl)propan-1-one *O*-methyl oxime (2aa)

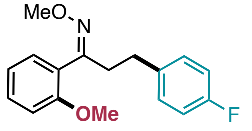

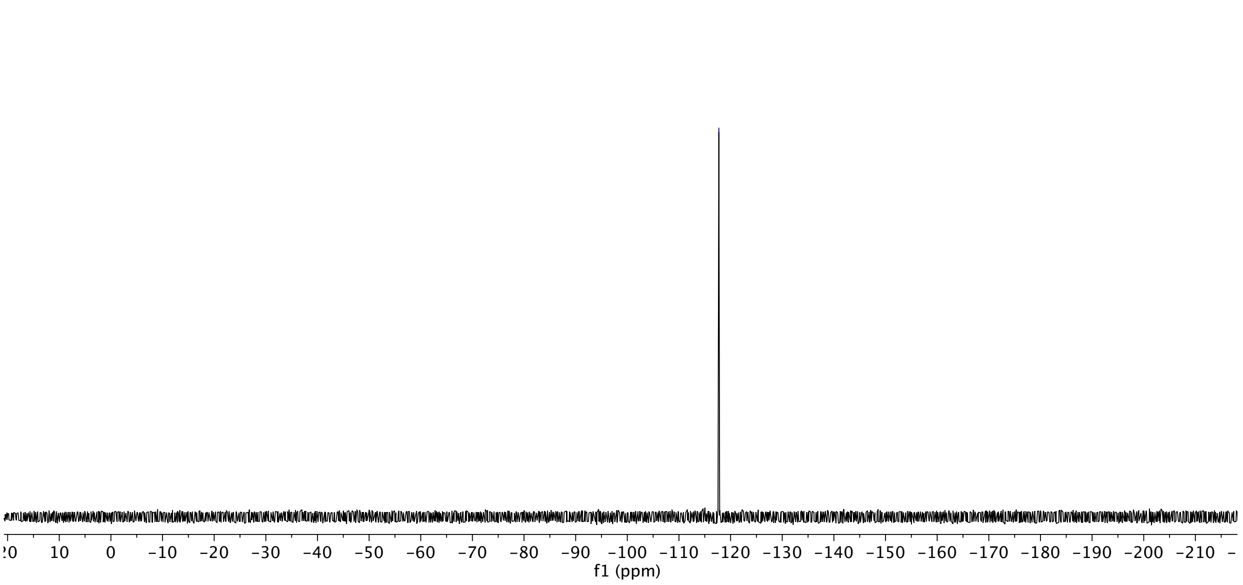

^{1}H NMR (600 MHz, CDCl$_3$) of (*Z*)-3-(3-chlorophenyl)-1-(2-methoxyphenyl)propan-1-one *O*-methyl oxime (2ab)

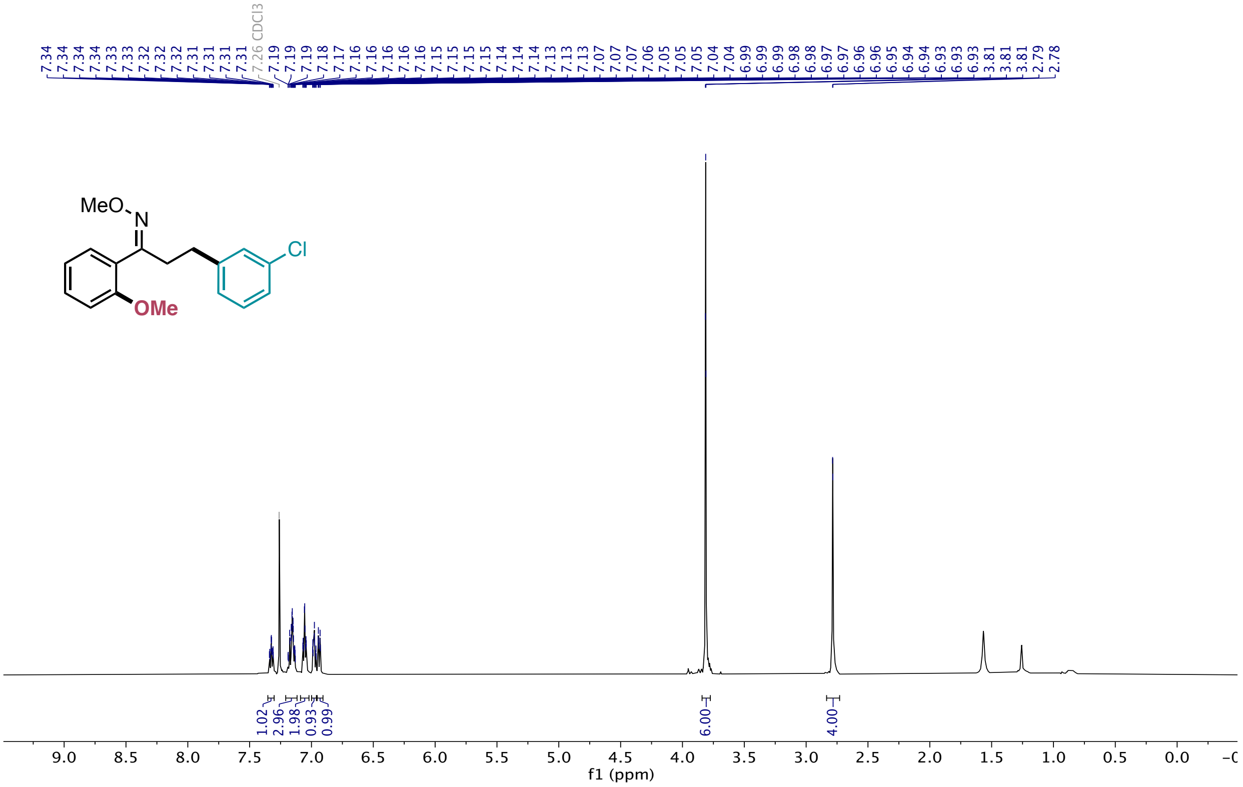

^{13}C NMR (151 MHz, CDCl$_3$) of (*Z*)-3-(3-chlorophenyl)-1-(2-methoxyphenyl)propan-1-one *O*-methyl oxime (2ab)

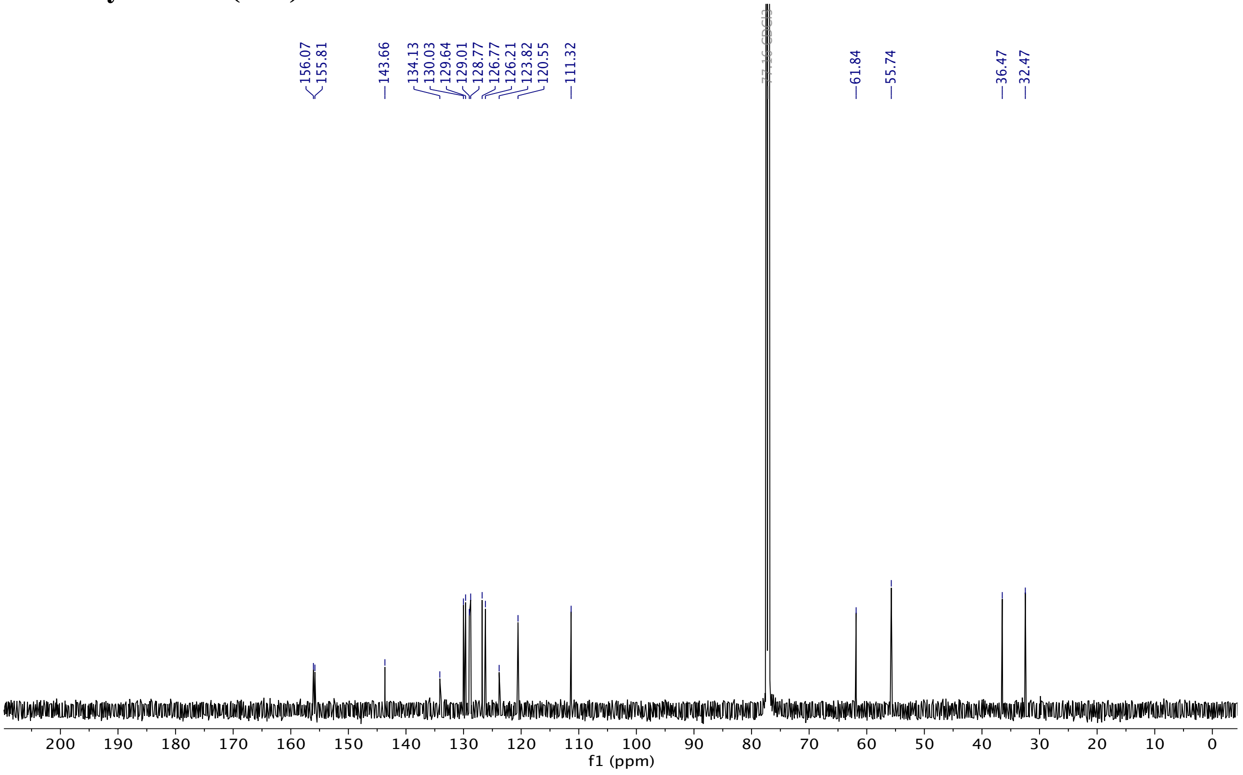

^{1}H NMR (500 MHz, $CDCl_3$) of (Z)-1-([1,1'-biphenyl]-2-yl)-3-phenylpropan-1-one O-methyl oxime (2ac)

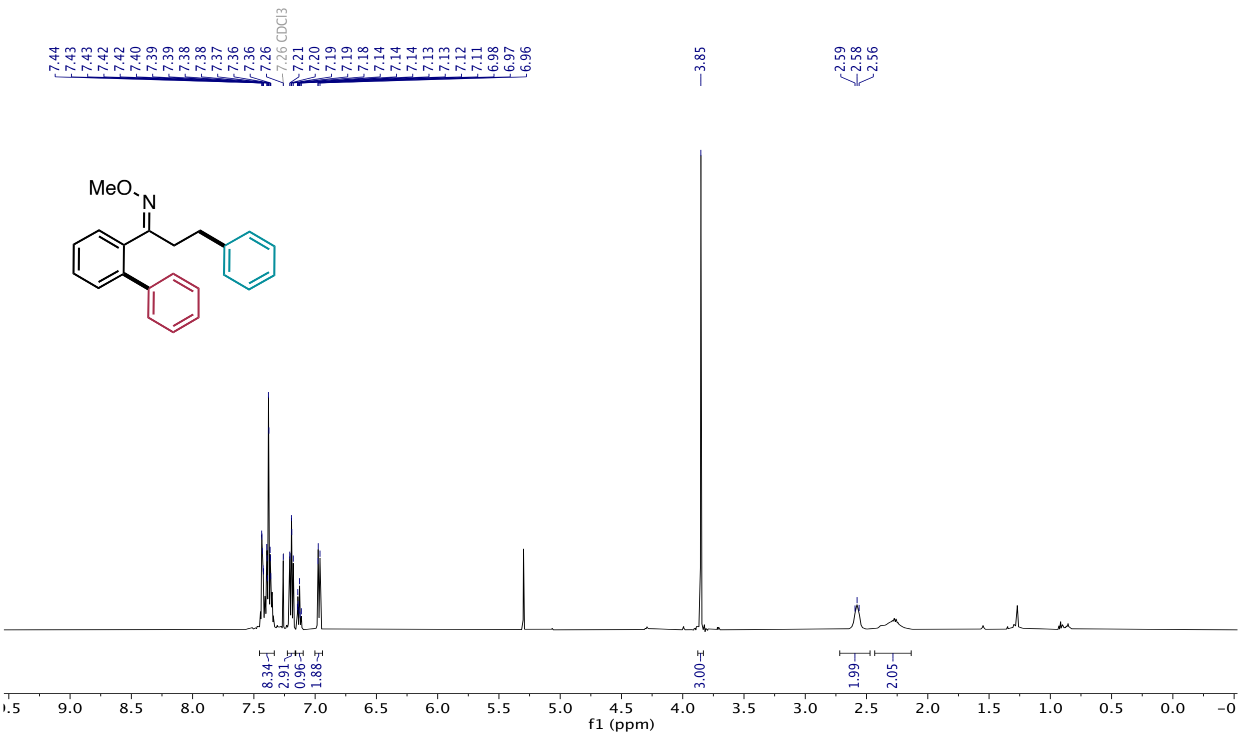

^{13}C{H} NMR (126 MHz, $CDCl_3$) of (Z)-1-([1,1'-biphenyl]-2-yl)-3-phenylpropan-1-one O-methyl oxime (2ac)

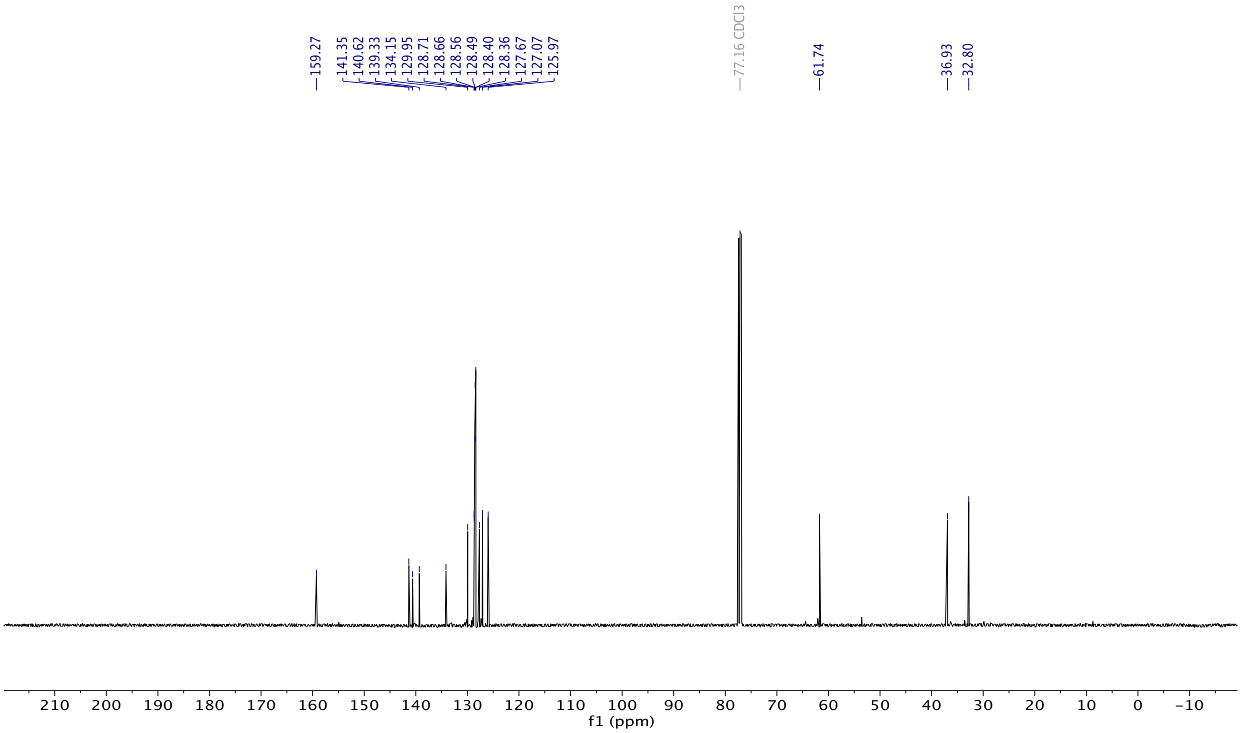

^{1}H NMR (500 MHz, CDCl$_3$) of (Z)-2-(4-(4-fluorophenyl)-2-(methoxyimino)butanoyl)phenyl acetate (10)

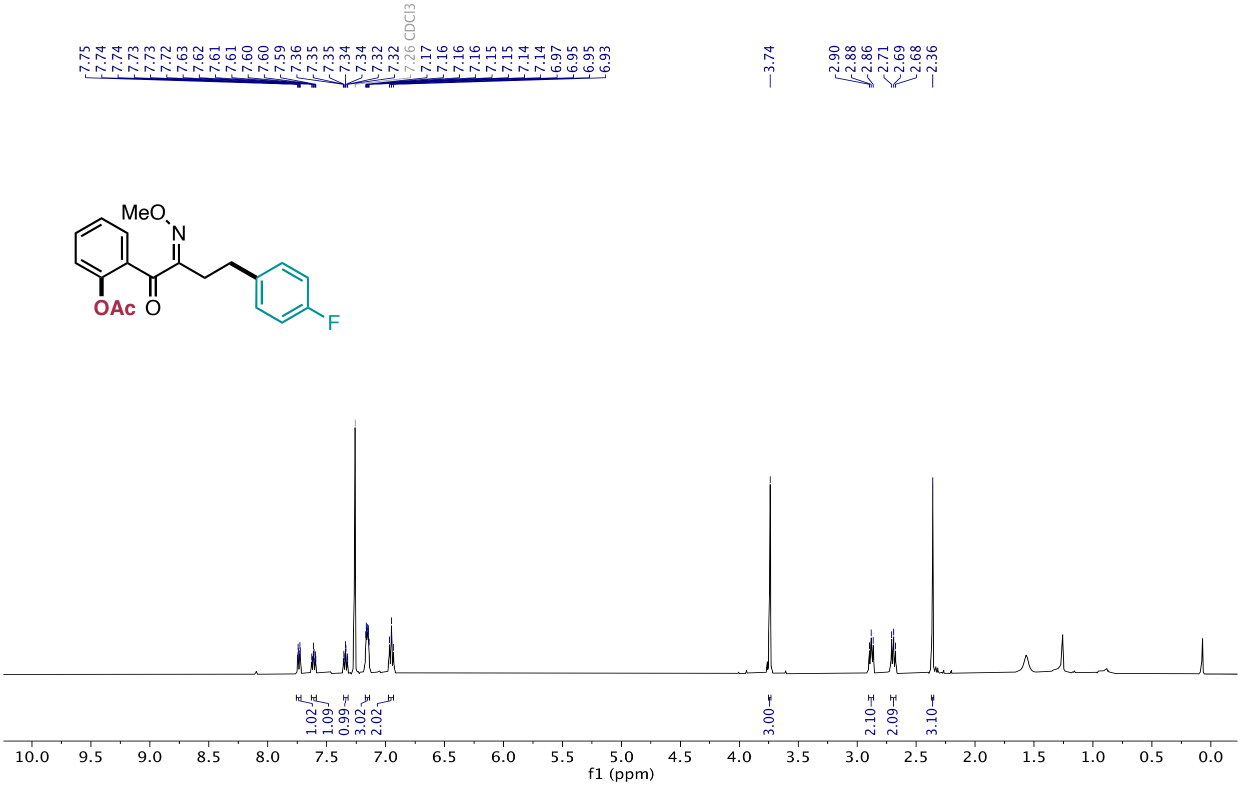

^{13}C NMR (126 MHz, CDCl$_3$) of (Z)-2-(4-(4-fluorophenyl)-2-(methoxyimino)butanoyl)phenyl acetate (10)

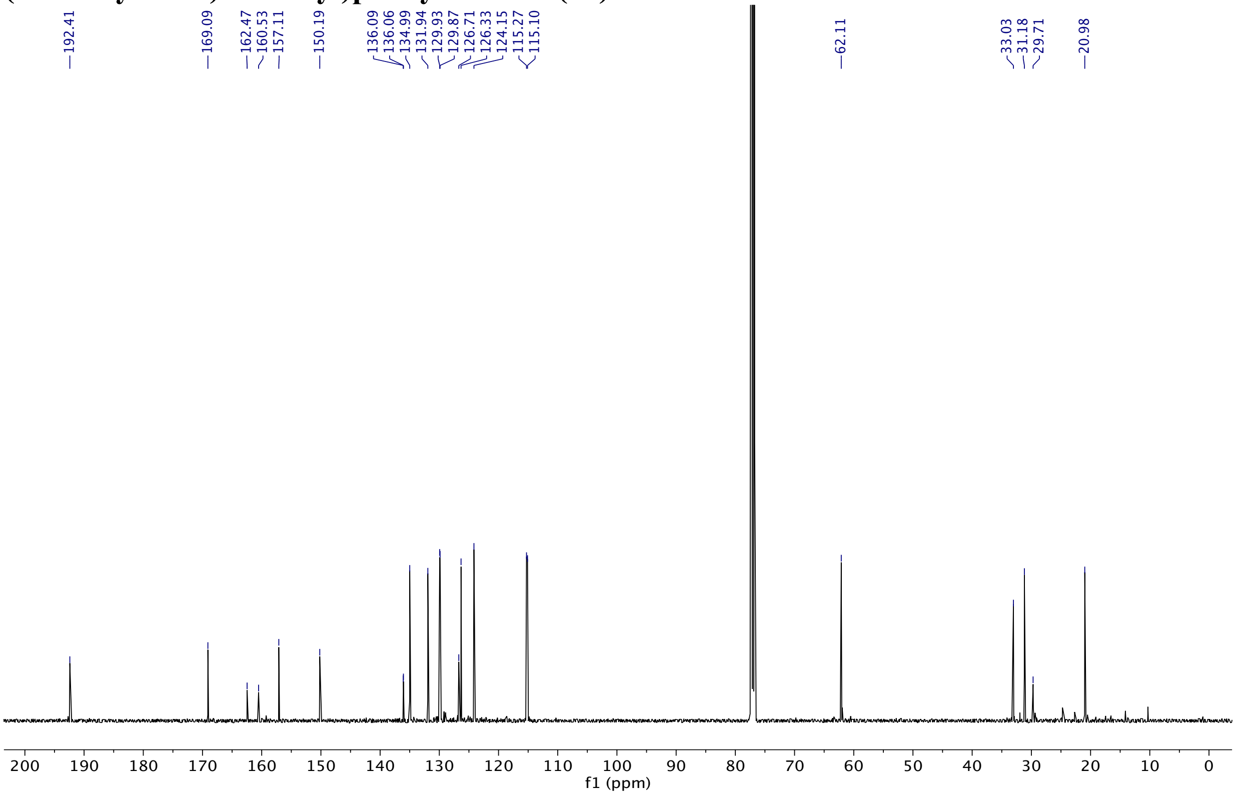

^{9}F NMR (471 MHz, $CDCl_3$) of (Z)-2-(4-(4-fluorophenyl)-2-(methoxyimino)butanoyl)phenyl acetate (10)

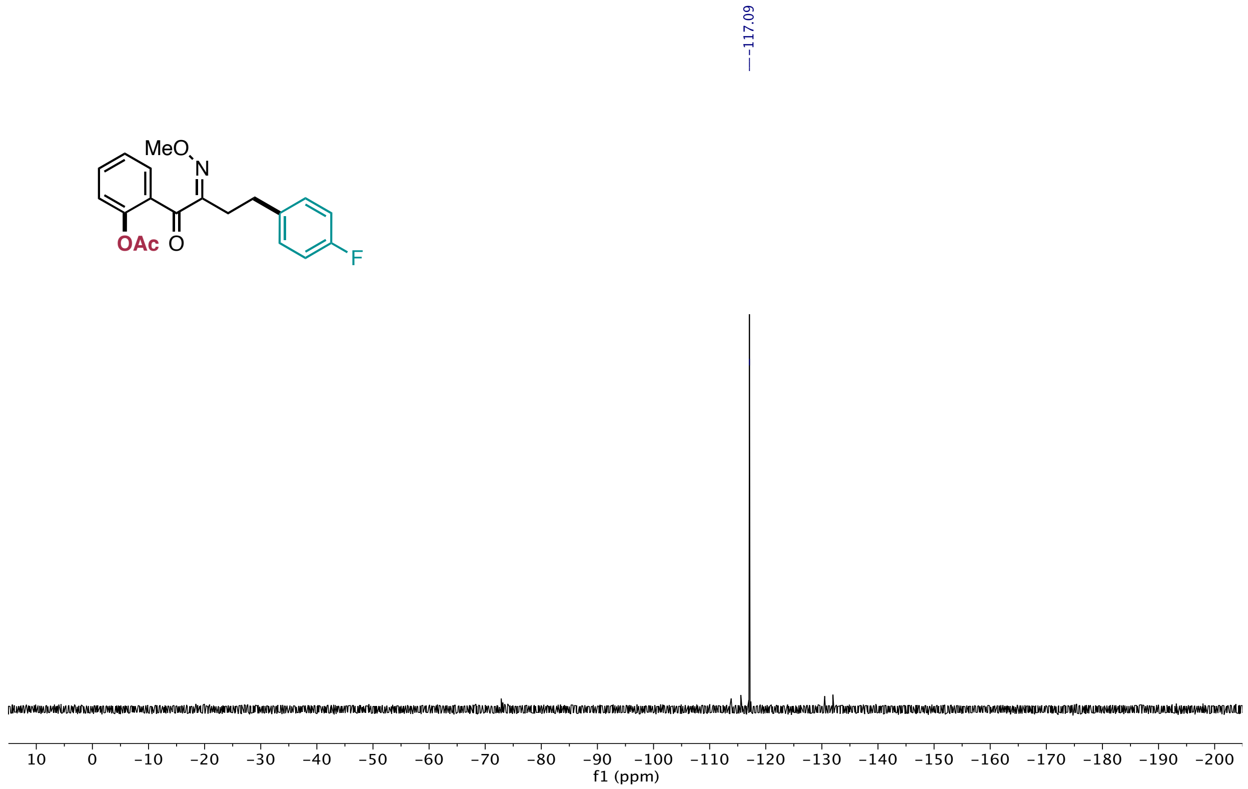

^{1}H NMR (600 MHz, $CDCl_3$) of 2-Methoxy-*N*-(3-phenylpropyl)aniline (11)

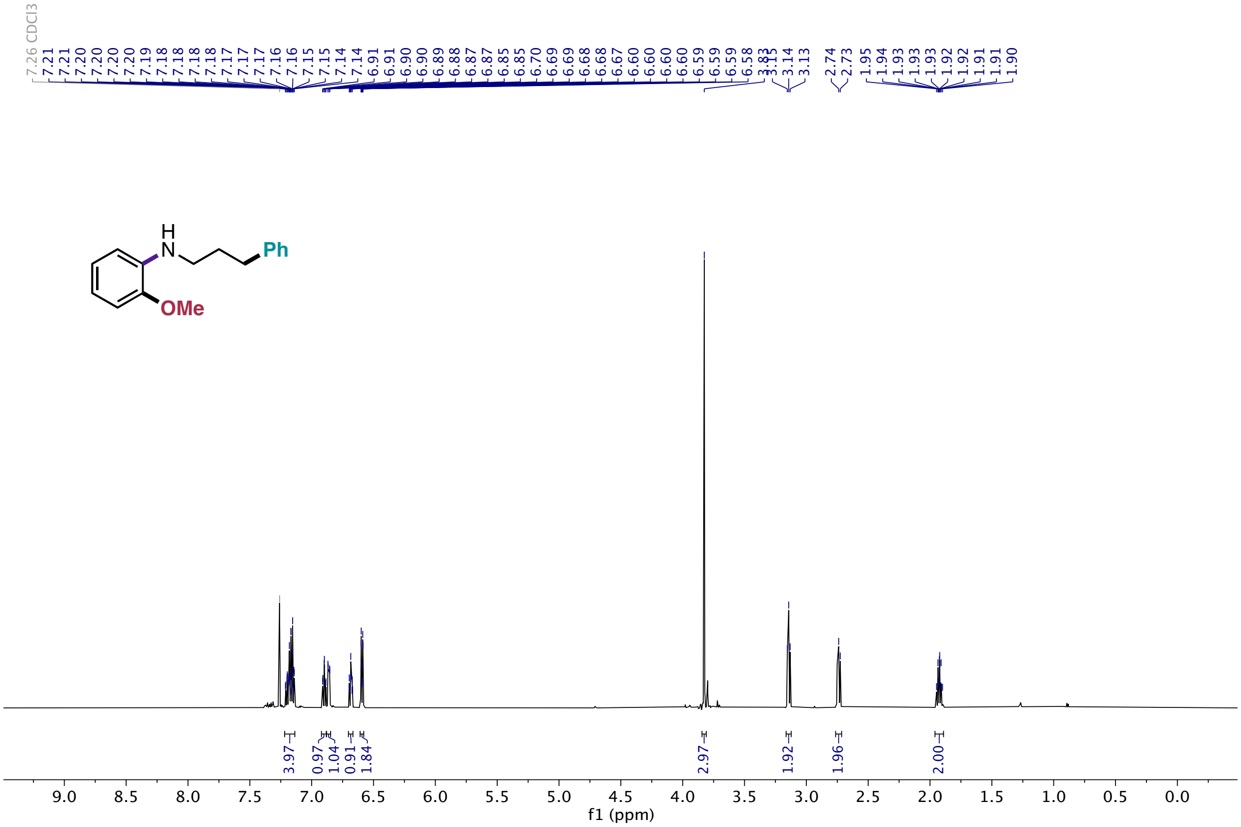

^{13}C NMR (151 MHz, $CDCl_3$) of 2-Methoxy-*N*-(3-phenylpropyl)aniline (11)

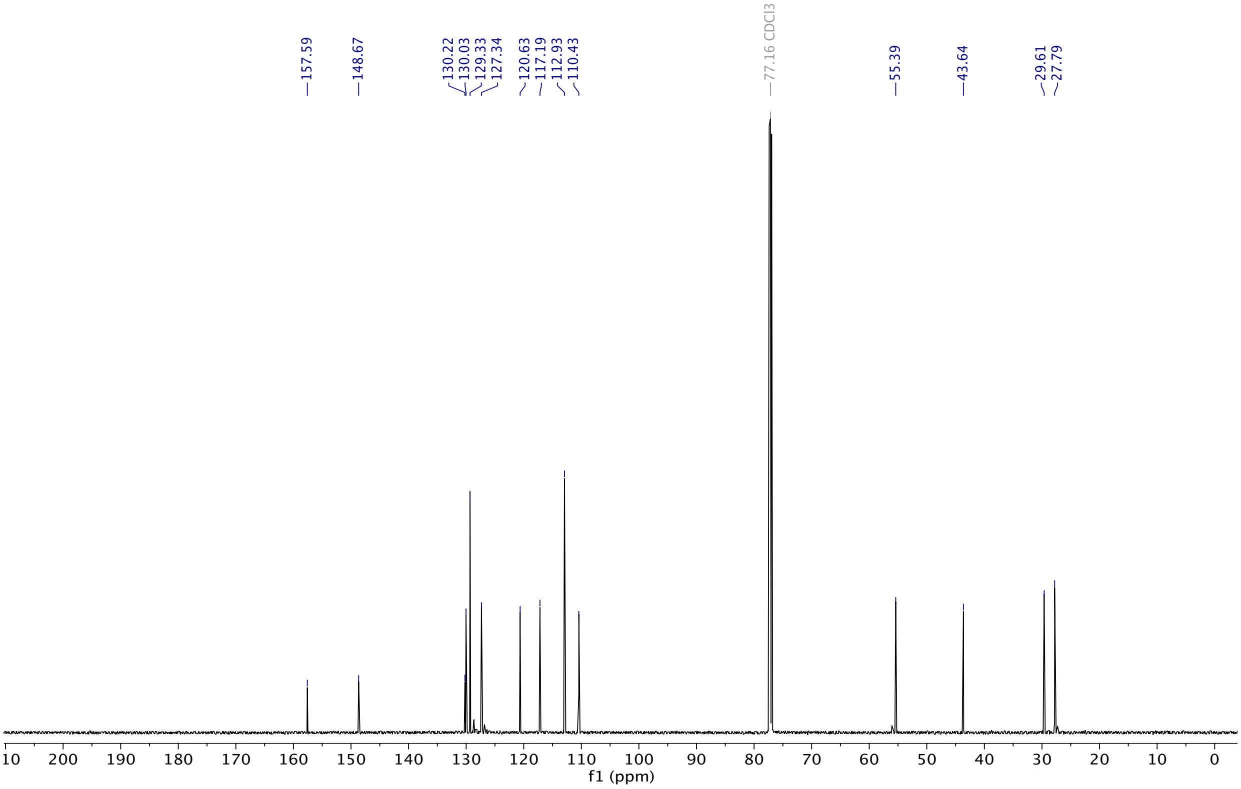

^{1}H NMR (400 MHz, CDCl$_3$) of 1-(2-methoxyphenyl)-3-phenylpropan-1-one (12)

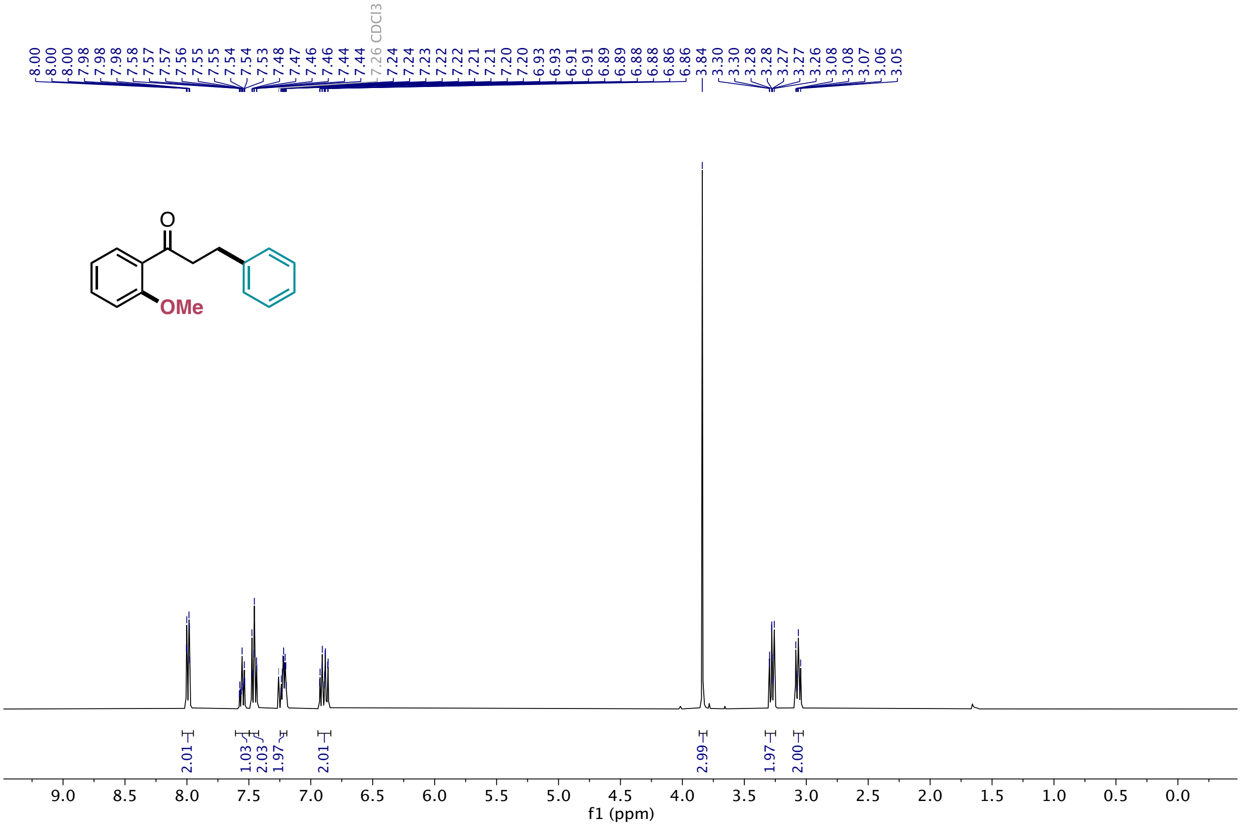

^{13}C NMR (101 MHz, CDCl$_3$) of 1-(2-methoxyphenyl)-3-phenylpropan-1-one (12)

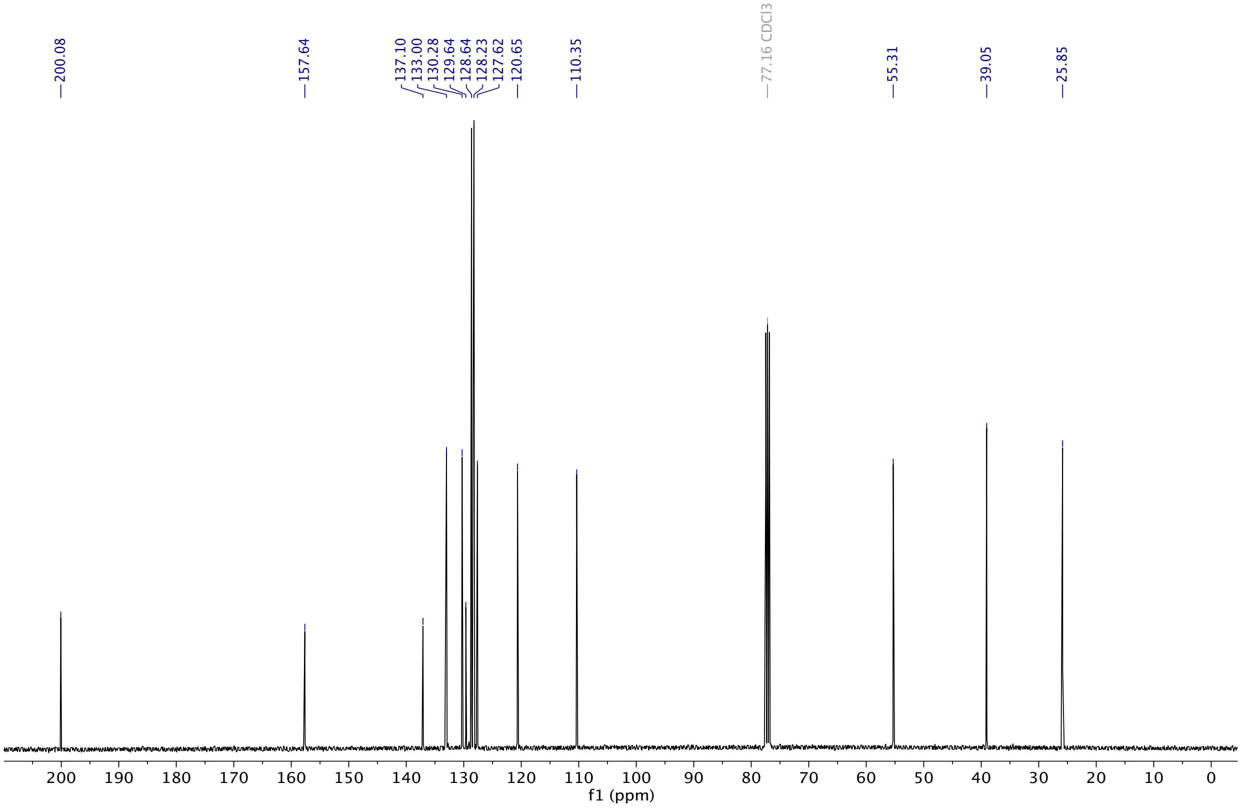

^{1}H NMR (400 MHz, $CDCl_3$ of 1-(2-hydroxyphenyl)-3-phenylpropan-1-one (14)

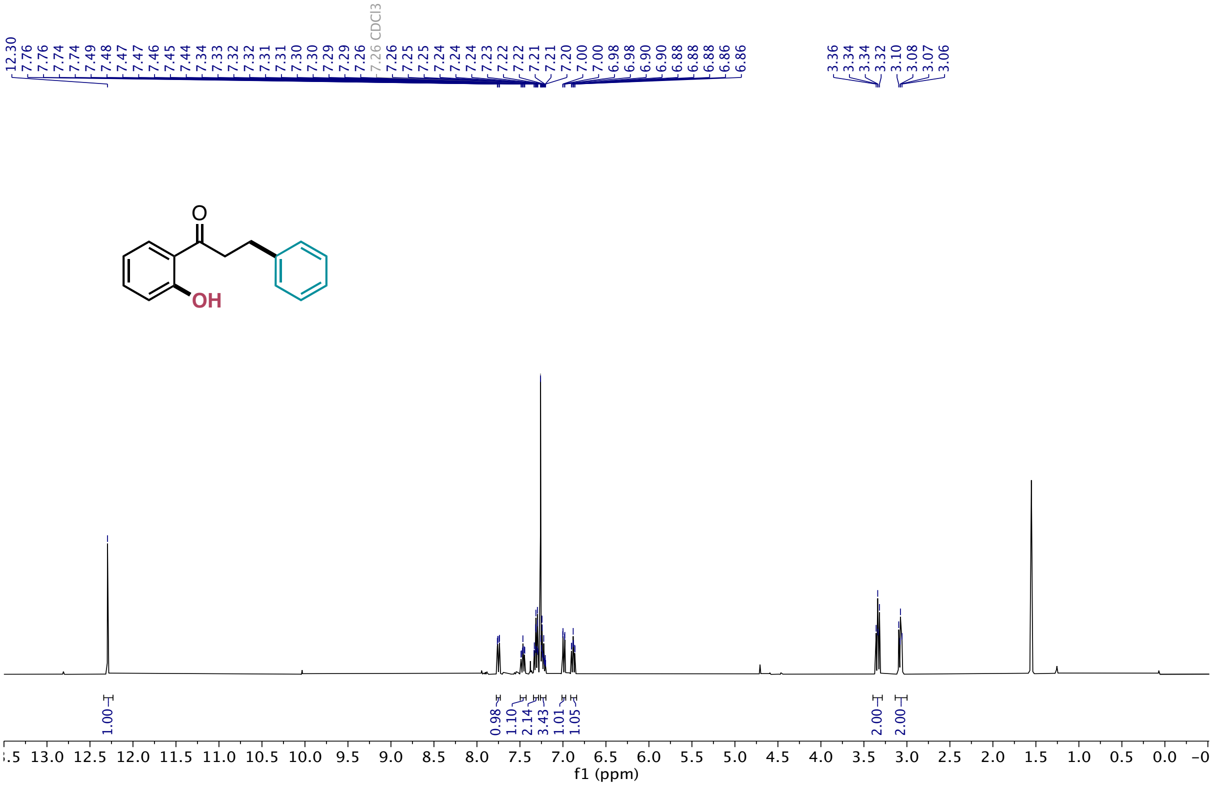

^{13}C NMR (101 MHz, $CDCl_3$) of 1-(2-hydroxyphenyl)-3-phenylpropan-1-one

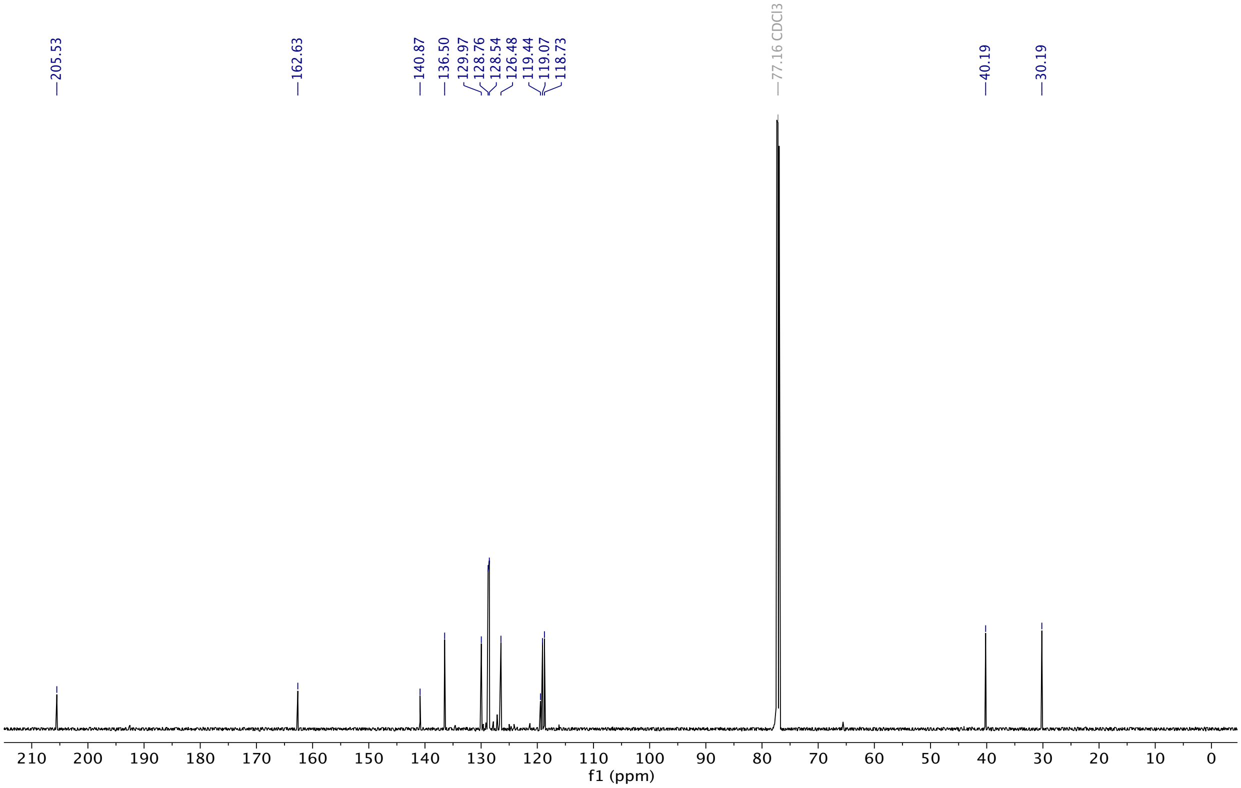

^{1}H NMR (400 MHz, $CDCl_3$) of 1-(2-(oxiran-2-ylmethoxy)phenyl)-3-phenylpropan-1-one

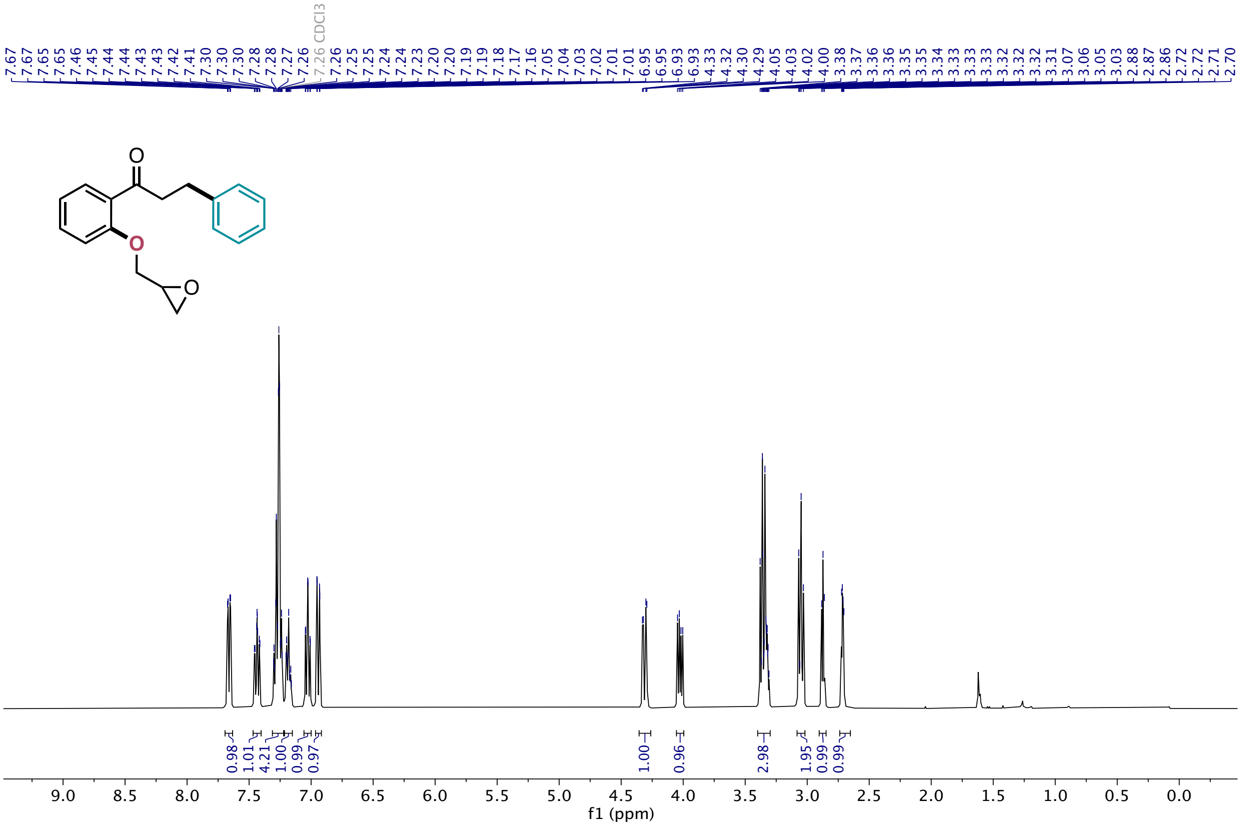

^{13}C NMR (101 MHz, $CDCl_3$ of 1-(2-(oxiran-2-ylmethoxy)phenyl)-3-phenylpropan-1-one

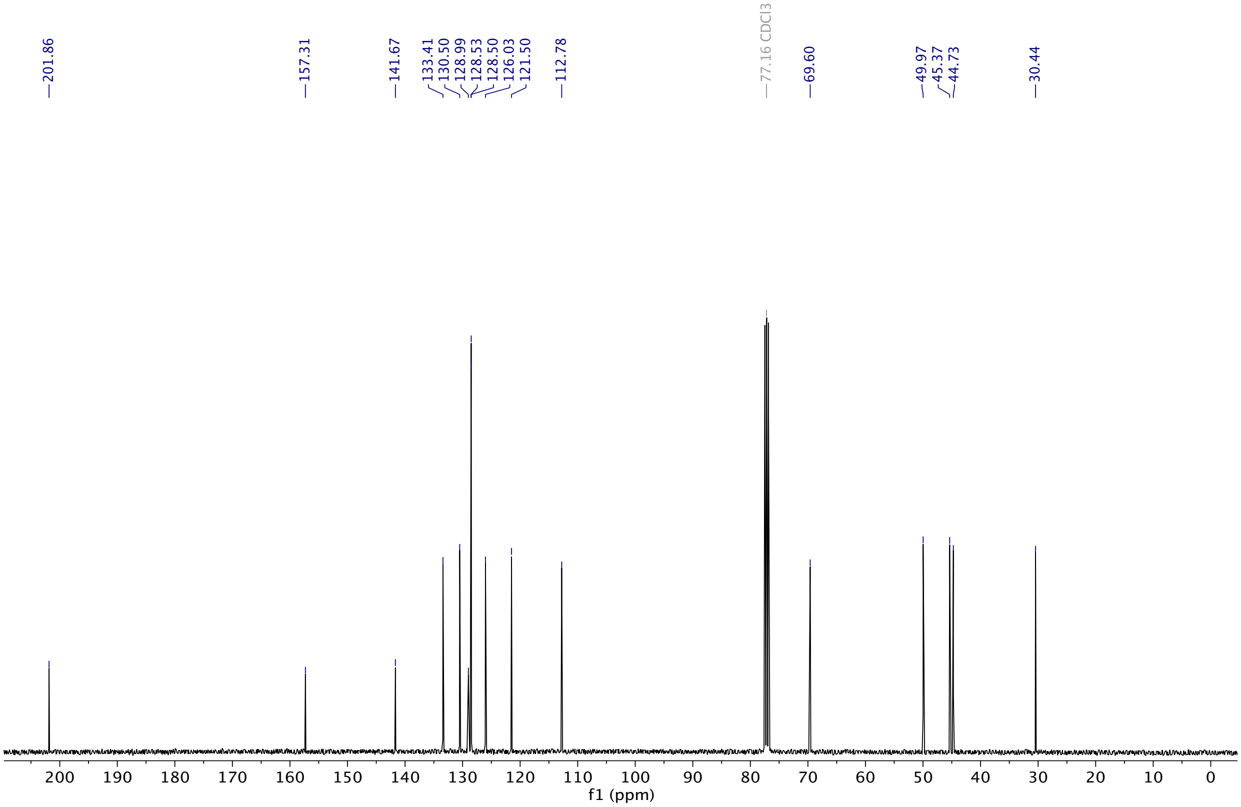

^{1}H NMR (600 MHz, $CDCl_3$) of 1-(2-(2-hydroxy-3-(propylamino)propoxy)phenyl)-3-phenylpropan-1-one (15)

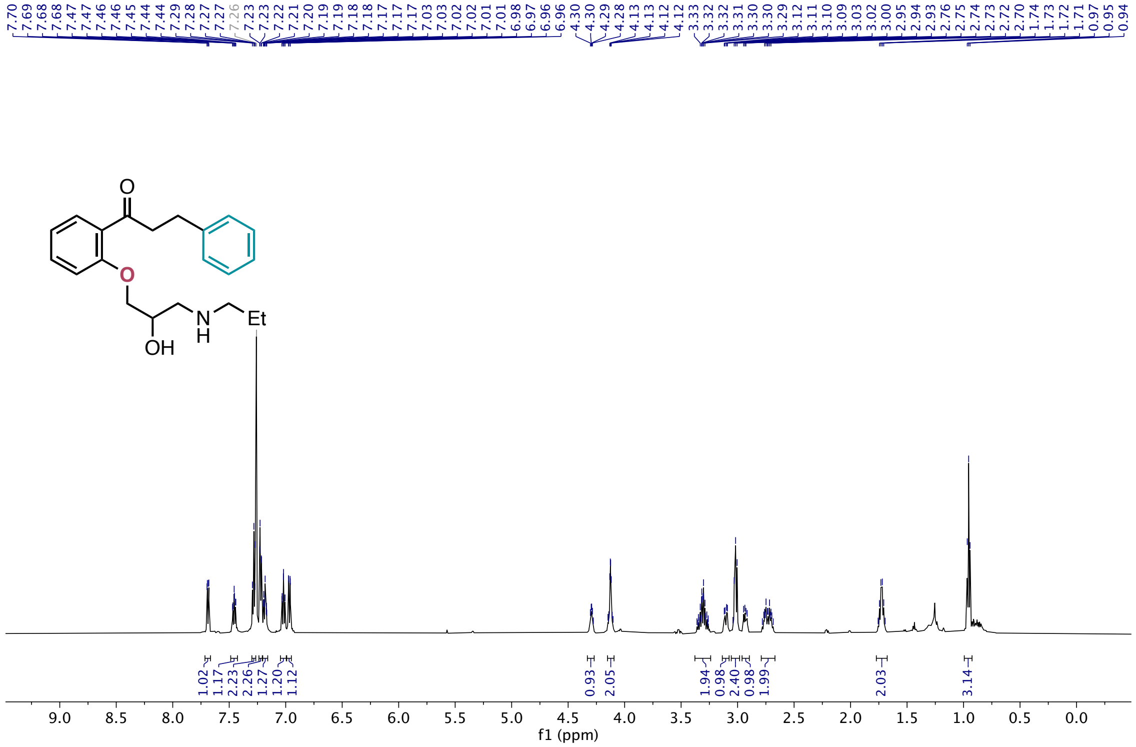

^{13}C NMR (151 MHz, $CDCl_3$) of 1-(2-(2-hydroxy-3-(propylamino)propoxy)phenyl)-3-phenylpropan-1-one (15)

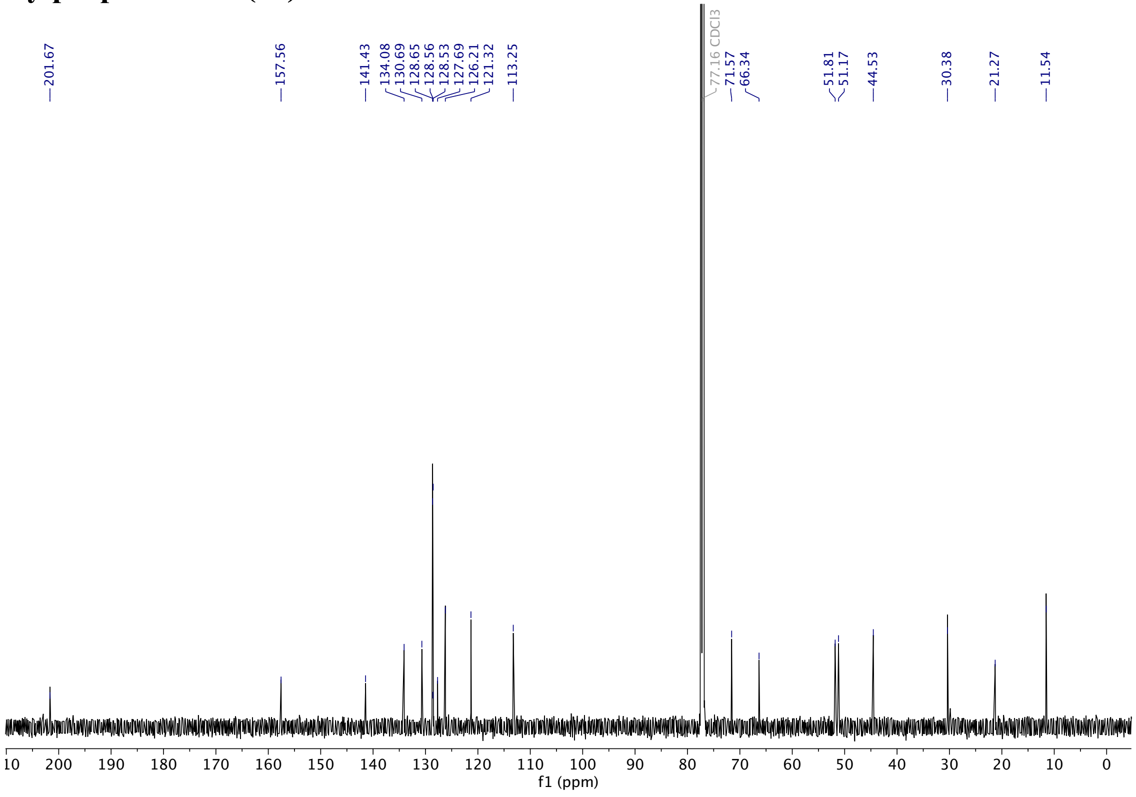

^{1}H NMR (Crude) (500 MHz, $CDCl_3$) of (*Z*)-2-(1-(methoxyimino)-2-methyl-3-phenylpropyl)phenyl acetate (2l)

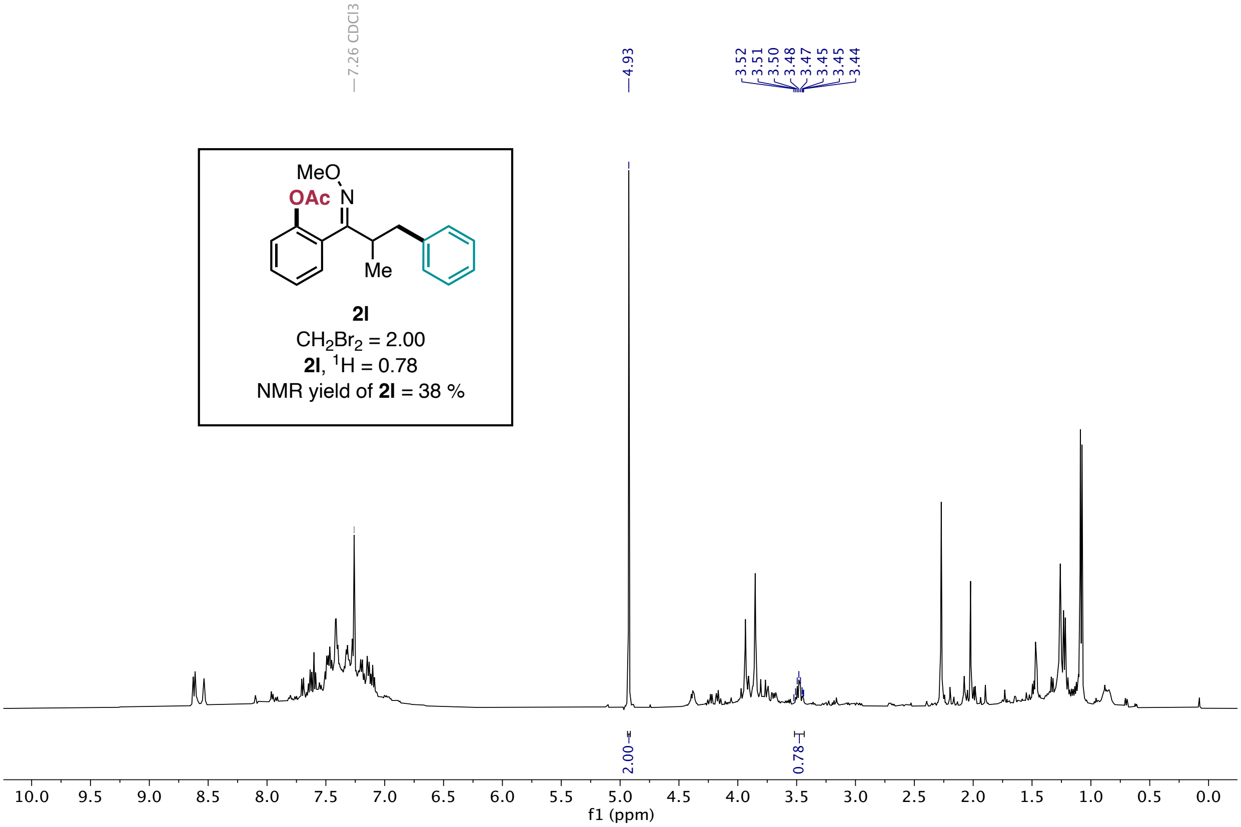

^{1}H NMR (Crude) (500 MHz, $CDCl_3$) of (*Z*)-2-(1-(methoxyimino)-2,3-diphenylpropyl)phenyl acetate (2m)

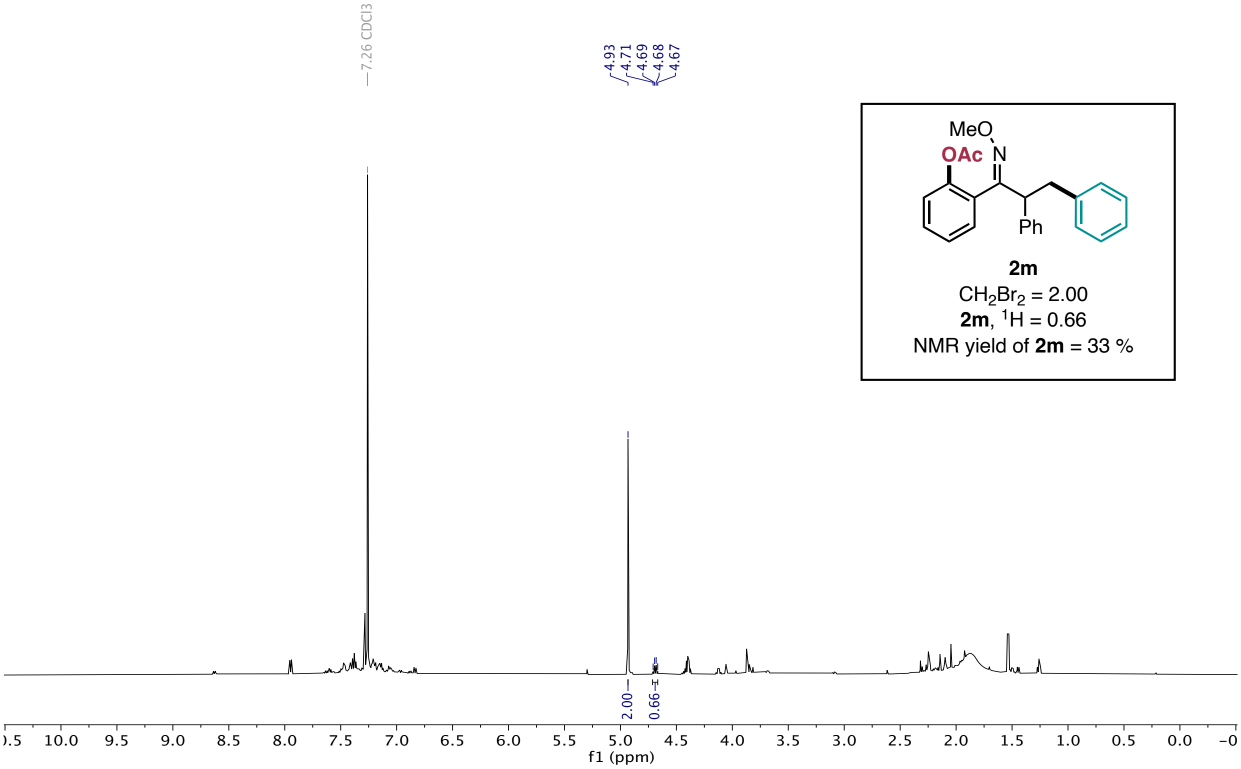

^{1}H NMR (Crude) (500 MHz, $CDCl_3$) of (*Z*)-3-(1-(methoxyimino)-3-phenylpropyl)benzo[*b*]thiophen-4-yl acetate (2o)

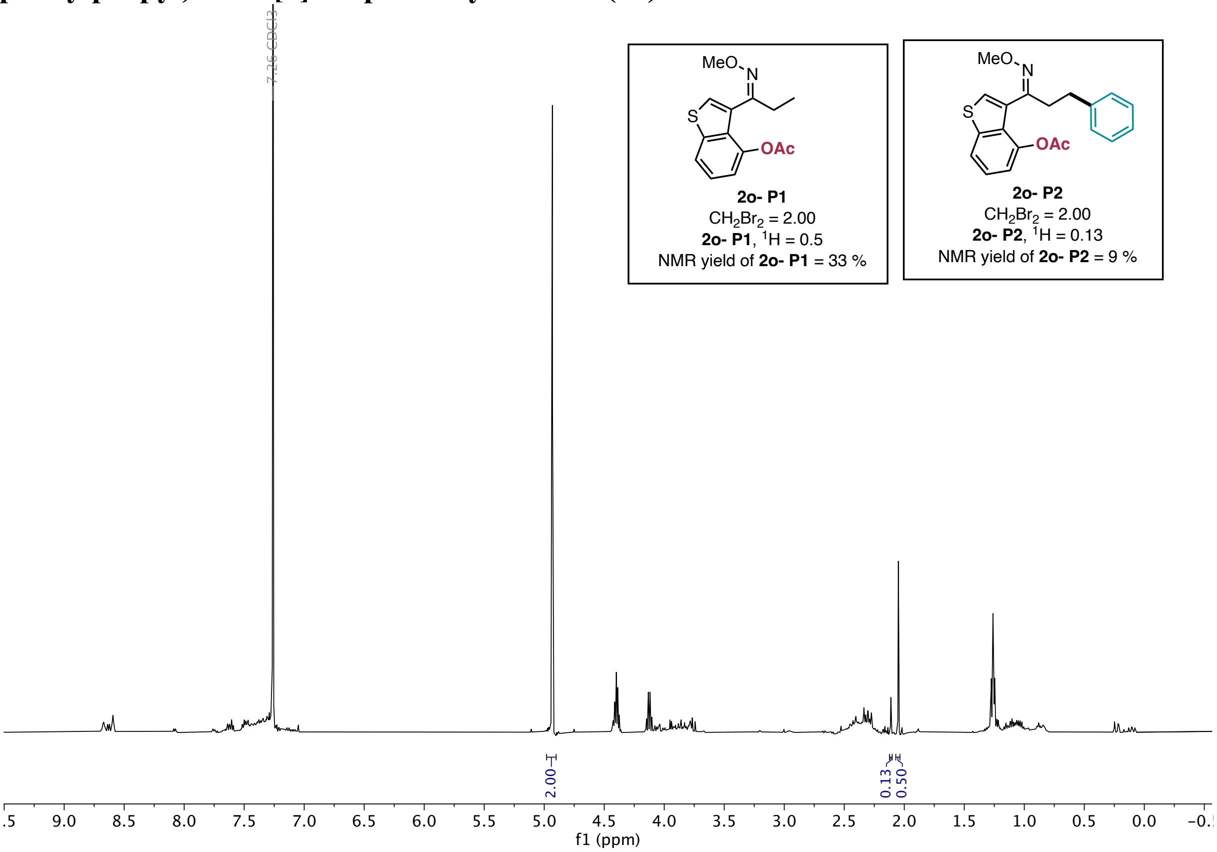

^{1}H NMR (Crude) (500 MHz, $CDCl_3$) of (*E*)-2-((methoxyimino)((1-phenylpropan-2-yl)oxy)methyl)phenyl acetate (2r)

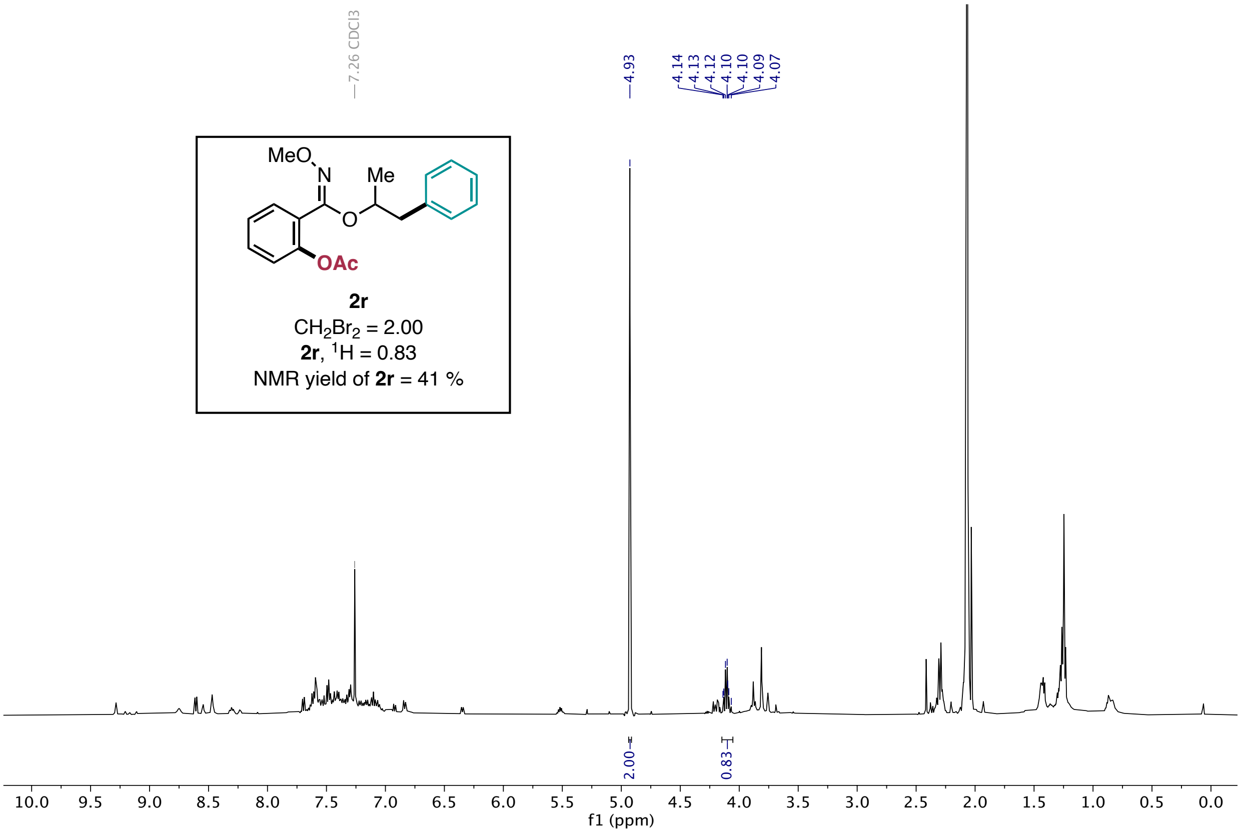

References:

1. Wu, Y.; Zhu, B.; Fan, H.; Bernard, H.; Hutton, C. A. Late-Stage Pd(II)-Catalyzed $C(sp^3)$−H Functionalization of Peptides Directed by a Removable, Backbone-Inserted Amidoxime Ether. *Angew. Chem. Int. Ed.* **2025**, *64*, e202423979.

2. He, B.-Q.; Gao, Y.; Wang, P.-Z.; Wu, H.; Zhou, H.-B.; Liu, X.-P.; Chen, J.-R. Dual Photoredox/Palladium-Catalyzed C–H Acylation of 2-Arylpyridines with Oxime Esters, *Synlett* **2021**, *32*, 373–377.

3. Ma, C.; Zhao, C.-Q.; Li, Y.-Q.; Zhang, L.-P.; Xu, X.-T.; Zhang, K.; Mei, T.-S**.** Palladium-Catalyzed C–H Activation/C–C Cross-Coupling Reactions via Electrochemistry. *Chem. Commun.* **2017**, *53*, 12189–12192.

4. Verma, P.; Richter, J. M.; Chekshin, N.; Qiao, J. X.; Yu, J.-Q. Palladium-Catalyzed $C(sp^3)$–H Functionalization of Free Carboxylic Acids. *J. Am. Chem. Soc.* **2020**, *142*, 5117–5125.

5. Nakafuku, K. M.; Fosu, S. C.; Nagib, D. A. Catalytic Alkene Difunctionalization via Imidate Radicals. *J. Am. Chem. Soc.* **2018**, *140*, 11202–11205.

6. Dandawate, M.; Choudhury, R.; Krishna, G. R.; Reddy, D. S. Total Synthesis and Absolute Configuration Determination of Ktedonoketone, a Benzenoid Metabolite from Thermophilic Bacterium. *Tetrahedron Lett.* **2020**, *61*, 152526.

7. Crich, D.; Li, M.; Jayalath, P. Dimethylthexylsilyl 2-Acetamido-3-O-allyl-2-deoxy-6-O-(4-methoxybenzyl)-β-d-glucopyranoside, Dimethylthexylsilyl 3,4,6-Tri-O-benzyl-β-d-mannopyranosyl-(1→4)-2-acetamido-3-O-allyl-2-deoxy-6-O-(4-methoxybenzyl)-β-d-glucopyranoside, and Dimethylthexylsilyl 2-O-(Benzylsulfonyl)-3,4,6-Tri-O-benzyl-β-d-mannopyranosyl-(1→4)-2-acetamido-3-O-allyl-2-deoxy-6-O-(4-methoxybenzyl)-β-d-glucopyranoside: Synthesis of Authentic Samples. *Carbohydr. Res.* **2009**, *344*, 140–144.

8. Gao, Y.; Li, G.; Liu, Y.; Lin, L.; Wang, D.; Liu, X.; Feng, X. Dichloroimidazolidinedione-Activated Beckmann Rearrangement of Ketoximes for Accessing Amides and Lactams. *J. Org. Chem.* **2018**, *83*, 2040–2049.

9. Hirata, Y.; Kimura, S.; Higashida, K.; Yoshino, T.; Matsunaga, S. Site-Selective $C(sp^3)$−H and Switchable $C(sp^3)$−H/$C(sp^2)$−H Functionalization Enabled by Electron-Deficient $Cp^*CF_3Ir(III)$ Catalyst and Photosensitizer. *Angew. Chem. Int. Ed.* ***2025***, *64*, e202421026.

10. Pilgrim, B. S.; Langer, T.; Johnson, C. N.; Taylor, R. J. K. Palladium-Catalyzed Enolate Arylation as a Key C–C Bond-Forming Reaction for the Synthesis of Isoquinolines. *Org. Biomol. Chem.* **2016**, *14*, 1065–1090.

11. Chu, Y.; Wang, R.; Zhang, X.; Gao, J.; Xu, Y. Asymmetric Reduction of Oxime Ethers Promoted by Chiral Spiroborate Esters with an O_3BN Framework. *J. Org. Chem.* **2006**, *71*, 3998–4001.

12. Hossain, M. D.; Kitamura, T. Unexpected, Drastic Effect of Triflic Acid on Oxidative Diacetoxylation of Iodoarenes by Sodium Perborate. A Facile and Efficient One-Pot Synthesis of (Diacetoxyiodo)arenes. *J. Org. Chem.* **2005**, *70*, 6984–6986.

13. Wang, Y.; Li, Y.; Yang, Y.; Yang, Y.; Zhang, Y.; Wang, J. Alkene Oxyalkylation Enabled by Merging Rhenium Catalysis with Hypervalent Iodine(III) Reagents via Decarboxylation. *J. Am. Chem. Soc.* **2013**, *135*, 18048–18051.
